# Prevalence of *BRCA1* and *BRCA2* pathogenic variants in a large, unselected breast cancer cohort

**DOI:** 10.1002/ijc.31841

**Published:** 2018-11-09

**Authors:** Jingmei Li, Wei Xiong Wen, Martin Eklund, Anders Kvist, Mikael Eriksson, Helene Nordahl Christensen, Astrid Torstensson, Svetlana Bajalica‐Lagercrantz, Alison M. Dunning, Brennan Decker, Jamie Allen, Craig Luccarini, Karen Pooley, Jacques Simard, Leila Dorling, Douglas F. Easton, Soo‐Hwang Teo, Per Hall, Åke Borg, Henrik Grönberg, Kamila Czene

**Affiliations:** ^1^ Human Genetics Genome Institute of Singapore Singapore Singapore; ^2^ Department of Surgery Yong Loo Lin School of Medicine, National University of Singapore Singapore Singapore; ^3^ Department of Medical Epidemiology and Biostatistics Karolinska Institutet Stockholm Sweden; ^4^ Cancer Research Malaysia, Sime Darby Medical Centre Subang Jaya Selangor Malaysia; ^5^ Division of Oncology and Pathology, Department of Clinical Sciences Lund University Lund Sweden; ^6^ AstraZeneca Nordic‐Baltic Södertälje Sweden; ^7^ Department of Oncology‐Pathology Karolinska Universitetssjukhuset Stockholm Sweden; ^8^ Centre for Cancer Genetic Epidemiology University of Cambridge Cambridge United Kingdom; ^9^ Cancer Genetics and Comparative Genomics Branch, National Human Genome Research Institute, National Institutes of Health Bethesda MD; ^10^ Department of Pathology Brigham and Women's Hospital Boston MA; ^11^ Genomics Center, Centre Hospitalier Universitaire de Québec‐Université Laval Research Center Canada Research Chair in Oncogenetics, Université Laval Quebec City Canada

**Keywords:** *BRCA1*, *BRCA2*, clinical testing, next‐generation sequencing, screening criteria, prediction, breast cancer

## Abstract

Breast cancer patients with *BRCA1/2*‐driven tumors may benefit from targeted therapy. It is not clear whether current *BRCA* screening guidelines are effective at identifying these patients. The purpose of our study was to evaluate the prevalence of inherited *BRCA1/2* pathogenic variants in a large, clinically representative breast cancer cohort and to estimate the proportion of *BRCA1/2* carriers not detected by selectively screening individuals with the highest probability of being carriers according to current clinical guidelines. The study included 5,122 unselected Swedish breast cancer patients diagnosed from 2001 to 2008. Target sequence enrichment (48.48 Fluidigm Access Arrays) and sequencing were performed (Illumina Hi‐Seq 2,500 instrument, v4 chemistry). Differences in patient and tumor characteristics of *BRCA1/2* carriers who were already identified as part of clinical *BRCA1/2* testing routines and additional *BRCA1/2* carriers found by sequencing the entire study population were compared using logistic regression models. Ninety‐two of 5,099 patients with valid variant calls were identified as *BRCA1/2* carriers by screening all study participants (1.8%). Only 416 study participants (8.2%) were screened as part of clinical practice, but this identified 35 out of 92 carriers (38.0%). Clinically identified carriers were younger, less likely postmenopausal and more likely to be associated with familiar ovarian cancer compared to the additional carriers identified by screening all patients. More *BRCA2* (34/42, 81.0%) than *BRCA1* carriers (23/50, 46%) were missed by clinical screening. In conclusion, *BRCA1/2* mutation prevalence in unselected breast cancer patients was 1.8%. Six in ten *BRCA* carriers were not detected by selective clinical screening of individuals.

## Abstract 2

**What's new?**

In order to provide personalized therapy to patients with pathogenic *BRCA1/2* variants, it's necessary to find them. Currently, patients are screened based on risk factors, such as family history. How many *BRCA1/2* carriers are missed? What if everyone were screened? Here, the authors sequenced DNA from more than 5,000 Swedish breast cancer patients looking for pathogenic *BRCA1/2* variants, and they found 92 carriers. Of these, only 35 had been identified by clinical screening. 60% of cancer‐causing *BRCA* variants had not been detected. This is one of the largest population‐based studies to date examining *BRCA1/2* prevalence.

## Introduction

Estimates of the prevalence of *BRCA1* or *BRCA2* germline pathogenic variants vary considerably depending on the technology used for mutation screening, population size and to what extent the genes are tested.^1^ Although *BRCA1/2* pathogenic variants are major determinants of hereditary breast cancers, women diagnosed with *BRCA1/2*‐associated breast cancer do not necessarily exhibit worse survival patterns than breast cancer patients without such pathogenic variants.^2^ On the contrary, patients diagnosed with *BRCA1/2*‐associated breast cancers have advantages in terms of treatment options when compared to patients with *BRCA1/2* wild‐type breast cancer (reviewed in Ref. 3). Evidence from clinical trials showed significantly greater sensitivity and higher response rate of *BRCA1/2*‐associated cancers to neoadjuvant and standard adjuvant chemotherapy than their wild‐type *BRCA1/2* counterparts.^3^ Treatment options for *BRCA1/2* breast cancers are also broadened with the introduction of new therapeutic agents, such as poly (ADP‐ribose) polymerase (*PARP*) inhibitors, which selectively target *BRCA1/2*‐deficient cancer cells.^4^, ^5^, ^6^, ^7^

Recommendation for counseling and genetic screening for *BRCA1/2* pathogenic variants is mainly based on personal and family history of breast and/or ovarian cancer, young age at disease onset, male breast cancer and multiple tumors (bilateral breast cancer or breast and ovarian cancer in the same patient).^8^ However, *BRCA* testing guidelines vary by region and country.^9^, ^10^ In Sweden, the Swedish Breast Cancer Group *BRCA1* and *BRCA2* screening criteria are used.^8^ A report by Nilsson *et al*. estimated that the Swedish *BRCA* testing criteria has an effectiveness of only 18% and concluded that clinical genetic testing criteria for *BRCA1* and *BRCA2* should be critically revised.^8^ As the effective identification of *BRCA1*/*2* germline pathogenic variants has potential to influence treatment decision and has implications for the family of the patients,^3^, ^4^, ^5^, ^6^, ^11^, ^12^ the pros and cons of testing all women diagnosed with breast cancer for such pathogenic variants need to be examined. In a large, clinically representative breast cancer cohort, we examined the prevalence and characteristics of *BRCA1/2* germline mutation carriers and compared our results with *BRCA* mutation carriers already identified through a national clinical *BRCA* screening program.

## Methods

### Study participants

All women under the age of 80 and diagnosed with breast cancer from 2001 to 2008 in Stockholm, Sweden were identified through the Stockholm‐Gotland Regional Breast Cancer quality register.^13^, ^14^ Women were invited to participate in the LIBRO1 study in 2009. In all, 5,715 women of the LIBRO1 study gave informed consent to the retrieval of data from medical records and national registers, answered a detailed questionnaire on background and lifestyle risk factors, and provided a blood specimen for genetic analysis.^13^, ^14^ Of these women, 5,125 were successfully genotyped in a large‐scale genotyping study on breast cancer risk (see **eTable 1** in **Data Supplement 1** for exclusion criteria, Supporting Information).^15^ Of these women, 5,122 had enough DNA remaining for targeted sequencing. The final analytical dataset comprised 5,099 samples which passed quality control. our study was approved by the Regional Ethical Review Board in Stockholm, Sweden (Karolinska Institutet, DNR2009/254–31/4).

### Patient characteristics

Self‐reported information on education level, age at menarche, body mass index (BMI), number of children, oral contraceptive use, hormone replacement therapy and details of family history of breast and ovarian cancer were obtained from the questionnaire. Patients were asked if their biological mothers and sisters have been diagnosed with breast or ovarian cancer, and if so, at what age. Mammograms were retrieved from radiology departments. Percent mammographic density was measured using an automated method described in Ref. 16. Information on whether the patients have an ovarian cancer or any nonbreast malignancy was retrieved *via* linkage to the Swedish Cancer Register using unique personal identity numbers of study participants (*personnummer*, 10 or 12 digit number used in Sweden to identify individuals).^17^

### Tumor characteristics

Tumor characteristics were retrieved from the Stockholm‐Gotland Regional Breast Cancer Quality Register^18^, ^19^ using unique personal identity numbers.^17^ Tumor size was measured in millimeters. Lymph node involvement was dichotomized into positive or negative. Estrogen receptor (ER) status was recorded as negative or positive in the registers, determined by radioimmunoassay or immunohistochemistry with cutoff values of more than 10% positive cells for IHC and more than 0 fmol/μg DNA for radioimmunoassay assays. The completeness of the registry data was 98% for tumor size and lymph node status and 80% for ER status. Information on grade (Nottingham histologic grade for invasive cancer and nuclear grade for cancer *in situ*) was available from 2004, with 93% completeness.^19^

Data on molecular markers were retrieved in 2015–2016 from medical and pathology records at treating hospitals (previously described in Ref. 20). HER2 status was dichotomized (positive/negative) in accordance with the Swedish Society of Pathology's guidelines: negative if protein expression showed 0 or 1+, or was higher with no confirmed gene amplification by FISH, and positive if FISH showed gene amplification.^20^ Proliferation marker Ki67 was measured according to contemporary guidelines and reported as percent staining (low if <20% and high otherwise).^20^ HER2 and Ki67 markers were not assessed, and thus not available in medical records, prior to 2005. Breast cancer subtype was assigned using a random forest algorithm (caret R package, v. 6.0.58) described in Ref. 20. The algorithm was trained to predict subtype based on a subset of individuals with PAM50 subtype derived from gene expression data (*n* = 237). Breast cancer subtype was then assigned to the remaining cases based on age at diagnosis, ER, PR, HER2 and Ki67 status.

### Targeted sequencing and data processing

Target‐enriched sequencing libraries of germline DNA from 5,122 breast cancer patients were prepared at the Centre for Cancer Genetic Epidemiology (University of Cambridge), as part of a larger effort that included samples from other cohorts. Briefly, target sequence enrichment was performed using 48.48 Fluidigm Access Arrays according to the manufacturer's protocol (Fluidigm, South San Francisco, CA). Fluidigm D3 assay design software was used to select primer pairs, which were multiplexed into pools selected for GC content and avoidance of off‐target primer‐primer and primer‐product complementarity (**eTable 2** in **Data Supplement 2**, Supporting Information). Target sequences were amplified with Illumina sequencing adaptors and one of 1,536 unique sample barcodes (supplied by Fluidigm, South San Francisco, CA). Robotic liquid handling and barcode plate identification were used in all steps of the library preparation process. The amplicon library was quantified with the KAPA Library Quantification Kit (KapaBiosystems, Boston, MA) and then sequenced on the Illumina Hi‐Seq 2,500 instrument using v4 chemistry, according to the manufacturer's protocol (Illumina, San Diego, CA). Each library was sequenced 2–3 times to provide sufficient coverage. Details on sequence data processing and quality control are shown in **eMethods** in **Data Supplement 1**, Supporting Information. A total of 5,099 samples had valid variant calls. The mean read depth across the coding sequences of *BRCA1* and *BRCA2* was 792.2 (standard deviation: 587.4) and 631 (standard deviation: 516), respectively. More than 90% of targeted bases had more than 15x coverage (94.8 [15.9] and 92.5 [20.4] for *BRCA1* and *BRCA2*, respectively).

### Definition of pathogenic variants

As described previously in Borg *et al*.,^21^ sequence variants were categorized based on their predicted effect on the mRNA and amino acid level and defined as pathogenic if they were (1) frameshift and nonsense variants with the exception of the *BRCA2* c.9976A > T (BIC: K3326X) and other variants located 3′ thereof (*n* = 105) and (2) all consensus splice acceptor or donor sequence sites, except those predicted to lead to naturally occurring in‐frame RNA isoforms that may rescue gene function.^22^ Public data on pathogenic *BRCA* variants (includes frameshift insertion/deletions, nonsense, splice sites and missense variants conclusively demonstrated to be pathogenic) that have been curated and classified by an international expert panel, the ENIGMA consortium, were also downloaded from http://brcaexchange.org/ (access date: Feb 22, 2017) for the annotation of the sequence data.

### Identification of women who have undergone *BRCA* testing in Sweden

Mutation screening for all oncogenetic clinics in Sweden (Lund, Stockholm, Uppsala, Göteborg, Linköping and Umeå) were conducted at the Department of Oncology, Lund University as part of a national *BRCA* testing program (**eMethods** in **Data Supplement 1**, Supporting Information). We cross‐referenced the personal identity numbers of all study participants in LIBRO1 with the *BRCA* testing unit at Lund University to identify women who have been tested for *BRCA1/2* pathogenic variants previously. The SweBRCA criteria are the only *BRCA1/2* testing criteria used in Sweden (**eTable 3** in **Data Supplement 1**, Supporting Information).^8^ Clinicians do not have any obligation to comply with the guidelines.^8^

### Statistical analysis

Predictor variables which include patient and tumor characteristics were described by the counts of each category and corresponding proportions. Binary logistic regression models were fitted for the dichotomous outcome (*BRCA1* [reference] and *BRCA2*) and multinomial logistic regression models were fitted for the three‐category outcome (*BRCA1*, *BRCA2* and non‐*BRCA* [reference category]), adjusting for age and year of diagnosis. Logistic regression models were also used to compare estimates (odds ratios [OR] and corresponding 95% confidence intervals [CI]) of patient and tumor characteristics between *BRCA1/2* carriers already identified among a subset of 416 patients screened as part of clinical *BRCA* testing routines and additional *BRCA1/2* carriers found by sequencing the entire study population (i.e., those not tested by the Swedish *BRCA* testing program).

## Results

The median time from date of diagnosis to study entry is 4.8 years (range: 1.3–9.2). The median age of breast cancer diagnosis of the study cohort was 59.6 years (range: 25.1–79.9). Nine of ten breast cancers were invasive (89.4%).

### Spectrum of *BRCA1* and *BRCA2* pathogenic variants

Of the 5,099 breast cancer patients, 92 (1.8%) were identified as *BRCA1/2* carriers (50 *BRCA1* carriers and 42 *BRCA2* carriers) and 5,007 were non‐*BRCA.*

**Figure 1:**
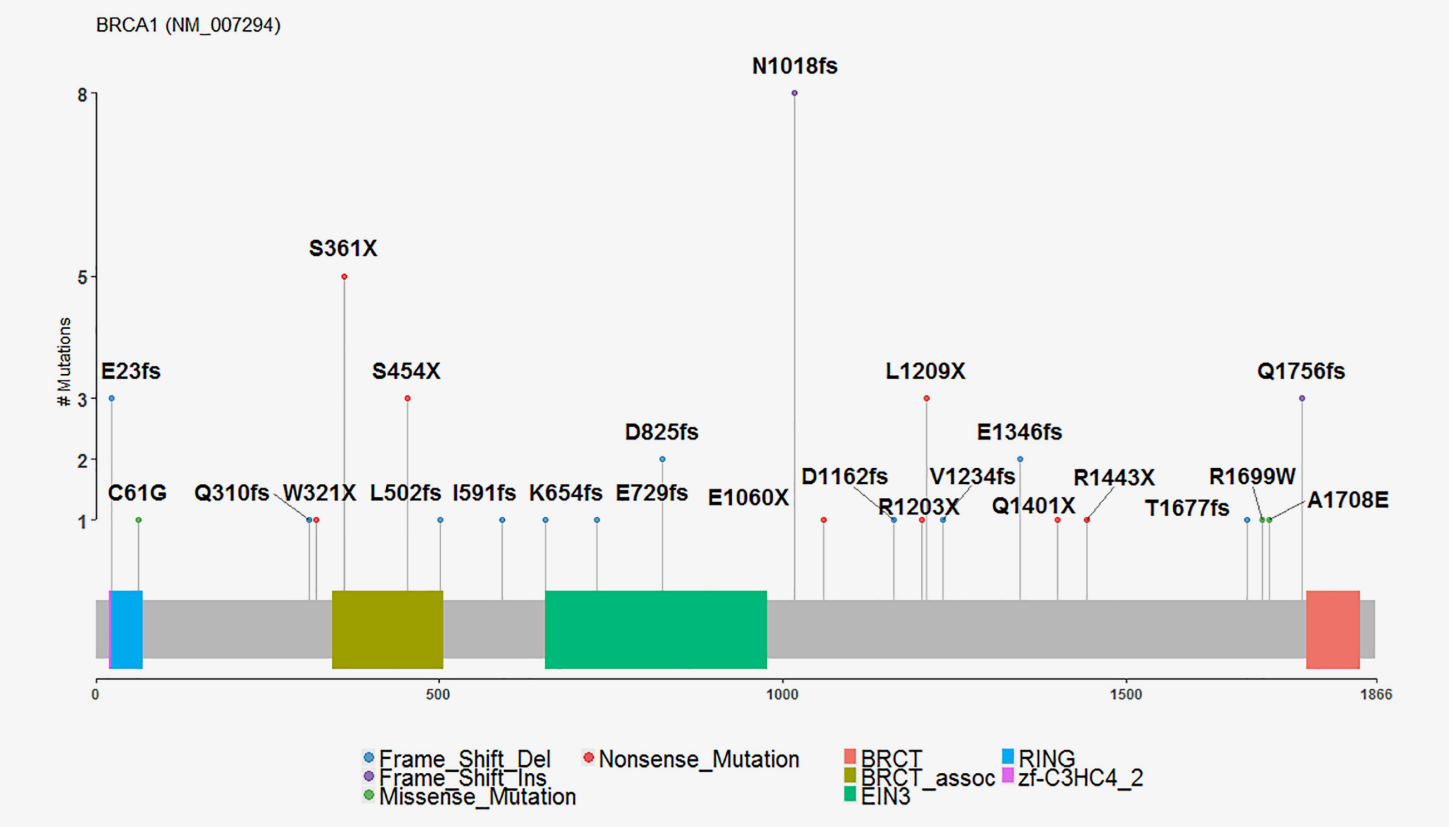
Mutation plot of *BRCA1.* Four and three splice variants for *BRCA1* (NM_007294.3) are not shown.

Among the 50 *BRCA1* carriers, there were 28 unique germline *BRCA1* pathogenic variants (11 frameshift deletions, 2 frameshift insertions, 8 truncating, 4 splice sites and 3 missense) (Fig. 1 and **eTable 4** in **Data Supplement 1**, Supporting Information). Frameshift insertions and deletions made up 26/50 (52%) of the *BRCA1* pathogenic variants. Exon 11 harbored 33/50 (66%) of the *BRCA1* pathogenic variants. The most common pathogenic variant was c.3048_3052dupTGAGA (*n* = 8), which is a founder mutation originating from the West coast of Sweden.^23^ Three other Swedish founder pathogenic variants were also identified (c.1082_1092del [*n* = 5], c.2475delC [*n* = 2]) and c.3626delT [*n* = 3]).^23^, ^24^, ^25^, ^26^

**Figure 2:**
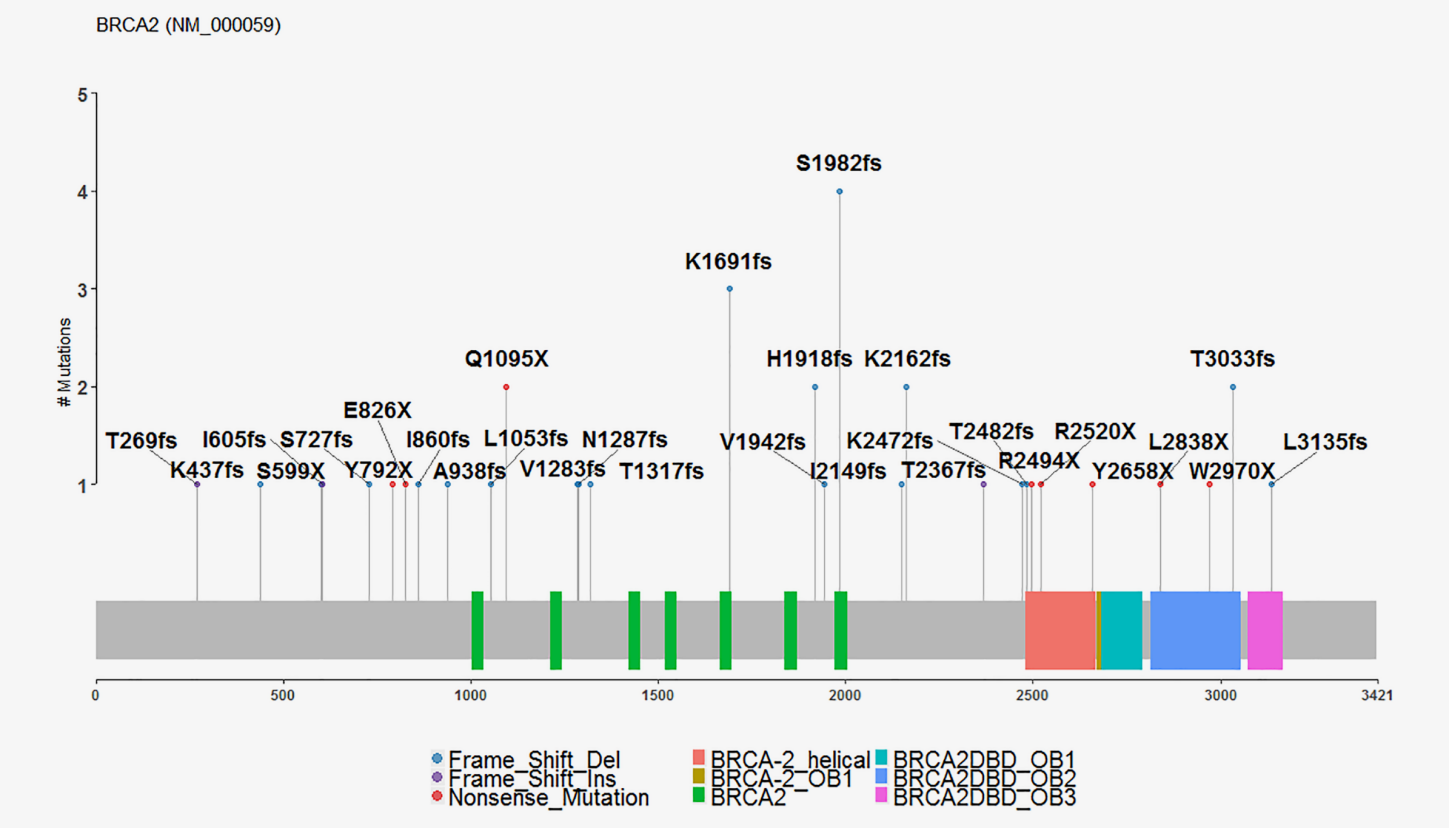
Mutation plot of *BRCA2.* Three splice variants for *BRCA2* (NM_000059.3) are not shown.

Among the 42 *BRCA2* carriers, there were 33 unique *BRCA2* pathogenic variants (18 frameshift deletions, 3 frameshift insertions, 9 truncating and 3 splice sites) (Fig. 2 and **eTable 5** in **Data Supplement 1,** Supporting Information). Over half of all *BRCA2* carriers (24/42, 57.1%) had a pathogenic variant on exon 11.

### Patient characteristics of *non‐BRCA*, *BRCA1* and *BRCA2 c*arriers

Half of the non‐*BRCA* women were at least 60 years old, compared to 26.0 and 33.3% for women with *BRCA1* and *BRCA2* pathogenic variants, respectively (**eTable 6** in **Data Supplement 1**, Supporting Information). In the crude analyses controlling for age and year of diagnosis, *BRCA1* and *BRCA2* carriers were more likely than non‐*BRCA* women to report family history of both breast (OR_*BRCA1 vs* non‐*BRCA*_: 4.00 [2.27–7.05] and OR_*BRCA2 vs* non‐*BRCA*_: 2.23 [1.17–4.26]) and family history of ovarian cancer (OR_*BRCA1 vs* non‐*BRCA*_: 7.53 [3.82–14.82] and OR_*BRCA2 vs* non‐*BRCA*_: 3.62 [1.50–8.71]) (**eTable 6** in **Data Supplement 1**, Supporting Information). *BRCA1* carriers, in particular, were also more likely to be also diagnosed with an ovarian cancer themselves (OR_*BRCA1 vs* non‐*BRCA*_: 28.02 [10.72–73.29] and OR_*BRCA2 vs* non‐*BRCA*_: 8.11 [1.87–35.24]) than non‐*BRCA* patients (**eTable 6** in **Data Supplement 1**, Supporting Information). *BRCA1* carriers were more likely to have a personal history of another malignant cancer in addition to their breast cancer than patients with non‐*BRCA* patients (OR_*BRCA1 vs* non‐*BRCA*_: 2.93 [1.37–6.27]). This association was driven by ovarian cancers (OR_*BRCA1 vs* non‐*BRCA*_ for all non‐breast and non‐ovarian malignancies: 0.83 [0.25–2.73]). *BRCA2* carriers were significantly less likely to be ever users of hormone replacement therapy (HRT) than non‐*BRCA* breast cancer patients (26.2% *vs* 53.8%) (**eTable 6** in **Data Supplement 1**, Supporting Information). In multivariable models shown in Table 1, all variables remained significantly associated, with the exception of personal history of any non‐breast malignancy.

**Table 1:** Odds ratio (OR) and corresponding 95% confidence intervals (CI) of predictors according to *BRCA* status

	*BRCA1 vs non‐BRCA* OR (95% CI)	*BRCA2 vs non‐BRCA* OR (95% CI)	*BRCA2 vs BRCA1* OR (95% CI)
*Model 1: Patient characteristics*			
Age at diagnosis: 50–59	**0.21 (0.10**–**0.45)**	0.78 (0.36–1.69)	**3.55 (1.05**–**11.97)**
Age at diagnosis: ≥60	**0.14 (0.06**–**0.31)**	0.55 (0.24–1.23)	**3.91 (1.11**–**13.84)**
Year of diagnosis: 2005–2008	1.68 (0.91–3.08)	1.03 (0.55–1.92)	0.90 (0.33–2.48)
HRT ever: Yes	1.08 (0.56–2.10	**0.36 (0.17**–**0.76)**	**0.31 (0.10**–**0.93)**
Familiy history of breast cancer: Yes	**3.57 (1.99**–**6.41)**	**2.08 (1.08**–**3.99)**	0.60 (0.24–1.55)
Familiy history of ovarian cancer: Yes	**6.99 (3.43**–**14.24)**	**3.57 (1.47**–**8.68)**	0.38 (0.11–1.35)
Personal history of ovarian cancer: Yes	**19.21 (5.89**–**62.72)**	**8.01 (1.61**–**39.94)**	0.49 (0.04–6.74)
Personal history of any malignant cancer (not breast): Yes	1.35 (0.52–3.54)	0.81 (0.26–2.56)	0.49 (0.07–3.59)
*Model 2: Tumor characteristics, adjusted for age and year of diagnosis*			
Detection mode: Interval	1.34 (0.38–4.79)	1.16 (0.45–3.03)	0.44 (0.05–3.50)
Detection mode: Clinical cancer in women without previous mammograms	2.61 (0.81–8.37)	0.66 (0.20–2.12)	0.35 (0.05–2.38)
Detection mode: Clinical cancer in women who had previous mammograms (i.e., interval > 24 months)	**3.54 (1.15**–**10.89)**	1.57 (0.63–3.94)	0.34 (0.06–2.02)
ER status: Negative	**5.19 (2.68**–**10.06)**	1.17 (0.48–2.87)	**0.22 (0.07**–**0.77)**
Grade: Intermediate‐differentiated	1.97 (0.24–16.23)	1.82 (0.52–6.34)	1.32 (0.10–18.26)
Grade: Poorly differentiated	7.11 (0.91–55.30)	1.55 (0.39–6.22)	0.36 (0.03–4.92)
Tumor size: ≥20	0.87 (0.48–1.59)	1.26 (0.67–2.39)	1.17 (0.37–3.76)
Nodal involvement: Yes	1.60 (0.79–3.27)	**2.54 (1.20**–**5.37)**	1.67 (0.43–6.51)
*Model 3: Breast cancer subtype, adjusted for age and year of diagnosis*			
Subtype: Luminal B	2.83 (0.54–14.77)	0.49 (0.06–3.73)	0.19 (0.01–2.60)
Subtype: HER2‐enriched	0.93 (0.11–8.07)	0.33 (0.04–2.52)	0.38 (0.02–8.07)
Subtype: Basal‐like	**40.07 (14.26**–**112.59)**	0.84 (0.11–6.43)	**0.02 (0.00**–**0.17)**

### Tumor characteristics of *non‐BRCA*, *BRCA1* and *BRCA2* carriers

In the crude analyses controlling for age and year of diagnosis, *BRCA2* carriers were in general not significantly different from non‐*BRCA* women in terms of tumor characteristics, with the exception of nodal involvement (OR_*BRCA2 vs* non‐*BRCA*_: 2.71 [1.31–5.62], **eTable 7** in **Data Supplement 1**, Supporting Information). On the contrary, tumors of *BRCA1* carriers were more aggressive than those of non‐*BRCA* breast cancer patients for all tumor characteristics examined (ER and PR status, grade, tumor size, nodal involvement and breast cancer subtype) except for the proportion of invasive tumors (**eTable 7** in **Data Supplement 1**, Supporting Information).

In multivariable multinomial models including all tumor characteristics that were significantly different between non‐*BRCA* and *BRCA1*‐positive breast cancer patients, only ER‐negativity remained significant (OR_*BRCA1 vs* non‐*BRCA*_: 5.19 [2.68–10.06]) (Table 1). ER status was also the only independent tumor characteristic that distinguished between *BRCA1* and *BRCA2* carriers (OR_*BRCA2 vs BRCA1*_: 0.22 [0.07–0.77]). This observation was mirrored in a separate multinomial model considering breast cancer subtypes, where *BRCA1* tumors were found to be 40 times more likely to be of the basal‐like subtype (OR_*BRCA1 vs* non‐*BRCA*_: 40.07 [14.26 to 112.59]). Only nodal involvement remained significant in the comparison between *BRCA2* and non‐*BRCA* breast cancer cases in the multivariable model (OR_*BRCA2 vs* non‐*BRCA*_: 2.54 [1.20–5.37) (Table 1).

### Comparison of *BRCA1/2* carriers identified *versus* not identified through clinical screening

**Figure 3:**
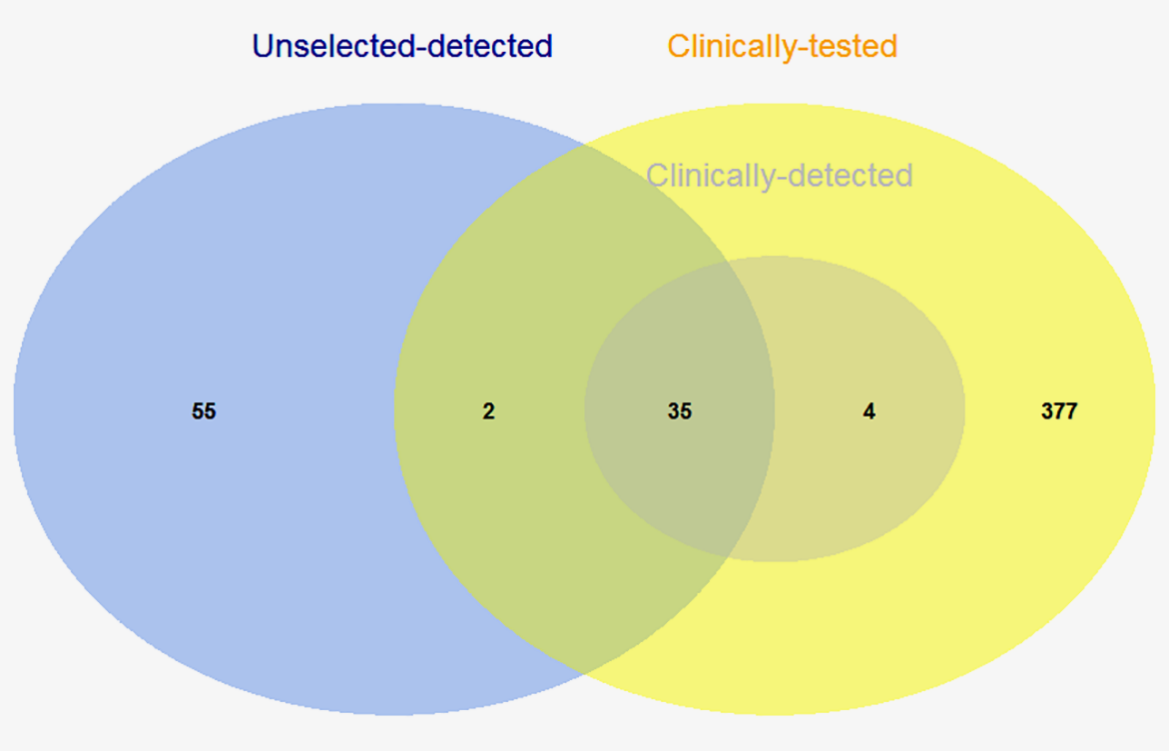
Overlap between women attending *BRCA* screening (clinically tested), *BRCA* carriers identified through selective clinical testing routine (clinically detected carriers) and *BRCA* carriers identified through screening all unselected LIBRO1 breast cancer patients (unselected‐detected). Of the 416 women who were clinically tested, 39 were found to be *BRCA1/2* carriers (39/416, 9.3%). Our study confirmed 35 of these pathogenic variants. Four pathogenic variants were missed (*BRCA1*: c.4186‐1785_4,358‐1667dup and c.4358‐1729_4986 + 736dup; *BRCA2*: c.7805 + 1538_8331 + 560del and c.9097_9098insT). By sequencing the entire Swedish study, we found 55 more carriers who were not screened as part of clinical routine. [Color figure can be viewed at wileyonlinelibrary.com]

Linkage with the Swedish *BRCA* register found 416 patients (8.2%) that were screened for pathogenic variants as part of routine clinical practice. Among these 416 women, clinical screening identified 39 carriers in the study cohort, of which our study confirmed 35 (Fig. 3). Four pathogenic variants were missed (*BRCA1*: c.4186‐1785_4,358‐1667dup and c.4358‐1729_4986 + 736dup; *BRCA2*: c.7805 + 1538_8331 + 560del and c.9097_9098insT) (Fig. 3). Three of these were large exonic deletions or duplications that the Fluidigm Access Array system is not suitable for detecting. This gives the Fluidigm Access Array method an estimated sensitivity of about 90%, or 97% when excluding large exonic variants.

Overall, 57/92 carriers (62.0%) were not already clinically identified: Two additional carriers were detected by the Fluidigm Access Array method among clinically screened patients (*BRCA2*: c.2578delA [confirmed by Sanger sequencing to be a false positive] and c.7443delT [missed carrier, screened with DHPLC and MLPA in 2008]); the remaining 55 out of 92 carriers (59.8%) identified by the Fluidigm Access Array method in the complete study cohort were never screened as part of clinical routine (Fig. 3).

More *BRCA2* (34/42, 80%) than *BRCA1* pathogenic variants (23/50, 46%) were missed by selectively testing only high‐risk individuals who were recommended for genetic testing and counseling (Table 2). Controlling for only year of diagnosis, *BRCA* carriers identified by clinical routine screening were younger (37.2% aged 50 years and above, compared to 73.7%), less likely to have experienced menopause (OR_identified *versus* not identified_: 0.17 [0.07–0.44]) and more likely to be associated with a family history of ovarian cancer (OR_identified *versus* not identified_: 3.11 [1.06–9.09]) (Table 2). Further adjustment for gene revealed a significant association with age at menarche (OR_identified *versus* not identified_: 2.99 [1.00–8.94]). There was also a trend between the likelihood of being identified as a carrier by selective testing and more children (Table 2). Tumors of *BRCA1/2* carriers identified by selective testing were more often detected clinically (OR_identified *versus* not identified_: 5.52 [1.38–22.18]), higher grade (OR_identified *versus* not identified_: 0.28 [0.08–0.92]), larger size (OR_identified *versus* not identified_: 2.48 [1.00–6.16]) and of a basal subtype (OR_identified *versus* not identified_: 6.07 [1.49–24.76]) (**eTable 8** in **Data Supplement 1**, Supporting Information). The differences observed for all tumor characteristics and selective testing detection did not remain significant after adjusting for gene.

**Table 2:** Frequency, odds ratio (OR) and corresponding 95% confidence intervals (CI) of patient characteristics among *BRCA* carriers identified *versus* not identified through selective clinical screening

Patient characteristic	Not identified by selective testing (*n* = 57)	Identified by selective testing (*n* = 35)	OR (95% CI)^1^	OR (95% CI)^2^	OR (95% CI)^3^
	*n* (%)	*n* (%)			
Gene, 1unadjusted					
*BRCA1*	23 (40.4)	27 (77.1)	1.00 (Reference)		
*BRCA2*	34 (59.6)	8 (22.9)	**0.20 (0.08**–**0.52)**		
Age at diagnosis, 1unadjusted					
<50	15 (26.3)	22 (62.9)	1.00 (Reference)		
50–59	20 (35.1)	8 (22.9)	**0.27 (0.10**–**0.78)**		
≥60	22 (38.6)	5 (14.3)	**0.15 (0.05**–**0.50)**		
Year of diagnosis, 1unadjusted					
2001–2004	26 (45.6)	12 (34.3)	1.00 (Reference)		
2005–2008	31 (54.4)	23 (65.7)	1.61 (0.67–3.84)		
Education					
University	29 (50.9)	21 (60.0)	1.00 (Reference)	1.00 (Reference)	1.00 (Reference)
Intermediate	12 (21.1)	9 (25.7)	1.06 (0.37–2.98)	1.40 (0.45–4.39)	2.08 (0.59–7.40)
Elementary	7 (12.3)	0 (0.0)	‐	‐	‐
Other	9 (15.8)	5 (14.3)	0.78 (0.23–2.68)	0.65 (0.17–2.46)	1.63 (0.35–7.66)
Age at menarche in years					
<13	21 (36.8)	7 (20.0)	1.00 (Reference)	1.00 (Reference)	1.00 (Reference)
≥13	36 (63.2)	28 (80.0)	2.17 (0.79–5.94)	**2.99 (1.00**–**8.94)**	**4.12 (1.19**–**14.26)**
Menopause status before breast cancer diagnosis					
Premenopause	14 (24.6)	23 (65.7)	1.00 (Reference)	1.00 (Reference)	1.00 (Reference)
Postmenopause	43 (75.4)	12 (34.3)	**0.17 (0.07**–**0.44)**	**0.17 (0.06**–**0.45)**	0.18 (0.03–1.25)
BMI in kg/m^2^					
<25	29 (50.9)	24 (68.6)	1.00 (Reference)	1.00 (Reference)	1.00 (Reference)
≥25	27 (47.4)	11 (31.4)	0.52 (0.21–1.27)	0.42 (0.16–1.12)	**0.32 (0.11**–**0.94)**
Missing	1 (1.8)	0 (0.0)			
Percentage mammographic density					
<25	22 (38.6)	10 (28.6)	1.00 (Reference)	1.00 (Reference)	1.00 (Reference)
≥25	16 (28.1)	14 (40.0)	1.97 (0.69–5.62)	1.54 (0.51–4.69)	0.93 (0.27–3.21)
Missing	19 (33.3)	11 (31.4)			
Number of children					
0	12 (21.1)	3 (8.6)	1.00 (Reference)	1.00 (Reference)	1.00 (Reference)
1	13 (22.8)	7 (20.0)	2.39 (0.49–11.65)	2.64 (0.50–13.83)	5.34 (0.84–33.79)
2	22 (38.6)	14 (40.0)	2.91 (0.68–12.53)	3.12 (0.68–14.24)	4.76 (0.89–25.43)
≥3	10 (17.5)	11 (31.4)	4.64 (1.00–21.66)	4.69 (0.93–23.60)	**10.55 (1.62**–**68.68)**
HRT ever					
No	34 (59.6)	25 (71.4)	1.00 (Reference)	1.00 (Reference)	1.00 (Reference)
Yes	21 (36.8)	10 (28.6)	0.61 (0.24–1.54)	0.45 (0.16–1.24)	0.84 (0.26–2.70)
Missing	2 (3.5)	0 (0.0)			
Oral contraceptives ever					
No	19 (33.3)	5 (14.3)	1.00 (Reference)	1.00 (Reference)	1.00 (Reference)
Yes	37 (64.9)	30 (85.7)	3.04 (1.01–9.15)	2.90 (0.91–9.24)	2.36 (0.71–7.85)
Missing	1 (1.8)	0 (0.0)			
Family history of breast cancer					
No	37 (64.9)	18 (51.4)	1.00 (Reference)	1.00 (Reference)	1.00 (Reference)
Yes	20 (35.1)	17 (48.6)	1.84 (0.77–4.39)	1.58 (0.63–3.99)	1.46 (0.54–3.90)
Family history of ovarian cancer					
No	50 (87.7)	24 (68.6)	1.00 (Reference)	1.00 (Reference)	1.00 (Reference)
Yes	7 (12.3)	11 (31.4)	**3.11 (1.06**–**9.09)**	2.87 (0.91–9.11)	3.41 (0.99–11.73)
Ovarian cancer					
No	51 (89.5)	33 (94.3)	1.00 (Reference)	1.00 (Reference)	1.00 (Reference)
Yes	6 (10.5)	2 (5.7)	0.60 (0.11–3.26)	0.37 (0.06–2.17)	0.46 (0.07–3.01)
Other malignant cancer					
No	48 (84.2)	31 (88.6)	1.00 (Reference)	1.00 (Reference)	1.00 (Reference)
Yes	9 (15.8)	4 (11.4)	0.76 (0.21–2.75)	0.54 (0.14–2.12)	0.66 (0.15–2.96)

1 Adjusted for year of diagnosis (2001–2004, 2005–2008).

2 Adjusted for year of diagnosis and gene (*BRCA1*, *BRCA2*).

3 Adjust for year of diagnosis, gene and age at diagnosis (<50, 50–59, ≥60).

## Discussion

*BRCA1/2* pathogenic variants were found in 1.8% of unselected breast cancer patients. In contrast to studies reporting *BRCA1/2* prevalence for a subset of high risk women,^27^, ^28^ the present sample reflects the general breast cancer population. None of the breast cancer risk factors examined differed between *BRCA1* and *BRCA2* carriers. However, *BRCA1* and *BRCA2* breast cancers differed in the proportions of patients with ER‐negative disease and basal‐like subtype. Six out of ten *BRCA1/2* carriers were not identified through genetic testing in the clinic.

*BRCA1* and *BRCA2* mutation frequencies in breast and ovarian cancer patients unselected for family history or age at onset are generally low (<1–7% for *BRCA1* and 1–3% for *BRCA2*).^29^ The combined *BRCA1/2* mutation frequency in a Swedish population of unselected breast cancer cases recruited from 1998 through 2000 in Stockholm has been previously estimated to be not more than 1% in the work by Margolin *et al*.^1^ In that study, screening for *BRCA1* pathogenic variants was limited to exon 11, which covers over half the coding region of *BRCA1.* 30 More than 70% of diagnosed pathogenic variants including four founder pathogenic variants in the Swedish population are known to be located on this exon.^31^, ^32^, ^33^ Prevalence of *BRCA2* pathogenic variants in the Swedish population was deemed by Margolin *et al*. to be negligible among unselected breast cancer patients due to the low frequency of such pathogenic variants even in high‐risk groups in the region.^1^ On the contrary, only 33 of 50 *BRCA1* pathogenic variants were identified on exon 11 in our study, thus suggesting that 34% of *BRCA1* carriers would have been missed if exon 11 alone were screened. Through testing the entire sequences of *BRCA1/2* genes with improved methodology and techniques, we estimate the combined prevalence of *BRCA1/2* pathogenic variants among unselected breast cancer patients in Sweden to be closer to 2%.

There are close to 2,000 known *BRCA1* germline pathogenic variants, many of which are loss‐of‐function frameshift pathogenic variants.^34^ Nine of 28 (32%) unique *BRCA1* and 6 of 33 (18%) unique *BRCA2* pathogenic variants were found to be recurrent in Swedish breast cancer patients (i.e., pathogenic variants that were found to occur in at least two unrelated individuals). The relatively low recurrent mutation frequency, including that of Swedish founder pathogenic variants, would mean that screening of selected pathogenic variants alone may not be a sensitive approach in this population as majority of *BRCA1* and *BRCA2* carriers will have been missed. While *BRCA1* pathogenic variants confer a more aggressive tumor phenotype, *BRCA2* pathogenic variants typically resemble sporadic breast cancer.^35^ There is good agreement between our observed results regarding the tumor characteristic differences between *BRCA1/2* and non‐*BRCA* breast cancer cases and what has been previously reported in literature. It has been observed by others that tumors in *BRCA1* carriers more frequently exhibited high mitotic count, high grade, ER and PR negativity.^36^, ^37^, ^38^ A large proportion of *BRCA1* mutation cases (~80%) have also been documented to be triple negative and basal‐like breast cancers.^36^, ^37^, ^38^ In a Swedish study where 54 female breast cancer patients from 22 families with *BRCA2* germ line pathogenic variants from Sweden and Denmark were compared to 214 age‐ and date of diagnosis‐matched controls identified among breast cancer patients from South Sweden, *BRCA2*‐associated cases were more often node‐positive than non‐*BRCA* cases.^39^ Other than nodal involvement, *BRCA2*‐associated breast carcinomas were generally associated with less aggressive tumor characteristics than *BRCA1* cancers, and were more likely to be hormone‐related.^37^, ^38^

Thirty‐eight percent of *BRCA1/2* carriers were identified through selective clinical testing of 8.2% of breast cancer patients. Grindedal *et al*. evaluated the results of *BRCA1/2* testing in South‐Eastern Norway and found that 65% of the *BRCA1/2* carriers would have been missed if using age of onset below 40 or triple negative breast cancer as criteria for testing.^40^ It is also conceivable that, due to an emphasis on disease family history in current guidelines, a smaller family size may compromise the identification of high risk individuals who would otherwise benefit from genetic testing.^41^ In a Swedish retrospective study by Nilsson *et al*. where all breast cancer patients were tested, it was found that while 65% of the *BRCA1/2* carriers fulfilled Swedish criteria for testing, only 18% had been identified in regular clinical routine.^8^ Other factors such as varying compliance with guidelines for the recommendation of *BRCA* testing by clinicians will lead to even more *BRCA1/2* carriers being missed. It may thus be of benefit to test all newly diagnosed breast cancers in light of available targeted therapy options.

To our knowledge, this is the largest population‐based breast cancer testing study for *BRCA1/2* published outside of founder populations. Despite the richness of the data which encompasses patient and tumor, some risk groups were too small to be examined with adequate statistical power (e.g., benign breast disease). The Swedish health care system is mainly government‐funded and decentralized, making it possible to identify all women who went for clinical *BRCA* testing. Nonetheless, private health care also exists, and some *BRCA1/2* carriers may have been identified by commercial testing outside the public sector. However, the number of patients tested outside of the national *BRCA* testing program is likely negligible during the period 2001–2008.^8^ It should be also noted that the Fluidigm Access Array method used cannot detect large rearrangements and has a sensitivity of ~90%, hence further analytical validity studies are needed. More sensitive methods and the universal *BRCA* testing of newly breast cancer patients will help to increase the number of women getting the best treatment for their disease.

In summary, *BRCA1/2* pathogenic variants were found in 1.8% of an unselected Swedish breast cancer cohort. Six out of ten *BRCA* carriers were not identified through selective clinical testing routines. Our results give fruitful information for further decisions of *BRCA* testing for all breast cancer patients at time of diagnosis. The presented data can be a starting point for further studies dealing with issues such as cost effectiveness of screening patients with different tumor characteristics and patient health attitudes.

## Supporting information

Data Supplement 1

Data Supplement 2
