# Supplementary material for: Prevalence of BRCA1 and BRCA2 pathogenic variants in a large, unselected breast cancer cohort
Source: Int J Cancer. 2018 Nov 9;144(5):1195–204. doi: 10.1002/ijc.31841 (PMC6320715; doi:10.1002/ijc.31841)
Supplement: Supplementary file 1 — Data Supplement 1 [file IJC-144-1195-s001.docx]

**Prevalence of *BRCA1* and *BRCA2* pathogenic variants in a
large, unselected breast cancer cohort**

**DATA SUPPLEMENT 1**

[**eTable 1.** Exclusion criteria for genotyping experiment 2](#_Toc508713019)

[**eMETHODS** 3](#_Toc508713020)

[**eTable 3**. Swedish Breast Cancer Group criteria for recommending *BRCA1/2* testing 4](#_Toc508713022)

[**eTable 4**. Description of *BRCA1* (NM_007294.3) pathogenic variants. 5](#_Toc508713023)

[**eTable 5**. Description of *BRCA2* (NM_000059.3) pathogenic variants. 6](#_Toc508713024)

[**eTable 6.** Frequency, odds ratio (OR) and corresponding 95% confidence intervals (CI) of patient characteristics according to *BRCA* status. 7](#_Toc508713025)

[**eTable 7.** Frequency, odds ratio (OR) and corresponding 95% confidence intervals (CI) of tumor characteristics according to *BRCA* status.**.** 9](#_Toc508713026)

[**eTable 8.** Frequency, odds ratio (OR) and corresponding 95% confidence intervals (CI) of tumor characteristics among *BRCA* carriers identified versus not identified through selective clinical screening.**.** * Adjusted for year of diagnosis (2001-2004, 2005-2008) 11](#_Toc508713027)

[**References** 14](#_Toc508713030)

#

# **eTable 1.** Exclusion criteria for genotyping experiment (Michailidou *et al.* ^1^). Of the 5,715 women who consented to genetic analyses of their blood samples, genotyping was successfully performed for 5,125 women. Of these, 5,122 had enough DNA remaining for targeted sequencing.

| **Exclusion criteria for genotyping experiment** | ***n*** |
| --- | --- |
| Concordant replicate - exclude lower call rate | 116 |
| Cryptic Duplicate | 7 |
| Extreme heterozygous | 34 |
| Call rate (<95%) | 8 |
| Male | 1 |
| Non-European | 114 |
| Phenotype data excluded | 177 |
| Relative pairs with different status | 9 |
| Relative pairs, exclude lower call rate | 39 |
| Unclear whether consented when data released in Jan 2012 | 69 |
| Study duplicates with KARBAC sample | 4 |
| Genotype not received | 12 |

# **eMETHODS**

**Details on targeted sequencing methodology used by the University of Cambridge (Fluidigm Access Array method)**

*Targeted sequencing*

Target-enriched sequencing libraries of germline DNA from 5,122 breast cancer patients were prepared at the Centre for Cancer Genetic Epidemiology (University of Cambridge). Data used in this study were part of a larger effort that included samples from other cohorts, as well as coding sequences and intron/exon boundaries for a total of 31 known or suspected breast cancer susceptibility genes, including *BRCA1* and *BRCA2*. Assay design was conducted as previously described ^2^. See e**Table 2** (**Data Supplement 2**) for primer sequences and amplicon details.

Briefly, target sequence enrichment was performed using 48.48 Fluidigm Access Arrays according to the manufacturer’s protocol (Fluidigm, South San Francisco, California, USA). Fluidigm D3 assay design software was used to select primer pairs, which were multiplexed into pools selected for GC content and avoidance of off-target primer-primer and primer-product complementarity. Target sequences were amplified with Illumina sequencing adaptors and one of 1,536 unique sample barcodes (supplied by Fluidigm, South San Francisco, California, USA). Robotic liquid handling and barcode plate identification were used in all steps of the library preparation process. Each library of amplicons (e**Table 2**, **Data Supplement 2**) for 1,536 samples was quantified with the KAPA Library Quantification Kit (KapaBiosystems, Boston, Massachusetts, USA) and then sequenced on the Illumina Hi-Seq 2500 instrument using v4 chemistry, according to the manufacturer’s protocol (Illumina, San Diego, California, USA). Each library was sequenced 2-3 times to provide sufficient coverage.

*Sequence data processing and quality control*

Raw data in FASTQ format was received from the University of Cambridge. Paired-end sequencing reads were aligned to the human genome reference sequence (hg19) using Burrows-Wheeler Aligner (version 0.7.12 ^3^). Aligned reads in SAM format were converted to BAM format and subsequently merged for each sample using SAMtools (version 1.1 ^4^). Read groups were assigned using Picard (version 1.119; http://broadinstitute.github.io/picard). Genome Analysis Toolkit (GATK version 3.7.0; https://software.broadinstitute.org/gatk/) was used for local insertion/deletion (indel) realignment and base quality score recalibration, variant calling, SNP and indel parsing and for deriving quality and depth metrics ^5^. The mean read depth across the coding sequences of *BRCA1* and *BRCA2* was 792.2 (standard deviation: 587.4) and 631 (standard deviation: 516), respectively. More than 90% of targeted bases had more than 15x coverage (94.8 [15.9] and 92.5 [20.4] for *BRCA1* and *BRCA2*, respectively).

Genetic variants were called with Unified Genotyper using the default parameters except –mindelFrac 0.05. SNPs and indels with low variant confidence/quality by depth (QD<2) and low approximate read depth (DP<10) were removed. Filter-based annotation of variants were performed using ANNOVAR ^6^. A total of 5,099 samples with valid variant calls were included in the final analytical dataset.

**Details on targeted sequencing methodology used by the Department of Oncology, Lund University (modified SureSelect hybrid selection method)**

The clinical mutation screening was performed using the most sensitive methods available for comprehensive detection of all classes of genetic variants known to affect *BRCA1* and *BRCA2*. Targeted sequencing libraries were prepared using a modified SureSelect hybrid selection method and a custom panel targeting 64 genes including complete *BRCA1* and *BRCA2* loci (exons and introns) and 100kb up- and downstream. Specificity was ensured by confirming all variants with Sanger sequencing on an independent DNA extraction from the patient blood sample. Paired-end sequencing of the libraries was performed on a HiSeq 2500 (2x100bp) to an average depth of ~400 reads. Until 2016, this was complemented with multiple ligation-dependent probe amplification (MLPA) for detection of deletions and duplications affecting one or more complete exons. The lab now has validated bioinformatic methods for detecting these variants directly from the sequencing data. Sensitivity estimated using a large collection of positive control samples including all classes of known pathogenic variants is 100%. Before 2010, denaturing high performance liquid chromatography (DHPLC) and MLPA was used. Together, the DHPLC and MLPA have a stated sensitivity of 95%. Many of the samples tested before 2010 have been screened again using the latest methods.

# **eTable 3**. Swedish Breast Cancer Group criteria for recommending *BRCA1/2* testing.

| **Criterion** | **Number meeting criterion** |
| --- | --- |
| Three cases of breast cancer in first degree relatives, or second degree relatives thought a male, with at least one diagnosed ≤50y, and/or ovarian cancer (regardless of age) | 79 |
| Two cases of breast cancer or ovarian cancer in first degree relatives, or second degree relatives thought a male, with at least one case of breast cancer diagnosed ≤40y, or two cases of ovarian cancer (regardless of age) | 113 |
| One case of breast cancer ≤35y | 99 |
| One case of triple-negative breast cancer ≤40y | 20 |
| One case of male breast cancer | NA |
| Breast cancer and ovarian cancer in one individual | 44 |
| Cases of bilateral breast cancer, prostate cancer, and pancreatic cancer may strengthen the indication for screening of pathogenic variants in BRCA1 and BRCA2, but are not defined in any specific criterion | NA |
| Total | 298 |

# **eTable 4**. Description of *BRCA1* (NM_007294.3) pathogenic variants.

| **Exon** | **cDNA Change** | **AA Change** | **Variant Classification** | **BIC Nomenclature** | **Note** | ***n*** |
| --- | --- | --- | --- | --- | --- | --- |
| 2 | c.68_69delAG | p.E23fs | frameshift deletion | 185_186delAG,185delAG,187delAG | Founder mutation in Ashkenazi Jews ^7^ | 3 |
| 5 | c.181T>G | p.C61G | nonsynonymous SNV | 300T>G | Common mutation in Europe ^8^ | 1 |
| 7 | c.302-2A>G | - | splice site | - | - | 1 |
| 11 | c.930delG | p.Q310fs | frameshift deletion | 1049delG | - | 1 |
| 11 | c.962G>A | p.W321* | stopgain | W321X | - | 1 |
| 11 | c.1082_1092delCAGAGAATCCT | p.S361* | stopgain | 1201del11 | Founder mutation common in Southern Sweden ^9^ | 5 |
| 11 | c.1360_1361delAG | p.S454* | stopgain | 1479delAG | - | 3 |
| 11 | c.1504_1508delTTAAA | p.L502fs | frameshift deletion | 1623_1627delTTAAA | - | 1 |
| 11 | c.1772delT | p.I591fs | frameshift deletion | 1891delT | - | 1 |
| 11 | c.1961delA | p.K654fs | frameshift deletion | 2080delA | - | 1 |
| 11 | c.2184delA | p.E729fs | frameshift deletion | - | - | 1 |
| 11 | c.2475delC | p.D825fs | frameshift deletion | 2594delC | Swedish BRCA1 founder mutation ^10^ | 2 |
| 11 | c.3048_3052dupTGAGA | p.N1018fs | frameshift insertion | 3166insTGAGA, p.Asn1018fs | Founder mutation originating from West Coast of Sweden ^8, 11^ | 8 |
| 11 | c.3178G>T | p.E1060* | stopgain | E1060X | - | 1 |
| 11 | c.3485delA | p.D1162fs | frameshift deletion | 3604delA | Founder mutation in Finland ^8^ | 1 |
| 11 | c.3607C>T | p.R1203* | stopgain | 3726C>T | - | 1 |
| 11 | c.3626delT | p.L1209* | stopgain | 3745delT | Founder mutation originating in Northern Sweden ^8^ | 3 |
| 11 | c.3700_3704delGTAAA | p.V1234fs | frameshift deletion | 3819_3823delGTAAA | Frequent mainly in Middle and Eastern Europe and Canada ^12^ | 1 |
| 11 | c.4035delA | p.E1346fs | frameshift deletion | 4154delA | Common mutation in Poland and Latvia ^8^ | 2 |
| 13 | c.4201C>T | p.Q1401* | stopgain | - | - | 1 |
| 13 | c.4327C>T | p.R1443* | stopgain | 4446C>T | - | 1 |
| 17 | c.5030_5033delCTAA | p.T1677fs | frameshift deletion | 5149del4,5147del4,5146del4 | - | 1 |
| 18 | c.5075-2A>C | - | splice site | IVS17-2A>C | - | 1 |
| 18 | c.5095C>T | p.R1699W | nonsynonymous SNV | 5214C>T | - | 1 |
| 18 | c.5123C>A | p.A1708E | nonsynonymous SNV | 5242C>A | - | 1 |
| 19 | c.5153-1G>C | - | splice site | IVS18-1G>C | - | 2 |
| 20 | c.5266dupC | p.Q1756fs | frameshift insertion | 5382_5383insC,5382insC,5383insC,5384insC,5385insC | Founder mutation in Russia ^13^ | 3 |
| 21 | c.5278-2A>T | - | splice site | - | - | 1 |

# **eTable 5**. Description of *BRCA2* (NM_000059.3) pathogenic variants.

| **Exon** | **cDNA Change** | **AA Change** | **Variant Classification** | **BIC Nomenclature** | **Note** | ***n*** |
| --- | --- | --- | --- | --- | --- | --- |
| 10 | c.805dupA | p.T269fs | frameshift insertion | 1033insA,p.Thr269fs | - | 1 |
| 10 | c.1310_1313delAAGA | p.K437fs | frameshift deletion | 1537_1540delAAAG | - | 1 |
| 10 | c.1796_1800delCTTAT | p.S599* | stopgain | 2024_2028delCTTAT | - | 1 |
| 10 | c.1813dupA | p.I605fs | frameshift insertion | 2041_2042insA | - | 1 |
| 11 | c.2179delT | p.S727fs | frameshift deletion | - | - | 1 |
| 11 | c.2376C>G | p.Y792* | stopgain | - | - | 1 |
| 11 | c.2476G>T | p.E826* | stopgain | - | - | 1 |
| 11 | c.2578delA | p.I860fs | frameshift deletion | - | - | 1 |
| 11 | c.2808_2811delACAA | p.A938fs | frameshift deletion | 3036_3039delACAA | - | 1 |
| 11 | c.3157_3163delTTAGATA | p.L1053fs | frameshift deletion | - | - | 1 |
| 11 | c.3283C>T | p.Q1095* | stopgain | - | - | 2 |
| 11 | c.3847_3848delGT | p.V1283fs | frameshift deletion | 4075_4076delGT | - | 1 |
| 11 | c.3860delA | p.N1287fs | frameshift deletion | 4088delA,4082delA | - | 1 |
| 11 | c.3950delC | p.T1317fs | frameshift deletion | - | - | 1 |
| 11 | c.5073delA | p.K1691fs | frameshift deletion | 5301delA | - | 3 |
| 11 | c.5754_5755delTA | p.H1918fs | frameshift deletion | - | - | 2 |
| 11 | c.5823delA | p.V1942fs | frameshift deletion | 6051delA | - | 1 |
| 11 | c.5946delT | p.S1982fs | frameshift deletion | 6174delT | Founder mutation in Ashkenazi Jews ^8^ | 4 |
| 11 | c.6444delT | p.I2149fs | frameshift deletion | - | - | 1 |
| 11 | c.6486_6489delACAA | p.K2162fs | frameshift deletion | 6714_6717delACAA | - | 2 |
| 14 | c.7097dupT | p.T2367fs | frameshift insertion | - | - | 1 |
| 14 | c.7414_7415delAA | p.K2472fs | frameshift deletion | 7642delAA | - | 1 |
| 15 | c.7443delT | p.T2482fs | frameshift deletion | 7671delT | - | 1 |
| 15 | c.7480C>T | p.R2494* | stopgain | 7708C>T | - | 1 |
| 15 | c.7558C>T | p.R2520* | stopgain | 7786C>T | - | 1 |
| 16 | c.7618-1G>A | - | splice site | IVS15-1G>A | - | 1 |
| 17 | c.7974C>G | p.Y2658* | stopgain | Y2658X | - | 1 |
| 19 | c.8332-1G>A | - | splice site | - | - | 1 |
| 20 | c.8513T>G | p.L2838* | stopgain | 8741T>G, p.Leu2838X | - | 1 |
| 22 | c.8910G>A | p.W2970* | stopgain | 9138G>A (W-X),p.Trp2970X | - | 1 |
| 23 | c.9097delA | p.T3033fs | frameshift deletion | - | - | 2 |
| 24 | c.9118-2A>G | - | splice site | IVS23-2A>G | - | 1 |
| 25 | c.9403delC | p.L3135fs | frameshift deletion | 9631delC | - | 1 |

# **eTable 6.** Frequency, odds ratio (OR) and corresponding 95% confidence intervals (CI) of patient characteristics according to *BRCA* status. *Adjusted for age (<50, 50-59, ≥60) and year of diagnosis (2001-2004 and 2005-2008).

| **Patient characteristic** | **Non-*BRCA* (*n*=5,007)** | ***BRCA1* (*n*=50)** | ***BRCA2* (*n*=42)** | ***BRCA1 vs non-BRCA OR (95% CI)**** | ***BRCA2 vs  non-BRCA OR (95% CI)**** | ***BRCA2 vs BRCA1 OR (95% CI)**** |
| --- | --- | --- | --- | --- | --- | --- |
| Age at study entry, years (mean, SD) | 63.4 (9.9) | 54.9 (12.6) | 58.6 (9.4) |  |  |  |
|  |  |  |  |  |  |  |
| Age at diagnosis, years (mean, SD) | 58.6 (9.9) | 50.3 (12.4) | 54.0 (9.5) |  |  |  |
|  |  |  |  |  |  |  |
| Age at diagnosis, years (unadjusted) |  |  |  |  |  |  |
| <50 | 887 (17.7) | 24 (48.0) | 13 (31.0) | 1.00 (Reference) | 1.00 (Reference) | 1.00 (Reference) |
| 50-59 | 1666 (33.3) | 13 (26.0) | 15 (35.7) | **0.29 (0.15 to 0.57)** | 0.61 (0.29 to 1.30) | 2.13 (0.78 to 5.81) |
| ≥60 | 2454 (49.0) | 13 (26.0) | 14 (33.3) | **0.20 (0.10 to 0.39)** | **0.39 (0.18 to 0.83)** | 1.99 (0.72 to 5.47) |
|  |  |  |  |  |  |  |
| Year of diagnosis (unadjusted) |  |  |  |  |  |  |
| 2001-2004 | 2325 (46.4) | 19 (38.0) | 19 (45.2) | 1.00 (Reference) | 1.00 (Reference) | 1.00 (Reference) |
| 2005-2008 | 2682 (53.6) | 31(62.0) | 23 (54.8) | 1.41 (0.80 to 2.51) | 1.05 (0.57 to 1.93) | 0.74 (0.32 to 1.71) |
|  |  |  |  |  |  |  |
| Education |  |  |  |  |  |  |
| University | 2113 (42.2) | 29 (58.0) | 21 (50.0) | 1.00 (Reference) | 1.00 (Reference) | 1.00 (Reference) |
| Intermediate | 1116 (22.3) | 9 (18.0) | 12 (28.6) | 0.60 (0.28 to 1.27) | 1.09 (0.53 to 2.23) | 1.51 (0.52 to 4.42) |
| Elementary | 753 (15.0) | 3 (6.0) | 4 (9.5) | 0.48 (0.14 to 1.63) | 0.66 (0.22 to 1.96) | 1.10 (0.20 to 6.02) |
| Other | 961 (19.2) | 9 (18.0) | 5 (11.9) | 1.05 (0.48 to 2.29) | 0.63 (0.23 to 1.72) | 0.50 (0.13 to 1.89) |
| Missing | 64 (12.8) | 0 (0.0) | 0 (0.0) |  |  |  |
|  |  |  |  |  |  |  |
| Age at menarche, years |  |  |  |  |  |  |
| <13 | 1592 (31.8) | 17 (34.0) | 11 (26.2) | 1.00 (Reference) | 1.00 (Reference) | 1.00 (Reference) |
| ≥13 | 3263 (65.2) | 33 (66.0) | 31 (73.8) | 1.12 (0.62 to 2.03) | 1.52 (0.76 to 3.06) | 1.51 (0.58 to 3.89) |
| Missing | 152 (3.0) | 0 (0.0) | 0 (0.0) |  |  |  |
|  |  |  |  |  |  |  |
| BMI, kg/m^2^ |  |  |  |  |  |  |
| <25 | 2644 (52.8) | 28 (56.0) | 25 (59.5) | 1.00 (Reference) | 1.00 (Reference) | 1.00 (Reference) |
| ≥25 | 2275 (45.4) | 22 (44.0) | 16 (38.1) | 1.03 (0.58 to 1.81) | 0.79 (0.42 to 1.49) | 0.84 (0.35 to 2.00) |
| Missing | 88 (1.8) | 0 (0.0) | 1 (2.4) |  |  |  |
|  |  |  |  |  |  |  |
| Percentage mammographic density |  |  |  |  |  |  |
| <25 | 2362 (47.2) | 15 (30.0) | 17 (40.5) | 1.00 (Reference) | 1.00 (Reference) | 1.00 (Reference) |
| ≥25 | 1507 (30.1) | 20 (40.0) | 10 (23.8) | 1.34 (0.66 to 2.75) | 0.73 (0.32 to 1.65) | 0.52 (0.18 to 1.53) |
| Missing | 1138 (22.7) | 15 (30.0) | 15 (35.7) |  |  |  |
|  |  |  |  |  |  |  |
| Number of children |  |  |  |  |  |  |
| 0 | 814 (16.3) | 8 (16.0) | 7 (16.7) | 1.00 (Reference) | 1.00 (Reference) | 1.00 (Reference) |
| 1 | 887 (17.7) | 10 (20.0) | 10 (23.8) | 1.26 (0.49 to 3.21) | 1.37 (0.52 to 3.62) | 0.96 (0.24 to 3.85) |
| 2 | 2145 (42.8) | 19 (38.0) | 17 (40.5) | 0.98 (0.43 to 2.26) | 0.96 (0.40 to 2.33) | 0.95 (0.27 to 3.31) |
| ≥3 | 1130 (22.6) | 13 (26.0) | 8 (19.0) | 1.36 (0.56 to 3.31) | 0.89 (0.32 to 2.48) | 0.64 (0.16 to 2.54) |
| Missing | 31 (0.6) | 0 (0.0) | 0 (0.0) |  |  |  |
|  |  |  |  |  |  |  |
| HRT ever |  |  |  |  |  |  |
| No | 2208 (44.1) | 30 (60.0) | 29 (69.0) | 1.00 (Reference) | 1.00 (Reference) | 1.00 (Reference) |
| Yes | 2694 (53.8) | 20 (40.0) | 11 (26.2) | 1.02 (0.53 to 1.94) | **0.36 (0.17 to 0.75)** | 0.36 (0.13 to 1.00) |
| Missing | 105 (2.1) | 0 (0.0) | 2 (4.8) |  |  |  |
|  |  |  |  |  |  |  |
| Oral contraceptives ever |  |  |  |  |  |  |
| No | 1285 (25.7) | 11 (22.0) | 13 (31.0) | 1.00 (Reference) | 1.00 (Reference) | 1.00 (Reference) |
| Yes | 3663 (73.2) | 39 (78.0) | 28 (66.7) | 0.87 (0.43 to 1.75) | 0.58 (0.29 to 1.16) | 0.67 (0.26 to 1.75) |
| Missing | 59 (1.1) | 0 (0.0) | 1 (2.4) |  |  |  |
|  |  |  |  |  |  |  |
| Ovarian cancer |  |  |  |  |  |  |
| No | 4971 (99.3) | 44 (88.0) | 40 (95.2) | 1.00 (Reference) | 1.00 (Reference) | 1.00 (Reference) |
| Yes | 36 (0.7) | 6 (12.0) | 2 (4.8) | **28.02 (10.72 to 73.29)** | **8.11 (1.87 to 35.24)** | 0.27 (0.05 to 1.50) |
|  |  |  |  |  |  |  |
| Any malignant cancer, not breast |  |  |  |  |  |  |
| No | 4494 (89.8) | 41 (82.0) | 38 (90.5) |  |  |  |
| Yes | 513 (10.2) | 9 (18.0) | 4 (9.5) | **2.93 (1.37 to 6.27)** | 1.12 (0.39 to 3.20) | 0.39 (0.10 to 1.44) |
|  |  |  |  |  |  |  |
| Family history of breast cancer |  |  |  |  |  |  |
| No | 3948 (78.8) | 27 (54.0) | 28 (66.7) | 1.00 (Reference) | 1.00 (Reference) | 1.00 (Reference) |
| Yes | 916 (18.3) | 23 (46.0) | 14 (33.3) | **4.00 (2.27 to 7.05)** | **2.23 (1.17 to 4.26)** | 0.60 (0.25 to 1.43) |
| Missing | 143 (2.9) | 0 (0.0) | 0 (0.0) |  |  |  |
|  |  |  |  |  |  |  |
| Family history of ovarian cancer |  |  |  |  |  |  |
| No | 4753 (94.9) | 38 (76.0) | 36 (85.7) | 1.00 (Reference) | 1.00 (Reference) | 1.00 (Reference) |
| Yes | 231 (4.6) | 12 (24.0) | 6 (14.3) | **7.53 (3.82 to 14.82)** | **3.62 (1.50 to 8.71)** | 0.52 (0.17 to 1.61) |
| Missing | 23 (0.5) | 0 (0.0) | 0 (0.0) |  |  |  |
|  |  |  |  |  |  |  |
| Breast cancer in mother |  |  |  |  |  |  |
| No | 4392 (87.7) | 29 (58.0) | 32 (76.2) | 1.00 (Reference) | 1.00 (Reference) | 1.00 (Reference) |
| Yes | 579 (11.6) | 21 (42.0) | 10 (23.8) | 5.17 (2.92 to 9.17) | 2.29 (1.12 to 4.68) | 0.47 (0.18 to 1.20) |
| Missing | 36 (0.7) | 0 (0.0) | 0 (0.0) |  |  |  |
|  |  |  |  |  |  |  |
| Age at breast cancer diagnosis in mother |  |  |  |  |  |  |
| <50 | 92 (15.9) | 11 (52.4) | 4 (40.0) | 1.00 (Reference) | 1.00 (Reference) | 1.00 (Reference) |
| ≥59 | 446 (77.0) | 9 (42.9) | 6 (60.0) | 0.20 (0.08 to 0.50) | 0.37 (0.10 to 1.35) | 2.05 (0.39 to 10.67) |
| Missing | 43 (7.4) | 1 (4.8) | 0 (0.0) |  |  |  |
|  |  |  |  |  |  |  |
| Ovarian cancer in mother |  |  |  |  |  |  |
| No | 4822 (96.3) | 39 (78.0) | 36 (85.6) | 1.00 (Reference) | 1.00 (Reference) | 1.00 (Reference) |
| Yes | 149 (3.0) | 11 (22.0) | 6 (14.3) | 9.82 (4.85 to 19.89) | 5.44 (2.24 to 13.18) | 0.61 (0.20 to 1.86) |
| Missing | 36 (0.7) | 0 (0.0) | 0 (0.0) |  |  |  |
|  |  |  |  |  |  |  |
| Ovarian cancer in sister |  |  |  |  |  |  |
| No | 4885 (97.6) | 48 (96.0) | 42 (100.0) | 1.00 (Reference) | 1.00 (Reference) | 1.00 (Reference) |
| Yes | 86 (1.7) | 2 (4.0) | 0 (0.0) | 3.23 (0.76 to 13.76) | - | - |
| Missing | 36 (0.7) | 0 (0.0) | 0 (0.0) |  |  |  |

# **eTable 7.** Frequency, odds ratio (OR) and corresponding 95% confidence intervals (CI) of tumor characteristics according to *BRCA* status. *Adjusted for age (<50, 50-59, ≥60) and year of diagnosis (2001-2004 and 2005-2008).

| **Tumor characteristic** | **Non-*BRCA* (n=5,007)**  ***n* (%)** | ***BRCA1* (n=50)**  ***n* (%)** | ***BRCA2* (n=42)**  ***n* (%)** | ***BRCA1 vs***  ***non-BRCA OR (95% CI)**** | ***BRCA2 vs***  ***non-BRCA OR (95% CI)**** | ***BRCA2 vs  BRCA1 OR (95% CI)**** |
| --- | --- | --- | --- | --- | --- | --- |
| Type of breast cancer |  |  |  |  |  |  |
| Invasive | 4470 (89.3) | 48 (96.0) | 42 (100.0) | 1.00 (Reference) | 1.00 (Reference) | 1.00 (Reference) |
| Non-invasive | 522 (10.4) | 2 (4.0) | 0 (0.0) | 0.37 (0.09 to 1.53) | - | - |
| Missing | 15 (0.3) | 0 (0.0) | 0 (0.0) |  |  |  |
|  |  |  |  |  |  |  |
| Detection mode |  |  |  |  |  |  |
| Screen-detected | 1844 (36.8) | 5 (10.0) | 12 (28.6) | 1.00 (Reference) | 1.00 (Reference) | 1.00 (Reference) |
| Interval | 768 (15.3) | 5 (10.0) | 7 (16.7) | 2.36 (0.68 to 8.17) | 1.39 (0.54 to 3.54) | 0.63 (0.12 to 3.20 |
| Clinical cancer in women without  previous mammograms | 911 (18.2) | 8 (16.0) | 4 (9.5) | **3.99 (1.26 to 12.66)** | 0.76 (0.24 to 2.43) | 0.22 (0.04 to 1.08) |
| Clinical cancer in women who had  previous mammograms (i.e. interval >24 months) | 1395 (27.9) | 31 (62.0) | 19 (45.2) | **5.20 (1.78 to 15.15)** | 1.77 (0.73 to 4.29) | 0.35 (0.08 to 1.49) |
| Missing | 89 (1.8) | 1 (2.0) | 0 (0.0) |  |  |  |
|  |  |  |  |  |  |  |
| Estrogen receptor status |  |  |  |  |  |  |
| Positive | 3637 (72.6) | 17 (34.0) | 30 (71.4) | 1.00 (Reference) | 1.00 (Reference) | 1.00 (Reference) |
| Negative | 643 (12.8) | 30 (60.0) | 7 (16.7) | **8.98 (4.90 to 16.46)** | 1.23 (0.54 to 2.82) | **0.14 (0.05 to 0.39)** |
| Missing | 727 (14.5) | 3 (6.0) | 5 (11.9) |  |  |  |
|  |  |  |  |  |  |  |
| Progesterone receptor status |  |  |  |  |  |  |
| Positive | 2952 (59.0) | 14 (28.0) | 24 (57.1) | 1.00 (Reference) | 1.00 (Reference) | 1.00 (Reference) |
| Negative | 1252 (25.0) | 33 (66.0) | 13 (31.0) | **6.06 (3.21 to 11.46)** | 1.33 (0.67 to 2.63) | **0.23 (0.09 to 0.60)** |
| Missing | 803 (16.0) | 3 (6.0) | 5 (11.9) |  |  |  |
|  |  |  |  |  |  |  |
| Grade |  |  |  |  |  |  |
| Well-differentiated | 578 (11.5) | 1 (2.0) | 3 (7.1) | 1.00 (Reference) | 1.00 (Reference) | 1.00 (Reference) |
| Moderately differentiated | 1563 (31.2) | 7 (14.0) | 16 (38.1) | 2.41 (0.30 to 19.66) | 1.91 (0.55 to 6.60) | 0.80 (0.07 to 9.47) |
| Poorly differentiated | 822 (16.4) | 31 (62.0) | 9 (21.4) | **17.99 (2.44 to 132.70)** | 1.90 (0.51 to 7.10) | 0.11 (0.01 to 1.22) |
| Missing | 2044 (40.8) | 11 (22.0) | 14 (33.3) |  |  |  |
|  |  |  |  |  |  |  |
| Tumor size (mm) |  |  |  |  |  |  |
| <20 | 3020 (60.3) | 27 (54.0) | 23 (54.8) | 1.00 (Reference) | 1.00 (Reference) | 1.00 (Reference) |
| ≥20 | 1608 (32.1) | 21 (42.0) | 19 (45.2) | 1.30 (0.73 to 2.32) | 1.47 (0.80 to 2.72) | 1.16 (0.46 to 2.89) |
| Missing | 379 (7.6) | 2 (4.0) | 0 (0) |  |  |  |
|  |  |  |  |  |  |  |
| Nodal involvement |  |  |  |  |  |  |
| No | 4503 (89.9) | 39 (78.0) | 32 (76.2) | 1.00 (Reference) | 1.00 (Reference) | 1.00 (Reference) |
| Yes | 466 (9.3) | 11 (22.0) | 10 (23.8) | **2.08 (1.04 to 4.14)** | **2.71 (1.31 to 5.62)** | 1.27 (0.46 to 3.54) |
| Missing | 38 (0.8) | 0 (0.0) | 0 (0.0) |  |  |  |
|  |  |  |  |  |  |  |
| Proliferation level (Ki67) |  |  |  |  |  |  |
| Low (<20%) | 923 (18.4) | 5 (10.0) | 11 (26.2) | 1.00 (Reference) | 1.00 (Reference) | 1.00 (Reference) |
| High (≥20%) | 736 (14.7) | 20 (40.0) | 7 (16.7) | **4.25 (1.58 to 11.44)** | 0.72 (0.28 to 1.88) | **0.18 (0.04 to 0.74)** |
| Missing | 3348 (66.9) | 25 (50.0) | 24 (57.1) |  |  |  |
|  |  |  |  |  |  |  |
| Molecular subtypes |  |  |  |  |  |  |
| Luminal A | 1212 (24.2) | 5 (10.0) | 15 (35.7) | 1.00 (Reference) | 1.00 (Reference) | 1.00 (Reference) |
| Luminal B | 156 (3.1) | 2 (4.0) | 1 (2.4) | 2.83 ( 0.54 to 14.77) | 0.49 ( 0.06 to 3.73) | 0.19 (0.01 to 2.60) |
| HER2-enriched | 214 (4.3) | 1 (2.0) | 1 (2.4) | 0.93 ( 0.11 to 8.07) | 0.33 ( 0.04 to 2.52) | 0.38 (0.02 to 8.07) |
| Basal-like | 84 (1.7) | 17 (34.0) | 1 (2.4) | **40.07 (14.26 to 112.59)** | 0.84 ( 0.11 to 6.43) | **0.02 (0.00 to 0.17)** |
| Missing | 3341 (66.7) | 25 (50.0) | 24 (57.1) |  |  |  |

# **eTable 8.** Frequency, odds ratio (OR) and corresponding 95% confidence intervals (CI) of tumor characteristics among *BRCA* carriers identified versus not identified through selective clinical screening**.** * Adjusted for year of diagnosis (2001-2004, 2005-2008). † Adjusted for year of diagnosis and gene (*BRCA1*, *BRCA2*). ‡ Adjust for year of diagnosis, gene and age at diagnosis (<50, 50-59, ≥60).

| **Tumor characteristic** | **Not identified by  selective testing (*n*=57)**  ***n* (%)** | **Identified by  selective testing**  **(*n*=35)**  ***n* (%)** | **OR (95% CI)*** | **OR (95% CI)†** | **OR (95% CI)‡** |
| --- | --- | --- | --- | --- | --- |
|  |  |  |  |  |  |
| Type of breast cancer |  |  |  |  |  |
| Invasive | 56 (98.2) | 34 (97.1) | 1.00 (Reference) | 1.00 (Reference) | 1.00 (Reference) |
| Non-invasive | 1 (1.8) | 1 (2.9) | 2.27 (0.13 to 39.73) | 1.11 (0.06 to 20.11) | 1.44 (0.06 to 37.74) |
|  |  |  |  |  |  |
| Detection mode |  |  |  |  |  |
| Screen-detected | 14 (24.6) | 3 (8.6) | 1.00 (Reference) | 1.00 (Reference) | 1.00 (Reference) |
| Interval | 8 (14.0) | 4 (11.4) | 2.56 (0.44 to 14.85) | 2.24 (0.34 to 14.73) | 1.56 (0.21 to 11.33) |
| Clinical cancer in women without previous mammograms | 10 (17.5) | 2 (5.7) | 0.79 (0.11 to 5.72) | 0.41 (0.05 to 3.37) | 0.48 (0.06 to 4.06) |
| Clinical cancer in women who had previous mammograms  (i.e. interval >24 months) | 24 (42.1) | 26 (74.3) | **5.52 (1.38 to 22.18)** | 3.85 (0.88 to 16.87) | 1.88 (0.32 to 11.01) |
| Missing | 1 (1.8) | 0 (0.0) |  |  |  |
|  |  |  |  |  |  |
| Estrogen receptor |  |  |  |  |  |
| Positive | 34 (59.6) | 13 (37.1) | 1.00 (Reference) | 1.00 (Reference) | 1.00 (Reference) |
| Negative | 19 (33.3) | 18 (51.4) | 2.48 (0.99 to 6.19) | 1.29 (0.45 to 3.68) | 0.81 (0.25 to 2.63) |
| Missing | 4 (7.0) | 4 (11.4) |  |  |  |
|  |  |  |  |  |  |
| Progesterone receptor |  |  |  |  |  |
| Positive | 25 (43.9) | 13 (37.1) | 1.00 (Reference) | 1.00 (Reference) | 1.00 (Reference) |
| Negative | 27 (47.4) | 19 (54.3) | 1.30 (0.53 to 3.19) | 0.69 (0.24 to 1.97) | 0.46 (0.14 to 1.52) |
| Missing | 5 (8.8) | 3 (8.6) |  |  |  |
|  |  |  |  |  |  |
| Grade |  |  |  |  |  |
| Poorly-differentiated | 20 (35.1) | 20 (57.1) | 1.00 (Reference) | 1.00 (Reference) | 1.00 (Reference) |
| Intermediate-differentiated | 18 (31.6) | 5 (14.3) | **0.28 (0.08 to 0.92)** | 0.48 (0.13 to 1.78) | 0.67 (0.17 to 2.70) |
| Well-differentiated | 4 (7.0) | 0 (0.0) | - |  | - |
| Missing | 15 (26.3) | 10 (28.6) |  |  |  |
|  |  |  |  |  |  |
| Tumor size (mm) |  |  |  |  |  |
| <20 | 35 (61.4) | 15 (42.9) | 1.00 (Reference) | 1.00 (Reference) | 1.00 (Reference) |
| ≥20 | 16 (28.1) | 17 (48.6) | **2.48 (1.00 to 6.16)** | **2.91 (1.07 to 7.92)** | 2.15 (0.74 to 6.24) |
| Missing | 5 (8.8) | 2 (5.7) |  |  |  |
|  |  |  |  |  |  |
| Nodal involvement |  |  |  |  |  |
| No | 45 (78.9) | 26 (74.3) | 1.00 (Reference) | 1.00 (Reference) | 1.00 (Reference) |
| Yes | 12 (21.1) | 9 (25.7) | 1.40 (0.51 to 3.84) | 1.53 (0.52 to 4.52) | 1.15 (0.36 to 3.67) |
|  |  |  |  |  |  |
| Proliferation level (Ki67) |  |  |  |  |  |
| Low (<20%) | 11 (19.3) | 5 (14.3) | 1.00 (Reference) | 1.00 (Reference) | 1.00 (Reference) |
| High (≥20%) | 12 (21.1) | 15 (42.9) | 2.75 (0.75 to 10.11) | 1.55 (0.37 to 6.43) | 0.80 (0.16 to 3.96) |
| Missing | 34 (59.6) | 15 (42.9) |  |  |  |
|  |  |  |  |  |  |
| Molecular subtypes |  |  |  |  |  |
| Luminal A | 14 (24.6) | 6 (17.1) | 1.00 (Reference) | 1.00 (Reference) | 1.00 (Reference) |
| Luminal B | 2 (3.5) | 1 (2.9) | 1.17 (0.09 to 15.46) | 0.65 (0.04 to 9.93) | 0.37 (0.02 to 6.64) |
| HER2-enriched | 2 (3.5) | 0 (0.0) | - | - | - |
| Basal-like | 5 (8.8) | 13 (37.1) | **6.07 (1.49 to 24.76)** | 2.54 (0.52 to 12.41) | 1.49 (0.25 to 8.76) |
| Missing | 34 (59.6) | 15 (42.9) |  |  |  |

# **References**

1. Michailidou K, Hall P, Gonzalez-Neira A, Ghoussaini M, Dennis J, Milne RL, Schmidt MK, Chang-Claude J, Bojesen SE, Bolla MK, Wang Q, Dicks E, et al. Large-scale genotyping identifies 41 new loci associated with breast cancer risk. *Nat Genet* 2013;**45**: 353-61, 61e1-2.

2. Decker B, Allen J, Luccarini C, Pooley KA, Shah M, Bolla MK, Wang Q, Ahmed S, Baynes C, Conroy DM, Brown J, Luben R, et al. Rare, protein-truncating variants in ATM, CHEK2 and PALB2, but not XRCC2, are associated with increased breast cancer risks. *Journal of Medical Genetics* 2017: jmedgenet-2017-104588.

3. Li H. Exploring single-sample SNP and INDEL calling with whole-genome de novo assembly. *Bioinformatics* 2012;**28**: 1838-44.

4. Li H, Handsaker B, Wysoker A, Fennell T, Ruan J, Homer N, Marth G, Abecasis G, Durbin R, Genome Project Data Processing S. The Sequence Alignment/Map format and SAMtools. *Bioinformatics* 2009;**25**: 2078-9.

5. McKenna A, Hanna M, Banks E, Sivachenko A, Cibulskis K, Kernytsky A, Garimella K, Altshuler D, Gabriel S, Daly M, DePristo MA. The Genome Analysis Toolkit: a MapReduce framework for analyzing next-generation DNA sequencing data. *Genome Res* 2010;**20**: 1297-303.

6. Wang K, Li M, Hakonarson H. ANNOVAR: functional annotation of genetic variants from high-throughput sequencing data. *Nucleic Acids Res* 2010;**38**: e164.

7. Bar-Sade RB, Kruglikova A, Modan B, Gak E, Hirsh-Yechezkel G, Theodor L, Novikov I, Gershoni-Baruch R, Risel S, Papa MZ, Ben-Baruch G, Friedman E. The 185delAG BRCA1 mutation originated before the dispersion of Jews in the diaspora and is not limited to Ashkenazim. *Hum Mol Genet* 1998;**7**: 801-5.

8. Janavicius R. Founder BRCA1/2 mutations in the Europe: implications for hereditary breast-ovarian cancer prevention and control. *EPMA J* 2010;**1**: 397-412.

9. Johannsson O, Ostermeyer EA, Hakansson S, Friedman LS, Johansson U, Sellberg G, Brondum-Nielsen K, Sele V, Olsson H, King MC, Borg A. Founding BRCA1 mutations in hereditary breast and ovarian cancer in southern Sweden. *Am J Hum Genet* 1996;**58**: 441-50.

10. Loman N, Johannsson O, Kristoffersson U, Olsson H, Borg A. Family history of breast and ovarian cancers and BRCA1 and BRCA2 mutations in a population-based series of early-onset breast cancer. *J Natl Cancer Inst* 2001;**93**: 1215-23.

11. Bergman A, Einbeigi Z, Olofsson U, Taib Z, Wallgren A, Karlsson P, Wahlstrom J, Martinsson T, Nordling M. The western Swedish BRCA1 founder mutation 3171ins5; a 3.7 cM conserved haplotype of today is a reminiscence of a 1500-year-old mutation. *Eur J Hum Genet* 2001;**9**: 787-93.

12. Foretova L, Machackova E, Navratilova M, Pavlu H, Hruba M, Lukesova M, Valik D. BRCA1 and BRCA2 mutations in women with familial or early-onset breast/ovarian cancer in the Czech Republic. *Hum Mutat* 2004;**23**: 397-8.

13. Iyevleva AG, Suspitsin EN, Kroeze K, Gorodnova TV, Sokolenko AP, Buslov KG, Voskresenskiy DA, Togo AV, Kovalenko SP, Stoep N, Devilee P, Imyanitov EN. Non-founder BRCA1 mutations in Russian breast cancer patients. *Cancer Lett* 2010;**298**: 258-63.
