# Supplementary material for: Prevalence of BRCA1 and BRCA2 pathogenic variants in a large, unselected breast cancer cohort
Source: Int J Cancer. 2018 Nov 9;144(5):1195–204. doi: 10.1002/ijc.31841 (PMC6320715; doi:10.1002/ijc.31841)
Supplement: Supplementary file 2 — Data Supplement 2 [file IJC-144-1195-s002.docx]

**eTable 2.** Primer sequences and amplicon details. Partially overlapping amplicons were tiled across the target regions with a maximum amplicon length (forward and reverse primer length plus intervening unique sequence) of 200-bp. Primers were designed with melting temperature range 59.0-61.0 ̊C. Each primer included an orientation-specific tail sequence for subsequent ligation of adapter and barcode sequences.

| **Gene/Chr** | **Assay Name** | **Chr** | **Start** | **End** | **Forward primer** | **Reverse primer** | **Total amplicon length** | **GC %** | **Amplicon** | **Flag** |
| --- | --- | --- | --- | --- | --- | --- | --- | --- | --- | --- |
| BRCA1 | BRCA1_3_UTR__exon_24__1 | 17 | 41196256 | 41196454 | ACACTGACGACATGGTTCTACAgtgaAAAGGCTCTGAGAAAGTCG | TACGGTAGCAGAGACTTGGTCTGGACTGTTTATAGCTGTTGGAAGGA | 199 | 45 | gtgaAAAGGCTCTGAGAAAGTCGGCTGGCCTAAGTCTCAAGAACAGTCATTCATGGTGGAAGTGTTTGCTACCAAGTTTATTTGCAGTGTTAACAGCACAACATTTACAAAACGTATTTTGTACAATCAAGTCTTCACTGCCCTTGCACACTGGGGGGGCTAGGGAAGACCTAGTCCTTCCAACAGCTATAAACAGTCC | One primer sits in the repeat region |
| BRCA1 | BRCA1_3_UTR__exon_24__2 | 17 | 41196303 | 41196500 | ACACTGACGACATGGTTCTACACATTCATGGTGGAAGTGTTTGCT | TACGGTAGCAGAGACTTGGTCTTGCTTGCTGAAGGAAGAAAAAGTG | 198 | 41 | CATTCATGGTGGAAGTGTTTGCTACCAAGTTTATTTGCAGTGTTAACAGCACAACATTTACAAAACGTATTTTGTACAATCAAGTCTTCACTGCCCTTGCACACTGGGGGGGCTAGGGAAGACCTAGTCCTTCCAACAGCTATAAACAGTCCTGGATAATGGGTTTATGAAAAACACTTTTTCTTCCTTCAGCAAGCA |  |
| BRCA1 | BRCA1_3UTRB_3 | 17 | 41196422 | 41196600 | ACACTGACGACATGGTTCTACAAGACCTAGTCCTTCCAACAGCTA | TACGGTAGCAGAGACTTGGTCTAAATGACAGATCCCACCAGGAAG | 179 | 39 | AGACCTAGTCCTTCCAACAGCTATAAACAGTCCTGGATAATGGGTTTATGAAAAACACTTTTTCTTCCTTCAGCAAGCAAAATTATTTATGAAGCTGTATGGTTTCAGCAACAGGGAGCAAAGGAAAAAAATCACCTCAAAGAAAGCAACAGCTTCCTTCCTGGTGGGATCTGTCATTT |  |
| BRCA1 | BRCA1_3UTRB_4 | 17 | 41196516 | 41196686 | ACACTGACGACATGGTTCTACACTGTATGGTTTCAGCAACAGGGA | TACGGTAGCAGAGACTTGGTCTAGTAAAATGTTTATTGTTGTAGCTCTGG | 171 | 36 | CTGTATGGTTTCAGCAACAGGGAGCAAAGGAAAAAAATCACCTCAAAGAAAGCAACAGCTTCCTTCCTGGTGGGATCTGTCATTTTATAGATATGAAATATTCATGCCAGAGGTCTTATATTTTAAGAGGAATGGATTATATACCAGAGCTACAACAATAAACATTTTACT |  |
| BRCA1 | BRCA1_3_UTR__exon_24__5 | 17 | 41196609 | 41196779 | ACACTGACGACATGGTTCTACATGAAATATTCATGCCAGAGGTCTT | TACGGTAGCAGAGACTTGGTCTTTTGCTAGATTTCTAAAGAATGTGTTCTAA | 171 | 31 | TGAAATATTCATGCCAGAGGTCTTATATTTTAAGAGGAATGGATTATATACCAGAGCTACAACAATAAACATTTTACTTATTACTAATGAGGAATTAGAAGACTGTCTTTGGAAACCGGTTCTTGAAAATCTTCTGCTGTTTTAGAACACATTCTTTAGAAATCTAGCAAA |  |
| BRCA1 | BRCA1_3_UTR__exon_24__6 | 17 | 41196796 | 41196982 | ACACTGACGACATGGTTCTACAAGAAATCTCTTCTAGTTTCATTTTCCTTT | TACGGTAGCAGAGACTTGGTCTCGGTCATGGTGGTGGACA | 187 | 49 | AGAAATCTCTTCTAGtttcattttcctttttttttttttttttttgagccacagtctcactgtcacccaggctggagtgccgtggtatgatcttggctcactgcaacctccacctcccgggctgaagtgattctcctgccttagccacctgagtagctgggattacaggtgtccaccaccatgaccg | Two primers sits in the repeat region |
| BRCA1 | BRCA1_3_UTR__exon_24__7 | 17 | 41196962 | 41197133 | ACACTGACGACATGGTTCTACAAGGTGTCCACCACCATGA | TACGGTAGCAGAGACTTGGTCTAAGGAAACTTGAAACCTGGGCAT | 172 | 52 | aggtgtccaccaccatgaccggctaatttctgtatttttagtagagatggggtttcaccatgttggccaggctggtttcgaactcctgacctccagtgatctgcccaccttggcctcccaaagtgctgggattacaggcgtgagccaccatgcccagGTTTCAAGTTTCCTT | Two primers sits in the repeat region |
| BRCA1 | BRCA1_3_UTR__exon_24__8 | 17 | 41197109 | 41197273 | ACACTGACGACATGGTTCTACAccatgcccagGTTTCAAGTTT | TACGGTAGCAGAGACTTGGTCTGCAGAGAGTCAGACCCTTCAAT | 165 | 45 | ccatgcccagGTTTCAAGTTTCCTTTTCATTTCTAATACCTGCCTCAGAATTTCCTCCCCAATGTTCCACTCCAACATTTGAGAACTGCCCAAGGACTATTCTGACTTTAAGTCACATAATCGATCCCAAGCACTCTCCTTCCATTGAAGGGTCTGACTCTCTGC | One primer sits in the repeat region |
| BRCA1 | BRCA1_3_UTR__exon_24__9 | 17 | 41197192 | 41197381 | ACACTGACGACATGGTTCTACAAACTGCCCAAGGACTATTCTGAC | TACGGTAGCAGAGACTTGGTCTTCTATTCAGGCTGTTGTTGGCTT | 190 | 46 | AACTGCCCAAGGACTATTCTGACTTTAAGTCACATAATCGATCCCAAGCACTCTCCTTCCATTGAAGGGTCTGACTCTCTGCCTTTGTGAACACAGGGTTTTAGAGAAGTAAACTTAGGGAAACCAGCTATTCTCTTGAGGCCAAGCCACTCTGTGCTTCCAGCCCTAAGCCAACAACAGCCTGAATAGA |  |
| BRCA1 | BRCA1_3_UTR__exon_24__10 | 17 | 41197309 | 41197497 | ACACTGACGACATGGTTCTACAGGGAAACCAGCTATTCTCTTGAGG | TACGGTAGCAGAGACTTGGTCTGGAAATCTGCCATGAGCACAAAA | 189 | 41 | GGGAAACCAGCTATTCTCTTGAGGCCAAGCCACTCTGTGCTTCCAGCCCTAAGCCAACAACAGCCTGAATAGAAAGAATAGGGCTGATAAATAATGAATCAGCATCTTGCTCAATTGGTGGCGTTTAAATGGTTTTAAAATCTTCTCAGGTGAAAAATTACCATAATTTTGTGCTCATGGCAGATTTCC |  |
| BRCA1 | BRCA1_3_UTR__exon_24__11 | 17 | 41197360 | 41197558 | ACACTGACGACATGGTTCTACAAGCCAACAACAGCCTGAATAGAA | TACGGTAGCAGAGACTTGGTCTCTGAAAAGGACTTCTGGCTATGC | 199 | 39 | AGCCAACAACAGCCTGAATAGAAAGAATAGGGCTGATAAATAATGAATCAGCATCTTGCTCAATTGGTGGCGTTTAAATGGTTTTAAAATCTTCTCAGGTGAAAAATTACCATAATTTTGTGCTCATGGCAGATTTCCAAGGGAGACTTCAAGCAGAAAATCTTTAAGGGACCCTTGCATAGCCAGAAGTCCTTTTCAG |  |
| BRCA1 | BRCA1_3_UTR__exon_24__12 | 17 | 41197488 | 41197682 | ACACTGACGACATGGTTCTACAGCAGATTTCCAAGGGAGACTTCA | TACGGTAGCAGAGACTTGGTCTACAGGTACAGAGCCACAGGA | 195 | 48 | GCAGATTTCCAAGGGAGACTTCAAGCAGAAAATCTTTAAGGGACCCTTGCATAGCCAGAAGTCCTTTTCAGGCTGATGTACATAAAATATTTAGTAGCCAGGACAGTAGAAGGACTGAAGAGTGAGAGGAGCTCCCAGGGCCTGGAAAGGCCACTTTGTAAGCTCATTCTTGGGGTCCTGTGGCTCTGTACCTGT |  |
| BRCA1 | BRCA1_3UTRA_3 | 17 | 41197582 | 41197779 | ACACTGACGACATGGTTCTACATAGCCAGGACAGTAGAAGGACTG | TACGGTAGCAGAGACTTGGTCTGTGGGTGTTGGACAGTGTAGC | 198 | 57 | TAGCCAGGACAGTAGAAGGACTGAAGAGTGAGAGGAGCTCCCAGGGCCTGGAAAGGCCACTTTGTAAGCTCATTCTTGGGGTCCTGTGGCTCTGTACCTGTGGCTGGCTGCAGTCAGTAGTGGCTGTGGGGGATCTGGGGTATCAGGTAGGTGTCCAGCTCCTGGCACTGGTAGAGTGCTACACTGTCCAACACCCAC | Assays designed by relax mode and have no off-target hits |
| BRCA1 | BRCA1_1 | 17 | 41197714 | 41197895 | ACACTGACGACATGGTTCTACAATCTGGGGTATCAGGTAGGTGTC | TACGGTAGCAGAGACTTGGTCTCCTGGAGTCGATTGATTAGAGCC | 182 | 52 | ATCTGGGGTATCAGGTAGGTGTCCAGCTCCTGGCACTGGTAGAGTGCTACACTGTCCAACACCCACTCTCGGGTCACCACAGGTGCCTCACACATCTGCCCAATTGCTGGAGACAGAGAACACAAGCAGAGATTAGTGTCAATTCATTCTCCTGGACTAGGCTCTAATCAATCGACTCCAGG | Assays designed by relax mode and have no off-target hits |
| BRCA1 | BRCA1_2 | 17 | 41199563 | 41199750 | ACACTGACGACATGGTTCTACATGCCAAGAACTGTGCTACTCAAG | TACGGTAGCAGAGACTTGGTCTTTGAATGCTCTTTCCTTCCTGGG | 188 | 51 | tgccaagaactgtgctactcaagcaccaggtaatgagtgataaaccaaacccatgcaaaagGACCCCATATAGCACAGGTACATGCAGGCACCTTACCATGGAAGCCATTGTCCTCTGTCCAGGCATCTGGCTGCACAACCACAATTGGGTGGACACCCTGGATCCCCAGGAAGGAAAGAGCATTCAA | One primer sits in the repeat region |
| BRCA1 | BRCA1_3 | 17 | 41201071 | 41201269 | ACACTGACGACATGGTTCTACAGGTGCCAGTCTTGCTCACAG | TACGGTAGCAGAGACTTGGTCTGGCCTGGGTTAAGTATGCAGAT | 199 | 47 | GGTGCCAGTCTTGCTCACAGGAGAGAATATTGTGTCCTCCCTCTCTGACAGGGCACCCAATACTTACTGTGCCAAGGGTGAATGATGAAAGCTCCTTCACCACAGAAGCACCACACAGCTGTACCATCCATTCCAGTTGATCTAAAATGGACATTTAGATGTAAAATCACTGCAGTAATCTGCATACTTAACCCAGGCC |  |
| BRCA1 | BRCA1_4 | 17 | 41202996 | 41203184 | ACACTGACGACATGGTTCTACACCCATCGTGGGATCTTGCTTAT | TACGGTAGCAGAGACTTGGTCTTTTTCCTTCTCTCCATTCCCCTG | 189 | 51 | CCCATCGTGGGATCTTGCTTATAATACTCCACTATGTAAGACAAAGGCTGGTGCTGGAACTCTGGGGTTCTCCCAGGCTCTTACCTGTGGGCATGTTGGTGAAGGGCCCATAGCAACAGATTTCTAGCCCCCTGAAGATCTGGAAGAAGAGAGGAAGAGAGAGGGACAGGGGAATGGAGAGAAGGAAAA |  |
| BRCA1 | BRCA1_5 | 17 | 41208999 | 41209195 | ACACTGACGACATGGTTCTACACTCTGCAAAGGGGAGTGGAATAC | TACGGTAGCAGAGACTTGGTCTTCTCTTTCTCTTATCCTGATGGGT | 197 | 46 | CTCTGCAAAGGGGAGTGGAATACAGAGTGGTGGGGTGAGATTTTTGTCAACTTGAGGGAGGGAGCTTTACCTTTCTGTCCTGGGATTCTCTTGCTCGCTTTGGACCTTGGTGGTTTCTTCCATTGACCACATCTCCTCTGACTTCAAAATCATGCTGAAAGAAACCAAACACAACCCATCAGGATAAGAGAAAGAGA |  |
| BRCA1 | BRCA1_6 | 17 | 41215258 | 41215442 | ACACTGACGACATGGTTCTACAGAAAGTGGTGCATTGATGGAAGG | TACGGTAGCAGAGACTTGGTCTAAGAGCACGTTCTTCTGCTGTAT | 185 | 36 | GAAAGTGGTGCATTGATGGAAGGAAGCAAATACATTTTTAACTATATGACTGAATGAATATCTCTGGTTAGTTTGTAACATCAAGTACTTACCTCATTCAGCATTTTTCTTTCTTTAATAGACTGGGTCACCCCTAAAGAGATCATAGAAAAGACAGGTTACATACAGCAGAAGAACGTGCTCTT |  |
| BRCA1 | BRCA1_7 | 17 | 41215830 | 41216019 | ACACTGACGACATGGTTCTACAGGTGTAAAAATGCAATTCTGAGGTGT | TACGGTAGCAGAGACTTGGTCTTCCTGATTTTGTTTTCAACTTCTAATCC | 190 | 37 | GGTGTAAAAATGCAATTCTGAGGTGTTAAAGGGAGGAGGGGAGAAATAGTATTATACTTACAGAAATAGCTAACTACCCATTTTCCTCCCGCAATTCCTAGAAAATATTTCAGTGTCCGTTCACACACAAACTCAGCATCTGCAGAATGAAAAACACTCAAAGGATTAGAAGTTGAAAACAAAATCAGGA |  |
| BRCA1 | BRCA1_8 | 17 | 41219582 | 41219777 | ACACTGACGACATGGTTCTACATATGCAGCAGATGCAAGGTATTC | TACGGTAGCAGAGACTTGGTCTAACTAGTATTCTGAGCTGTGTGC | 196 | 35 | TATGCAGCAGATGCAAGGTATTCTGTAAAGGTTCTTGGTATACCTGTTTTCATAACAACATGAGTAGTCTCTTCAGTAATTAGATTAGTTAAAGTGATGTGGTGTTTTCTGGCAAACTTGTACACGAGCATCTGAAATTAAATCAAATATTCCATTATCATGAGTTACCTCTAGCACACAGCTCAGAATACTAGTT |  |
| BRCA1 | BRCA1_Intron_16_1 | 17 | 41220845 | 41221039 | ACACTGACGACATGGTTCTACACGTAAGAAGGCTTAGAAGGGGTTC | TACGGTAGCAGAGACTTGGTCTTGGCTAGTAAATGATGGCCCTTG | 195 | 37 | CGTAAGAAGGCTTAGAAGGGGTTCATGTTCTTCCCCAAAGAAATTTAGAGTCCTCTAGCTATTATCTATCAACCAAATAAAAATCAATAGGCAGCAAAAGAAATTAACCAGATCAATTATGGGCACAGTTGACCTAGCTGGTAGGTATTGTCTGATAAATAACTTTGTTTACCAAGGGCCATCATTTACTAGCCA |  |
| BRCA1 | BRCA1_Intron_16_2 | 17 | 41220960 | 41221131 | ACACTGACGACATGGTTCTACAATTATGGGCACAGTTGACCTAGC | TACGGTAGCAGAGACTTGGTCTAGTATGGAGTGAGGCAACATGAA | 172 | 44 | ATTATGGGCACAGTTGACCTAGCTGGTAGGTATTGTCTGATAAATAACTTTGTTTACCAAGGGCCATCATTTACTAGCCATAGCCACAGCCACTCACACCAGTATTTACGTGTTGCACAATACTGCCATGAAGGTCAGGCCATCTTTCTTTCATGTTGCCTCACTCCATACT |  |
| BRCA1 | BRCA1_Intron_16_3 | 17 | 41221006 | 41221202 | ACACTGACGACATGGTTCTACAACTTTGTTTACCAAGGGCCATCA | TACGGTAGCAGAGACTTGGTCTCGGAGGGTGGTAGCAAAAGG | 197 | 49 | ACTTTGTTTACCAAGGGCCATCATTTACTAGCCATAGCCACAGCCACTCACACCAGTATTTACGTGTTGCACAATACTGCCATGAAGGTCAGGCCATCTTTCTTTCATGTTGCCTCACTCCATACTGCCACAGGGCACTGAAAAAAGGCAGGCTAGAGAAGCTAATGACATCCTCCCCCTTTTGCTACCACCCTCCG |  |
| BRCA1 | BRCA1_Intron_16_4 | 17 | 41221097 | 41221291 | ACACTGACGACATGGTTCTACAGGCCATCTTTCTTTCATGTTGCC | TACGGTAGCAGAGACTTGGTCTTTCAGCAGTTCAGCATTGCTCTT | 195 | 47 | GGCCATCTTTCTTTCATGTTGCCTCACTCCATACTGCCACAGGGCACTGAAAAAAGGCAGGCTAGAGAAGCTAATGACATCCTCCCCCTTTTGCTACCACCCTCCGCCAGGATCATTAGCAATTCTGAACTGATTAATTGCTGAAATCCCAGCCTTGAAACTGTTCAGTTAGAAGAGCAATGCTGAACTGCTGAA |  |
| BRCA1 | BRCA1_9 | 17 | 41222884 | 41223082 | ACACTGACGACATGGTTCTACAAACTCTTTCCAGAATGTTGTTAAGTC | TACGGTAGCAGAGACTTGGTCTGCTCATACTACTGATACTGCTGGG | 199 | 43 | AACTCTTTCCAGAATGTTGTTAAGTCTTAGTCATTAGGGAGATACATATGGATACACTCACAAATTCTTCTGGGGTCAGGCCAGACACCACCATGGACATTCTTTTGTTGACCCTTTCTGTTGAAGCTGTCAATTCTGGCTTCTCCCTGCTCACACTTTCTTCCATTGCATTATACCCAGCAGTATCAGTAGTATGAGC | Assays designed by relax mode and have no off-target hits |
| BRCA1 | BRCA1_10 | 17 | 41222991 | 41223181 | ACACTGACGACATGGTTCTACATTGACCCTTTCTGTTGAAGCTGT | TACGGTAGCAGAGACTTGGTCTGCCCCAGAGTCAGCTCGT | 191 | 48 | TTGACCCTTTCTGTTGAAGCTGTCAATTCTGGCTTCTCCCTGCTCACACTTTCTTCCATTGCATTATACCCAGCAGTATCAGTAGTATGAGCAGCAGCTGGACTCTGGGCAGATTCTGCAACTTTCAATTGGGGAACTTTCAATGCAGAGGTTGAAGATGGTATGTTGCCAACACGAGCTGACTCTGGGGC | Assays designed by relax mode and have no off-target hits |
| BRCA1 | BRCA1_11 | 17 | 41223105 | 41223287 | ACACTGACGACATGGTTCTACATCTGCAACTTTCAATTGGGGAAC | TACGGTAGCAGAGACTTGGTCTTGTGTAAATTAAACTTCTCCCATTCCT | 183 | 46 | TCTGCAACTTTCAATTGGGGAACTTTCAATGCAGAGGTTGAAGATGGTATGTTGCCAACACGAGCTGACTCTGGGGCTCTGTCTTCAGAAGGATCAGATTCAGGGTCATCAGAGAAGAGGCTGATTCCAGATTCCAGGTAAGGGGTTCCCTCTGAAAGGAATGGGAGAAGTTTAATTTACACA | Assays designed by relax mode and have no off-target hits |
| BRCA1 | BRCA1_12 | 17 | 41226290 | 41226486 | ACACTGACGACATGGTTCTACAATGTAGGATTCAGAGTAAAATCAAAGTG | TACGGTAGCAGAGACTTGGTCTTGCTCTGGGAGTCTTCAGAATAGA | 197 | 43 | ATGTAGGATTCAGAGTAAAATCAAAGTGTTTGTTCCAATACAGCAGATGAAATATTACCTAGATCTTGCCTTGGCAAGTAAGATGTTTCCGTCAAATCGTGTGGCCCAGACTCTTCCAGCTGTTGCTCCTCCACATCAACAACCTTAATGAGCTCCTCTTGAGATGGGTAGTTTCTATTCTGAAGACTCCCAGAGCA | Assays designed by relax mode and have no off-target hits |
| BRCA1 | BRCA1_13 | 17 | 41226396 | 41226580 | ACACTGACGACATGGTTCTACACAGACTCTTCCAGCTGTTGCT | TACGGTAGCAGAGACTTGGTCTCAATTGGTGGCGATGGTTTTCTC | 185 | 45 | CAGACTCTTCCAGCTGTTGCTCCTCCACATCAACAACCTTAATGAGCTCCTCTTGAGATGGGTAGTTTCTATTCTGAAGACTCCCAGAGCAACTGTGCATGTACCACCTATCATCTAATGATGGGCATTTAGAAGGGGATGACCTAGAAAGATAAATGGAAGGAGAAAACCATCGCCACCAATTG | Assays designed by relax mode and have no off-target hits |
| BRCA1 | BRCA1_14 | 17 | 41228395 | 41228576 | ACACTGACGACATGGTTCTACATGCCTGTATGCAAAAAACTGGAG | TACGGTAGCAGAGACTTGGTCTCCTTTCTGCTGACAAGTTTGAGG | 182 | 35 | TGCCTGTATGCAAAAAACTGGAGAAAGTATGGTGAAAAAAATTAACAATCAGAGTTCAATATAAATAAAGATGTCAGATACCACAGCATCTTTACATTGATGTTTCTTACCTTTCCACTCCTGGTTCTTTATTTTTACTGGTAGAACTATCTGCAGACACCTCAAACTTGTCAGCAGAAAGG |  |
| BRCA1 | BRCA1_15 | 17 | 41228496 | 41228684 | ACACTGACGACATGGTTCTACAGTTTCTTACCTTTCCACTCCTGGT | TACGGTAGCAGAGACTTGGTCTTCACTATCAGAACAAAGCAGTAAAGT | 189 | 37 | GTTTCTTACCTTTCCACTCCTGGTTCTTTATTTTTACTGGTAGAACTATCTGCAGACACCTCAAACTTGTCAGCAGAAAGGCCTTCTGGATTCTGGCTTATAGGGTATTCACTACTTTTCTGTGAAGTTAATACTGCTTTAAATGGAATGAGAAAACAAATCTACTTTACTGCTTTGTTCTGATAGTGA | Assays designed by relax mode and have no off-target hits |
| BRCA1 | BRCA1_16 | 17 | 41231286 | 41231459 | ACACTGACGACATGGTTCTACAGCCAGAACCACCATCTTTCAGTA | TACGGTAGCAGAGACTTGGTCTAGTACCCGTTCCCTTGATGTCTA | 174 | 42 | GCCAGAACCACCATCTTTCAGTAATTTGCCAAAATGACGAACACAAAGGGAAAGAGGAGAGGCACCTGATATATGTTCTCTAGGCCTTTTAGAAAACATGGAGTTGTTCCTTTGGCCATGTATATGCGAATCTGTAAGAAAGGTGAAATTGTAGACATCAAGGGAACGGGTACT |  |
| BRCA1 | BRCA1_Intron_13_1 | 17 | 41231398 | 41231595 | ACACTGACGACATGGTTCTACATGGCCATGTATATGCGAATCTGT | TACGGTAGCAGAGACTTGGTCTTCAATACGCACATTAATTCTCTTGGC | 198 | 43 | TGGCCATGTATATGCGAATCTGTAAGAAAGGTGAAATTGTAGACATCAAGGGAACGGGTACTGTTCAAAAAGGAATGCCCCACAAGTGTTACCATGGCAAAACTGGAAAAGTCTACAACGTGACCCAGCATGCTGTTGGCATCGCTGTAAACAAGTTAAGGGCAAGATTCTTGCCAAGAGAATTAATGTGCGTATTGA |  |
| BRCA1 | BRCA1_Intron_13_2 | 17 | 41231492 | 41231689 | ACACTGACGACATGGTTCTACATGGCAAAACTGGAAAAGTCTACA | TACGGTAGCAGAGACTTGGTCTCCACGTACCTTTCTCTTTGGCTT | 198 | 43 | TGGCAAAACTGGAAAAGTCTACAACGTGACCCAGCATGCTGTTGGCATCGCTGTAAACAAGTTAAGGGCAAGATTCTTGCCAAGAGAATTAATGTGCGTATTGAGCACATTAAACACTCTAAGAGCCAAGACAGCTTCCTGAAACGCGTGAAGGAAAATGATCAGAAAAAGAAAGAAGCCAAAGAGAAAGGTACGTGG |  |
| BRCA1 | BRCA1_Intron_13_3 | 17 | 41231596 | 41231778 | ACACTGACGACATGGTTCTACAGCACATTAAACACTCTAAGAGCCAA | TACGGTAGCAGAGACTTGGTCTGGAATAGGTTCCAGCTGCTCA | 183 | 48 | GCACATTAAACACTCTAAGAGCCAAGACAGCTTCCTGAAACGCGTGAAGGAAAATGATCAGAAAAAGAAAGAAGCCAAAGAGAAAGGTACGTGGGTTCAACTGAAGCACCAGCCTGCTCCACCCAGAGAAGCACACTTTGTGAGAACCAATGGGAAGGAGCCTGAGCAGCTGGAACCTATTCC |  |
| BRCA1 | BRCA1_Intron_13_4 | 17 | 41231671 | 41231869 | ACACTGACGACATGGTTCTACACAAAGAGAAAGGTACGTGGGTTC | TACGGTAGCAGAGACTTGGTCTTGTTCTGGTGATAGAATGACTTTTCTT | 199 | 41 | CAAAGAGAAAGGTACGTGGGTTCAACTGAAGCACCAGCCTGCTCCACCCAGAGAAGCACACTTTGTGAGAACCAATGGGAAGGAGCCTGAGCAGCTGGAACCTATTCCCTATGAATTCATGGCATAATAGGTGTTAAAAAAAAAAAAAAAAGACCTTTGGACTGTAAAAAAAAAGAAAAGTCATTCTATCACCAGAACA |  |
| BRCA1 | BRCA1_Intron_13_5 | 17 | 41231760 | 41231936 | ACACTGACGACATGGTTCTACAAGCAGCTGGAACCTATTCCCTAT | TACGGTAGCAGAGACTTGGTCTACAAGTTATCTTATTTGCTTCATTGCAT | 177 | 32 | AGCAGCTGGAACCTATTCCCTATGAATTCATGGCATAATAGGTGTTAAAAAAAAAAAAAAAAGACCTTTGGACTGTAAAAAAAAAGAAAAGTCATTCTATCACCAGAACATTTAGCATATAAATTCCTCTTCTTACTACAATGGGCCTCATGCAATGAAGCAAATAAGATAACTTGT |  |
| BRCA1 | BRCA1_Intron_13_6 | 17 | 41231851 | 41232048 | ACACTGACGACATGGTTCTACATCATTCTATCACCAGAACATTTAGCA | TACGGTAGCAGAGACTTGGTCTCACAAGGCTTAGGTTGGGCTT | 198 | 35 | TCATTCTATCACCAGAACATTTAGCATATAAATTCCTCTTCTTACTACAATGGGCCTCATGCAATGAAGCAAATAAGATAACTTGTTAGAAGTTAACAACTCAATAGAACCTGAAAAACAGAGCAAAACCTTTCTGCTTTTTTTTCTCCTTAATCCTTAATTCATTCTCTGAACAGCAAGCCCAACCTAAGCCTTGTG |  |
| BRCA1 | BRCA1_Intron_13_7 | 17 | 41231905 | 41232081 | ACACTGACGACATGGTTCTACACCTCATGCAATGAAGCAAATAAGAT | TACGGTAGCAGAGACTTGGTCTTTCCTAAACCAGCCAGCAAGTAG | 177 | 37 | CCTCATGCAATGAAGCAAATAAGATAACTTGTTAGAAGTTAACAACTCAATAGAACCTGAAAAACAGAGCAAAACCTTTCTGCTTTTTTTTCTCCTTAATCCTTAATTCATTCTCTGAACAGCAAGCCCAACCTAAGCCTTGTGACATGATGATCTACTTGCTGGCTGGTTTAGGAA |  |
| BRCA1 | BRCA1_Intron_13_8 | 17 | 41232004 | 41232201 | ACACTGACGACATGGTTCTACATCCTTAATTCATTCTCTGAACAGCA | TACGGTAGCAGAGACTTGGTCTTGGCCCATACTTCCTATCCACAA | 198 | 39 | TCCTTAATTCATTCTCTGAACAGCAAGCCCAACCTAAGCCTTGTGACATGATGATCTACTTGCTGGCTGGTTTAGGAAAGTCTAAATGCAGAGATCCAGAGTCCAAGTTTCAGAATATTATTGTAGTTCCTCTTAAATCATTCCCTCATCTAGATGTCTTAACATGTTACATTCTTTGTGGATAGGAAGTATGGGCCA |  |
| BRCA1 | BRCA1_17 | 17 | 41234313 | 41234511 | ACACTGACGACATGGTTCTACAGAGCAAGGATCATAAAATGTTGGAG | TACGGTAGCAGAGACTTGGTCTAGCCAGCCTTCTAACAGCTAC | 199 | 43 | GAGCAAGGATCATAAAATGTTGGAGCTAGGTCCTTACTCTTCAGAAGGAGATAAAGGGGAAGGAAAGAATTTTGCTTAAGATATCAGTGTTTGGCCAACAATACACACCTTTTTCTGATGTGCTTTGTTCTGGATTTCGCAGGTCCTCAAGGGCAGAAGAGTCACTTATGATGGAAGGGTAGCTGTTAGAAGGCTGGCT |  |
| BRCA1 | BRCA1_18 | 17 | 41234442 | 41234625 | ACACTGACGACATGGTTCTACACTGGATTTCGCAGGTCCTCAAG | TACGGTAGCAGAGACTTGGTCTTCATTTTCTTGGTGCCATTTATCGT | 184 | 46 | CTGGATTTCGCAGGTCCTCAAGGGCAGAAGAGTCACTTATGATGGAAGGGTAGCTGTTAGAAGGCTGGCTCCCATGCTGTTCTAACACAGCTTCTAGTTCAGCCATTTCCTGCTGGAGCTTTATCAGGTTATGTTGCATGGTATCCCTCTGCTTCAAAAACGATAAATGGCACCAAGAAAATGA | Assays designed by relax mode and have no off-target hits |
| BRCA1 | BRCA1_Intron_12__region_2__1 | 17 | 41236576 | 41236770 | ACACTGACGACATGGTTCTACAACTCTCCGTTTCTTCTTCTGATCC | TACGGTAGCAGAGACTTGGTCTAATGGCATTGCTGCAGACCTT | 195 | 40 | ACTCTCCGTTTCTTCTTCTGATCCTTAAAAATGACTTTGCCCTTTACTAAAAACAAGCCATCTTCTTTCTCCTGCTACACTGAGTTCACAATTTTCAGGAGGTTTTTATGACCAGAAAATGTATTCCTAGCTTCACAAAGGTTTCTACTGCTACTCTAACACTGCTTTGCTGCCAAGGTCTGCAGCAATGCCATT |  |
| BRCA1 | BRCA1_Intron_12__region_2__2 | 17 | 41236708 | 41236884 | ACACTGACGACATGGTTCTACATCACAAAGGTTTCTACTGCTACTCT | TACGGTAGCAGAGACTTGGTCTCCTGTGAAGCATTTTGGTCAGAT | 177 | 46 | TCACAAAGGTTTCTACTGCTACTCTAACACTGCTTTGCTGCCAAGGTCTGCAGCAATGCCATTGCCACCACGTGGTACTCGTGAGTAGTTACATCACTGATCAAACCCTAGCCAAAGTCTTGCCTCAGTCAACCTGGAATGAAAGCACATAGATATCTGACCAAAATGCTTCACAGG |  |
| BRCA1 | BRCA1_Intron_12__region_2__3 | 17 | 41236803 | 41236999 | ACACTGACGACATGGTTCTACAACTGATCAAACCCTAGCCAAAGT | TACGGTAGCAGAGACTTGGTCTTTGGGAAACCCAAAGGAAAGTTA | 197 | 40 | ACTGATCAAACCCTAGCCAAAGTCTTGCCTCAGTCAACCTGGAATGAAAGCACATAGATATCTGACCAAAATGCTTCACAGGCTGTATTAGTCACTACTGTCACAACCATCACATCTAACCTCTACGTGGCCATTACATAAATCAGAAAAAGTAGTGGGGTAATTATTAATAGATAACTTTCCTTTGGGTTTCCCAA |  |
| BRCA1 | BRCA1_Intron_12__region_1__1 | 17 | 41237464 | 41237643 | ACACTGACGACATGGTTCTACATGCCATTAGGATAAAATTCCTCAACA | TACGGTAGCAGAGACTTGGTCTAAAAGAGGAGTGGCAGCAAGTAA | 180 | 41 | TGCCATTAGGATAAAATTCCTCAACATAGCATTCAAAAGGCCCTTTATACCCTGGTATTATCTCTCATTTCTCCCAACACCCCTCACACAAGTTGTTTTCCTATAACACATGACAACTTTACGTTTCCTGGGAATTCCATGACTCTGTACCTTTACCTTACTTGCTGCCACTCCTCTTTT |  |
| BRCA1 | BRCA1_Intron_12__region_1__2 | 17 | 41237543 | 41237725 | ACACTGACGACATGGTTCTACACCCCTCACACAAGTTGTTTTCCT | TACGGTAGCAGAGACTTGGTCTTCATATTTCACAACTCCTCCTGGT | 183 | 42 | CCCCTCACACAAGTTGTTTTCCTATAACACATGACAACTTTACGTTTCCTGGGAATTCCATGACTCTGTACCTTTACCTTACTTGCTGCCACTCCTCTTTTTCCACTTGGAAAAATTCTCATCTTTCAAGGCCTAGTTTGAATGTCTCCTTAAGTCCCCACCAGGAGGAGTTGTGAAATATGA |  |
| BRCA1 | BRCA1_Intron_12__region_1__3 | 17 | 41237628 | 41237825 | ACACTGACGACATGGTTCTACACTGCCACTCCTCTTTTTCCACT | TACGGTAGCAGAGACTTGGTCTTATACACTCTGCTTCCCTCCAGA | 198 | 46 | CTGCCACTCCTCTTTTTCCACTTGGAAAAATTCTCATCTTTCAAGGCCTAGTTTGAATGTCTCCTTAAGTCCCCACCAGGAGGAGTTGTGAAATATGAGAAGTAAGGGCAAGTGGGCAGAATGTGGCTAATTCTAATAAGACTCCTGAAATGTGCAGCAGAGCAGCCGGAACTGGTCTGGAGGGAAGCAGAGTGTATA |  |
| BRCA1 | BRCA1_Intron_12__region_1__4 | 17 | 41237700 | 41237896 | ACACTGACGACATGGTTCTACACCACCAGGAGGAGTTGTGAAATA | TACGGTAGCAGAGACTTGGTCTATACACATGTTTTATGGGAAGGTTTT | 197 | 41 | CCACCAGGAGGAGTTGTGAAATATGAGAAGTAAGGGCAAGTGGGCAGAATGTGGCTAATTCTAATAAGACTCCTGAAATGTGCAGCAGAGCAGCCGGAACTGGTCTGGAGGGAAGCAGAGTGTATATCTGACCCTTGTTTGTTCACATTCATTACATATAAATATATATATAAAACCTTCCCATAAAACATGTGTAT |  |
| BRCA1 | BRCA1_19 | 17 | 41242903 | 41243101 | ACACTGACGACATGGTTCTACACTACTGAATGCAAAGGACACCAC | TACGGTAGCAGAGACTTGGTCTGCAGCGTTTATAGTCTGCTTTTAC | 199 | 45 | CTACTGAATGCAAAGGACACCACACACACGCATGTGCACACACACACACGCTTTTTACCTGAGTGGTTAAAATGTCACTCTGAGAGGATAGCCCTGAGCAGTCTTCAGAGACGCTTGTTTCACTCTCACACCCAGATGCTGCTTCACCTTAAATAACAAAAACAGAGGTTCAGATGTAAAAGCAGACTATAAACGCTGC |  |
| BRCA1 | BRCA1_20 | 17 | 41243373 | 41243569 | ACACTGACGACATGGTTCTACAATGTGCTCCCCAAAAGCATAAAC | TACGGTAGCAGAGACTTGGTCTCAGTCTGAAAGCCAGGGAGTTG | 197 | 41 | ATGTGCTCCCCAAAAGCATAAACATTTAGCTCACTTCTATAAATAGACTGGGGCAAACACAAAAACCTGGTTCCAATACCTAAGTTTGAATCCATGCTTTGCTCTTCTTGATTATTTTCTTCCAAGCCCGTTCCTCTTTCTTCATCATCTGAAACCAATTCCTTGTCACTCAGACCAACTCCCTGGCTTTCAGACTG |  |
| BRCA1 | BRCA1_21 | 17 | 41243485 | 41243674 | ACACTGACGACATGGTTCTACATATTTTCTTCCAAGCCCGTTCCT | TACGGTAGCAGAGACTTGGTCTTCTGCTAGCTTGTTTTCTTCACA | 190 | 43 | TATTTTCTTCCAAGCCCGTTCCTCTTTCTTCATCATCTGAAACCAATTCCTTGTCACTCAGACCAACTCCCTGGCTTTCAGACTGATGCCTCATTTGTTTGGAAGAACCAATCAAGAAAGGATCCTGGGTGTTTGTATTTGCAGTCAAGTCTTCCAATTCACTGCACTGTGAAGAAAACAAGCTAGCAGA | Assays designed by relax mode and have no off-target hits |
| BRCA1 | BRCA1_22 | 17 | 41243604 | 41243793 | ACACTGACGACATGGTTCTACAGGATCCTGGGTGTTTGTATTTGC | TACGGTAGCAGAGACTTGGTCTTGTCTAAGAACACAGAGGAGAATTTA | 190 | 39 | GGATCCTGGGTGTTTGTATTTGCAGTCAAGTCTTCCAATTCACTGCACTGTGAAGAAAACAAGCTAGCAGAACATTTTGTTTCCTCACTAAGGTGATGTTCCTGAGATGCCTTTGCCAATATTACCTGGTTACTGCAGTCATTTAAGCTATTCTTCAATGATAATAAATTCTCCTCTGTGTTCTTAGACA | Assays designed by relax mode and have no off-target hits |
| BRCA1 | BRCA1_23 | 17 | 41243699 | 41243897 | ACACTGACGACATGGTTCTACAATGTTCCTGAGATGCCTTTGCC | TACGGTAGCAGAGACTTGGTCTTAGTGAGGATGAAGAGCTTCCCT | 199 | 40 | ATGTTCCTGAGATGCCTTTGCCAATATTACCTGGTTACTGCAGTCATTTAAGCTATTCTTCAATGATAATAAATTCTCCTCTGTGTTCTTAGACAGACACTCGGTAGCAACGGTGCTATGCCTAGTAGACTGAGAAGGTATATTGTTTACTTTACCAAATAACAAGTGTTGGAAGCAGGGAAGCTCTTCATCCTCACTA | Assays designed by relax mode and have no off-target hits |
| BRCA1 | BRCA1_24 | 17 | 41243852 | 41244044 | ACACTGACGACATGGTTCTACAACCAAATAACAAGTGTTGGAAGC | TACGGTAGCAGAGACTTGGTCTTGACATTAAGGAAAGTTCTGCTGTT | 193 | 45 | ACCAAATAACAAGTGTTGGAAGCAGGGAAGCTCTTCATCCTCACTAGATAAGTTCTCTTCTGAGGACTCTAATTTCTTGGCCCCTCTTCGGTAACCCTGAGCCAAATGTGTATGGGTGAAAGGGCTAGGACTCCTGCTAAGCTCTCCTTTCTGGACGCTTTTGCTAAAAACAGCAGAACTTTCCTTAATGTCA | Assays designed by relax mode and have no off-target hits |
| BRCA1 | BRCA1_25 | 17 | 41243949 | 41244145 | ACACTGACGACATGGTTCTACATGAGCCAAATGTGTATGGGTGAA | TACGGTAGCAGAGACTTGGTCTCAGCCTATGGGAAGTAGTCATGC | 197 | 43 | TGAGCCAAATGTGTATGGGTGAAAGGGCTAGGACTCCTGCTAAGCTCTCCTTTCTGGACGCTTTTGCTAAAAACAGCAGAACTTTCCTTAATGTCATTTTCAGCAAAACTAGTATCTTCCTTTATTTCACCATCATCTAACAGGTCATCAGGTGTCTCAGAACAAACCTGAGATGCATGACTACTTCCCATAGGCTG | Assays designed by relax mode and have no off-target hits |
| BRCA1 | BRCA1_26 | 17 | 41244007 | 41244197 | ACACTGACGACATGGTTCTACACGCTTTTGCTAAAAACAGCAGAAC | TACGGTAGCAGAGACTTGGTCTTCAGACTGTTAATACAGATTTCTCTCCA | 191 | 37 | CGCTTTTGCTAAAAACAGCAGAACTTTCCTTAATGTCATTTTCAGCAAAACTAGTATCTTCCTTTATTTCACCATCATCTAACAGGTCATCAGGTGTCTCAGAACAAACCTGAGATGCATGACTACTTCCCATAGGCTGTTCTAAGTTATCTGAAATCAGATATGGAGAGAAATCTGTATTAACAGTCTGA | Assays designed by relax mode and have no off-target hits |
| BRCA1 | BRCA1_27 | 17 | 41244115 | 41244295 | ACACTGACGACATGGTTCTACACCTGAGATGCATGACTACTTCCC | TACGGTAGCAGAGACTTGGTCTAGATTAGGGGTTTTGCAACCTGA | 181 | 36 | CCTGAGATGCATGACTACTTCCCATAGGCTGTTCTAAGTTATCTGAAATCAGATATGGAGAGAAATCTGTATTAACAGTCTGAACTACTTCTTCATATTCTTGCTTTTTTATTTCAGGATGCTTACAATTACTTCCAGGAAGACTTTGTTTATAGACCTCAGGTTGCAAAACCCCTAATCT | Assays designed by relax mode and have no off-target hits |
| BRCA1 | BRCA1_28 | 17 | 41244171 | 41244361 | ACACTGACGACATGGTTCTACAGGAGAGAAATCTGTATTAACAGTCTGAAC | TACGGTAGCAGAGACTTGGTCTTCCAGTGATGAAAACATTCAAGCA | 191 | 37 | GGAGAGAAATCTGTATTAACAGTCTGAACTACTTCTTCATATTCTTGCTTTTTTATTTCAGGATGCTTACAATTACTTCCAGGAAGACTTTGTTTATAGACCTCAGGTTGCAAAACCCCTAATCTAAGCATAGCATTCAATTTTGGCCCTCTGTTTCTACCTAGTTCTGCTTGAATGTTTTCATCactgga | Assays designed by relax mode and have no off-target hits |
| BRCA1 | BRCA1_29 | 17 | 41244266 | 41244448 | ACACTGACGACATGGTTCTACAATAGACCTCAGGTTGCAAAACCC | TACGGTAGCAGAGACTTGGTCTAATGTTTTTAAAGAAGCCAGCTCA | 183 | 38 | ATAGACCTCAGGTTGCAAAACCCCTAATCTAAGCATAGCATTCAATTTTGGCCCTCTGTTTCTACCTAGTTCTGCTTGAATGTTTTCATCactggaacctatttcattaatactggagcccacttcattagtactggaacctacttcattaataTTGCTTGAGCTGGCTTCTTTAAAAACATT | Assays designed by relax mode and have no off-target hits |
| BRCA1 | BRCA1_30 | 17 | 41244375 | 41244557 | ACACTGACGACATGGTTCTACAATACTGGAGCCCACTTCATTAGT | TACGGTAGCAGAGACTTGGTCTTCTGCTAGAGGAAAACTTTGAGGA | 183 | 37 | atactggagcccacttcattagtactggaacctacttcattaataTTGCTTGAGCTGGCTTCTTTAAAAACATTTTCTCTAATGTTATTACGGCTAATTGTGCTCACTGTACTTGGAATGTTCTCATTTCCCATTTCTCTTTCAGGTGACATTGAATGTTCCTCAAAGTTTTCCTCTAGCAGA | Assays designed by relax mode and have no off-target hits |
| BRCA1 | BRCA1_31 | 17 | 41244473 | 41244671 | ACACTGACGACATGGTTCTACATGTGCTCACTGTACTTGGAATGT | TACGGTAGCAGAGACTTGGTCTAGGCAACGAAACTGGACTCATTA | 199 | 36 | TGTGCTCACTGTACTTGGAATGTTCTCATTTCCCATTTCTCTTTCAGGTGACATTGAATGTTCCTCAAAGTTTTCCTCTAGCAGATTTTTCTTACATTTAGTTTTAACAAATGACTTGATGGGAAAAAGTGGTGGTATACGATATGGGTTTTGTAAAAGTCCATGTTTATTTGGAGTAATGAGTCCAGTTTCGTTGCCT | Assays designed by relax mode and have no off-target hits |
| BRCA1 | BRCA1_32 | 17 | 41244586 | 41244763 | ACACTGACGACATGGTTCTACAACTTGATGGGAAAAAGTGGTGGT | TACGGTAGCAGAGACTTGGTCTTTTCCTGTGGTTGGTCAGAAAGA | 178 | 39 | ACTTGATGGGAAAAAGTGGTGGTATACGATATGGGTTTTGTAAAAGTCCATGTTTATTTGGAGTAATGAGTCCAGTTTCGTTGCCTCTGAACTGAGATGATAGACAAAACCTAGAGCCTCCTTTGATACTACATTTGGCATTATCAACTGGCTTATCTTTCTGACCAACCACAGGAAA | Assays designed by relax mode and have no off-target hits |
| BRCA1 | BRCA1_33 | 17 | 41244648 | 41244841 | ACACTGACGACATGGTTCTACAGTAATGAGTCCAGTTTCGTTGCC | TACGGTAGCAGAGACTTGGTCTTGTGAACAAAAGGAAGAAAATCAAGGA | 194 | 40 | GTAATGAGTCCAGTTTCGTTGCCTCTGAACTGAGATGATAGACAAAACCTAGAGCCTCCTTTGATACTACATTTGGCATTATCAACTGGCTTATCTTTCTGACCAACCACAGGAAAGCCTGCAGTGATATTAACTGTCTGTACAGGCTTGATATTAGACTCATTCTTTCCTTGATTTTCTTCCTTTTGTTCACA | Assays designed by relax mode and have no off-target hits |
| BRCA1 | BRCA1_34 | 17 | 41244742 | 41244940 | ACACTGACGACATGGTTCTACACTTTCTGACCAACCACAGGAAAG | TACGGTAGCAGAGACTTGGTCTGCTCCGTTTTCAAATCCAGGAAA | 199 | 41 | CTTTCTGACCAACCACAGGAAAGCCTGCAGTGATATTAACTGTCTGTACAGGCTTGATATTAGACTCATTCTTTCCTTGATTTTCTTCCTTTTGTTCACATTCAAAAGTGACTTTTGGACTTTGTTTCTTTAAGGACCCAGAGTGGGCAGAGAATGTTGCACATTCCTCTTCTGCATTTCCTGGATTTGAAAACGGAGC | Assays designed by relax mode and have no off-target hits |
| BRCA1 | BRCA1_35 | 17 | 41244864 | 41245040 | ACACTGACGACATGGTTCTACATGTTTCTTTAAGGACCCAGAGTG | TACGGTAGCAGAGACTTGGTCTAGTTAACCACAGTCGGGAAACAA | 177 | 42 | TGTTTCTTTAAGGACCCAGAGTGGGCAGAGAATGTTGCACATTCCTCTTCTGCATTTCCTGGATTTGAAAACGGAGCAAATGACTGGCGCTTTGAAACCTTGAATGTATTCTGCAAATACTGAGCATCAAGTTCACTTTCTTCCATTTCTATGCTTGTTTCCCGACTGTGGTTAACT | Assays designed by relax mode and have no off-target hits |
| BRCA1 | BRCA1_36 | 17 | 41244947 | 41245144 | ACACTGACGACATGGTTCTACACTGGCGCTTTGAAACCTTGAAT | TACGGTAGCAGAGACTTGGTCTGTGAGTCAGTGTGCAGCATTTG | 198 | 40 | CTGGCGCTTTGAAACCTTGAATGTATTCTGCAAATACTGAGCATCAAGTTCACTTTCTTCCATTTCTATGCTTGTTTCCCGACTGTGGTTAACTTCATGTCCCAATGGATACTTAAAGCCTTCTGTGTCATTTCTATTATCTTTGGAACAACCATGAATTAGTCCCTTGGGGTTTTCAAATGCTGCACACTGACTCAC | Assays designed by relax mode and have no off-target hits |
| BRCA1 | BRCA1_37 | 17 | 41245044 | 41245232 | ACACTGACGACATGGTTCTACATGTCCCAATGGATACTTAAAGCC | TACGGTAGCAGAGACTTGGTCTACCTGGTACTGATTATGGCACTC | 189 | 41 | TGTCCCAATGGATACTTAAAGCCTTCTGTGTCATTTCTATTATCTTTGGAACAACCATGAATTAGTCCCTTGGGGTTTTCAAATGCTGCACACTGACTCACACATTTATTTGGTTCTGTTTTTGCCTTCCCTAGAGTGCTAACTTCCAGTAACGAGATACTTTCCTGAGTGCCATAATCAGTACCAGGT | Assays designed by relax mode and have no off-target hits |
| BRCA1 | BRCA1_38 | 17 | 41245154 | 41245339 | ACACTGACGACATGGTTCTACATGGTTCTGTTTTTGCCTTCCCTA | TACGGTAGCAGAGACTTGGTCTACAGTTAAAGTGTCTAATAATGCTGAAGA | 186 | 40 | TGGTTCTGTTTTTGCCTTCCCTAGAGTGCTAACTTCCAGTAACGAGATACTTTCCTGAGTGCCATAATCAGTACCAGGTACCAATGAAATACTGCTACTCTCTACAGATCTTTCAGTTTGCAAAACCCTTTCTCCACTTAACATGAGATCTTTGGGGTCTTCAGCATTATTAGACACTTTAACTGT | Assays designed by relax mode and have no off-target hits |
| BRCA1 | BRCA1_39 | 17 | 41245246 | 41245433 | ACACTGACGACATGGTTCTACATGCTACTCTCTACAGATCTTTCAGTT | TACGGTAGCAGAGACTTGGTCTACCTGGTTCTTTTACTAAGTGTTCAAA | 188 | 36 | TGCTACTCTCTACAGATCTTTCAGTTTGCAAAACCCTTTCTCCACTTAACATGAGATCTTTGGGGTCTTCAGCATTATTAGACACTTTAACTGTTTCTAGTTTCTCTTCTTTTTCTTCTCTTGGAAGGCTAGGATTGACAAATTCTTTAAGTTCACTGGTATTTGAACACTTAGTAAAAGAACCAGGT | Assays designed by relax mode and have no off-target hits |
| BRCA1 | BRCA1_40 | 17 | 41245357 | 41245539 | ACACTGACGACATGGTTCTACATTTCTTCTCTTGGAAGGCTAGGA | TACGGTAGCAGAGACTTGGTCTAAGGTAAAGAACCTGCAACTGGA | 183 | 39 | TTTCTTCTCTTGGAAGGCTAGGATTGACAAATTCTTTAAGTTCACTGGTATTTGAACACTTAGTAAAAGAACCAGGTGCATTTGTTAACTTCAGCTCTGGGAAAGTATCGCTGTCATGTCTTTTACTTGTCTGTTCATTTGGCTTGTTACTCTTCTTGGCTCCAGTTGCAGGTTCTTTACCTT | Assays designed by relax mode and have no off-target hits |
| BRCA1 | BRCA1_41 | 17 | 41245455 | 41245653 | ACACTGACGACATGGTTCTACAGGGAAAGTATCGCTGTCATGTCT | TACGGTAGCAGAGACTTGGTCTGCCCACCTAATTGTACTGAATTGC | 199 | 41 | GGGAAAGTATCGCTGTCATGTCTTTTACTTGTCTGTTCATTTGGCTTGTTACTCTTCTTGGCTCCAGTTGCAGGTTCTTTACCTTCCATGAGTTGTAGGTTTCTGCTGTGCCTGACTGGCATTTGGTTGTACTTTTTTTTCTTTATCTCTTCACTGCTAGAACAACTATCAATTTGCAATTCAGTACAATTAGGTGGGC | Assays designed by relax mode and have no off-target hits |
| BRCA1 | BRCA1_42 | 17 | 41245543 | 41245737 | ACACTGACGACATGGTTCTACATGAGTTGTAGGTTTCTGCTGTGC | TACGGTAGCAGAGACTTGGTCTAAGCACCTAAAAAGAATAGGCTGA | 195 | 39 | TGAGTTGTAGGTTTCTGCTGTGCCTGACTGGCATTTGGTTGTACTTTTTTTTCTTTATCTCTTCACTGCTAGAACAACTATCAATTTGCAATTCAGTACAATTAGGTGGGCTTAGATTTCTACTGACTACTAGTTCAAGCGCATGAATATGCCTGGTAGAAGACTTCCTCCTCAGCCTATTCTTTTTAGGTGCTT | Assays designed by relax mode and have no off-target hits |
| BRCA1 | BRCA1_43 | 17 | 41245646 | 41245835 | ACACTGACGACATGGTTCTACAAGGTGGGCTTAGATTTCTACTGAC | TACGGTAGCAGAGACTTGGTCTAGAATCACTCGAAAAAGAATCTGC | 190 | 38 | AGGTGGGCTTAGATTTCTACTGACTACTAGTTCAAGCGCATGAATATGCCTGGTAGAAGACTTCCTCCTCAGCCTATTCTTTTTAGGTGCTTTTGAATTGTGGATATTTAATTCGAGTTCCATATTGCTTATACTGCTGCTTATAGGTTCAGCTTTCGTTTTGAAAGCAGATTCTTTTTCGAGTGATTCT | Assays designed by relax mode and have no off-target hits |
| BRCA1 | BRCA1_44 | 17 | 41245703 | 41245894 | ACACTGACGACATGGTTCTACAAGACTTCCTCCTCAGCCTATTCT | TACGGTAGCAGAGACTTGGTCTGGTCATGAGAATAAAACAAAAGGTGATTCT | 192 | 35 | AGACTTCCTCCTCAGCCTATTCTTTTTAGGTGCTTTTGAATTGTGGATATTTAATTCGAGTTCCATATTGCTTATACTGCTGCTTATAGGTTCAGCTTTCGTTTTGAAAGCAGATTCTTTTTCGAGTGATTCTATTGGGTTAGGATTTTTCTCATTCTGAATAGAATCACCTTTTGTTTTATTCTCATGACC | Assays designed by relax mode and have no off-target hits |
| BRCA1 | BRCA1_45 | 17 | 41245824 | 41245994 | ACACTGACGACATGGTTCTACATCGAGTGATTCTATTGGGTTAGGA | TACGGTAGCAGAGACTTGGTCTCAAGAAAGCAGATTTGGCAGTTCA | 171 | 36 | TCGAGTGATTCTATTGGGTTAGGATTTTTCTCATTCTGAATAGAATCACCTTTTGTTTTATTCTCATGACCACTATTAGTAATATTCATCACTTGACCATTCTGCTCCGTTTGGTTAGTTCCCTGATTTATCATTTCAGGAGTCTTTTGAACTGCCAAATCTGCTTTCTTG | Assays designed by relax mode and have no off-target hits |
| BRCA1 | BRCA1_46 | 17 | 41245929 | 41246123 | ACACTGACGACATGGTTCTACATCCGTTTGGTTAGTTCCCTGATTT | TACGGTAGCAGAGACTTGGTCTCCATGTAACTGAAAATCTAATTATAGGAGCA | 195 | 38 | TCCGTTTGGTTAGTTCCCTGATTTATCATTTCAGGAGTCTTTTGAACTGCCAAATCTGCTTTCTTGATAAAATCCTCAGGATGAAGGCCTGATGTAGGTCTCCTTTTACGCTTTAATTTATTTGTGAGGGGACGCTCTTGTATTATCTGTGGCTCAGTAACAAATGCTCCTATAATTAGATTTTCAGTTACATGG | Assays designed by relax mode and have no off-target hits |
| BRCA1 | BRCA1_47 | 17 | 41246014 | 41246212 | ACACTGACGACATGGTTCTACAGGCCTGATGTAGGTCTCCTTTTA | TACGGTAGCAGAGACTTGGTCTAGAGTTCACTCCAAATCAGTAGAG | 199 | 39 | GGCCTGATGTAGGTCTCCTTTTACGCTTTAATTTATTTGTGAGGGGACGCTCTTGTATTATCTGTGGCTCAGTAACAAATGCTCCTATAATTAGATTTTCAGTTACATGGCTTAAGTTGGGGAGGCTTGCCTTCTTCCGATAGGTTTTCCCAAATATTTTGTCTTCAATATTACTCTCTACTGATTTGGAGTGAACTCT | Assays designed by relax mode and have no off-target hits |
| BRCA1 | BRCA1_48 | 17 | 41246138 | 41246324 | ACACTGACGACATGGTTCTACAGCTTGCCTTCTTCCGATAGGT | TACGGTAGCAGAGACTTGGTCTAGTAGCTGATGTATTGGACGTTCT | 187 | 37 | GCTTGCCTTCTTCCGATAGGTTTTCCCAAATATTTTGTCTTCAATATTACTCTCTACTGATTTGGAGTGAACTCTTTCACTTTTACATATTAAAGCCTCATGAGGATCACTGGCCAGTAAGTCTATTTTCTCTGAAGAACCAGAATATTCATCTACCTCATTTAGAACGTCCAATACATCAGCTACT | Assays designed by relax mode and have no off-target hits |
| BRCA1 | BRCA1_49 | 17 | 41246190 | 41246388 | ACACTGACGACATGGTTCTACATCTACTGATTTGGAGTGAACTCTTT | TACGGTAGCAGAGACTTGGTCTCCAGAAGTGATGAACTGTTAGGT | 199 | 39 | TCTACTGATTTGGAGTGAACTCTTTCACTTTTACATATTAAAGCCTCATGAGGATCACTGGCCAGTAAGTCTATTTTCTCTGAAGAACCAGAATATTCATCTACCTCATTTAGAACGTCCAATACATCAGCTACTTTGGCATTTGATTCAGACTCCCCATCATGTGAGTCATCAGAACCTAACAGTTCATCACTTCTGG | Assays designed by relax mode and have no off-target hits |
| BRCA1 | BRCA1_50 | 17 | 41246313 | 41246488 | ACACTGACGACATGGTTCTACAACATCAGCTACTTTGGCATTTGA | TACGGTAGCAGAGACTTGGTCTAATAAGCAGAAACTGCCATGCTC | 176 | 40 | ACATCAGCTACTTTGGCATTTGATTCAGACTCCCCATCATGTGAGTCATCAGAACCTAACAGTTCATCACTTCTGGAAAACCACTCATTAACTTTCTGAATGCTGCTATTTAGTGTTATCCAAGGAACATCTTCAGTATCTCTAGGATTCTCTGAGCATGGCAGTTTCTGCTTATT | Assays designed by relax mode and have no off-target hits |
| BRCA1 | BRCA1_51 | 17 | 41246413 | 41246606 | ACACTGACGACATGGTTCTACATGCTGCTATTTAGTGTTATCCAAGG | TACGGTAGCAGAGACTTGGTCTAAGGAGCCAACATAACAGATGGG | 194 | 43 | TGCTGCTATTTAGTGTTATCCAAGGAACATCTTCAGTATCTCTAGGATTCTCTGAGCATGGCAGTTTCTGCTTATTCCATTCTTTTCTCTCACACAGGGGATCAGCATTCAGATCTACCTTTTTTTCTGTGCTGGGAGTCCGCCTATCATTACATGTTTCCTTACTTCCAGCCCATCTGTTATGTTGGCTCCTT | Assays designed by relax mode and have no off-target hits |
| BRCA1 | BRCA1_52 | 17 | 41246501 | 41246699 | ACACTGACGACATGGTTCTACACTCACACAGGGGATCAGCATTC | TACGGTAGCAGAGACTTGGTCTACAGCATGAGAACAGCAGTTTATT | 199 | 42 | CTCACACAGGGGATCAGCATTCAGATCTACCTTTTTTTCTGTGCTGGGAGTCCGCCTATCATTACATGTTTCCTTACTTCCAGCCCATCTGTTATGTTGGCTCCTTGCTAAGCCAGGCTGTTTGCTTTTATTACAGAATTCAGCCTTTTCTACATTCATTCTGTCTTTAGTGAGTAATAAACTGCTGTTCTCATGCTGT | Assays designed by relax mode and have no off-target hits |
| BRCA1 | BRCA1_53 | 17 | 41246584 | 41246781 | ACACTGACGACATGGTTCTACACCCATCTGTTATGTTGGCTCCTT | TACGGTAGCAGAGACTTGGTCTGGCATCCAGAAAAGTATCAGGGTA | 198 | 42 | CCCATCTGTTATGTTGGCTCCTTGCTAAGCCAGGCTGTTTGCTTTTATTACAGAATTCAGCCTTTTCTACATTCATTCTGTCTTTAGTGAGTAATAAACTGCTGTTCTCATGCTGTAATGAGCTGGCATGAGTATTTGTGCCACATGGCTCCACATGCAAGTTTGAAACAGAACTACCCTGATACTTTTCTGGATGCC | Assays designed by relax mode and have no off-target hits |
| BRCA1 | BRCA1_54 | 17 | 41246695 | 41246882 | ACACTGACGACATGGTTCTACAGCTGTAATGAGCTGGCATGAGTA | TACGGTAGCAGAGACTTGGTCTTTCAGCTGCTTGTGAATTTTCTG | 188 | 43 | GCTGTAATGAGCTGGCATGAGTATTTGTGCCACATGGCTCCACATGCAAGTTTGAAACAGAACTACCCTGATACTTTTCTGGATGCCTCTCAGCTGCACGCTTCTCAGTGGTGTTCAAATCATTATTACTGGGTTGATGATGTTCAGTATTTGTTACATCCGTCTCAGAAAATTCACAAGCAGCTGAA | Assays designed by relax mode and have no off-target hits |
| BRCA1 | BRCA1_55 | 17 | 41246761 | 41246959 | ACACTGACGACATGGTTCTACACCTGATACTTTTCTGGATGCCTCT | TACGGTAGCAGAGACTTGGTCTCCACCTCCAAGGTGTATGAAGTA | 199 | 37 | CCTGATACTTTTCTGGATGCCTCTCAGCTGCACGCTTCTCAGTGGTGTTCAAATCATTATTACTGGGTTGATGATGTTCAGTATTTGTTACATCCGTCTCAGAAAATTCACAAGCAGCTGAAAATATACAAAAATAACAAGGTACTCAAAAACTGAATTGTCATTAAAAAAATACATACTTCATACACCTTGGAGGTGG | Assays designed by relax mode and have no off-target hits |
| BRCA1 | BRCA1_56 | 17 | 41247808 | 41248006 | ACACTGACGACATGGTTCTACATGTATCTACCCACTCTCTTTTCAGT | TACGGTAGCAGAGACTTGGTCTGGTCATTTGACAGTTCTGCATAC | 199 | 40 | TGTATCTACCCACTCTCTTTTCAGTGCCTGTTAAGTTGGCAAACTTTGCCATTACCCTTTTTTGCAGAATCCAAACTGATTTCATCCCTGGTTCCTTGAGGGGTGATTTGTAACAATTCTTGATCTCCCACACTATAGGGAAAAGACAGAGTCCTAATAAGAAACACTAGTTACATGTATGCAGAACTGTCAAATGACC |  |
| BRCA1 | BRCA1_57 | 17 | 41249130 | 41249324 | ACACTGACGACATGGTTCTACAACAAACTGCACATACATCCCTGA | TACGGTAGCAGAGACTTGGTCTTGGGGGGAAATTTTTTAGGATCTG | 195 | 32 | acaaactgcacatacatccctgaacctaaaataaaagttaaaaTATTTTTAAAAAGAGAGAAACATCAATCCTTAATATTAACTAAATAGGAAAATACCAGCTTCATAGACAAAGGTTCTCTTTGACTCACCTGCAATAAGTTGCCTTATTAACGGTATCTTCAGAAGAATCAGATCCTAAAAAATTTCCCCCCA | Assays designed by relax mode and have no off-target hits |
| BRCA1 | BRCA1_58 | 17 | 41249255 | 41249439 | ACACTGACGACATGGTTCTACAACTCACCTGCAATAAGTTGCCTT | TACGGTAGCAGAGACTTGGTCTGCATTGTACCTGCCACAGTAGAT | 185 | 31 | ACTCACCTGCAATAAGTTGCCTTATTAACGGTATCTTCAGAAGAATCAGATCCTAAAAAATTTCCCCCCAAAAAATAAATCAATAAAAGTTTTCTTAATTAAAAGGGTTAAAAAAATGTACTTGTTGAAAAACAGATattcaactagaaatatttactgagcatctactgtggcaggtacaatgc | Assays designed by relax mode and have no off-target hits |
| BRCA1 | BRCA1_59 | 17 | 41251674 | 41251855 | ACACTGACGACATGGTTCTACAATTCACTTCCCAAAGCTGCCTAC | TACGGTAGCAGAGACTTGGTCTGTGAGAACTCTGAGGACAAAGCA | 182 | 37 | ATTCACTTCCCAAAGCTGCCTACCACAAATACAAATTATGACCAAGATTTTTGGCAAAACTATAAGATAAGGAATCCAGCAATTATTATTAAATACTTAAAAAACCTGAGACCCTTACCCAATTCAATGTAGACAGACGTCTTTTGAGGTTGTATCCGCTGCTTTGTCCTCAGAGTTCTCAC | Assays designed by relax mode and have no off-target hits |
| BRCA1 | BRCA1_60 | 17 | 41251773 | 41251963 | ACACTGACGACATGGTTCTACAAAAAACCTGAGACCCTTACCCAA | TACGGTAGCAGAGACTTGGTCTCTCTTCAGGAGGAAAAGCACAGA | 191 | 45 | AAAAACCTGAGACCCTTACCCAATTCAATGTAGACAGACGTCTTTTGAGGTTGTATCCGCTGCTTTGTCCTCAGAGTTCTCACAGTTCCAAGGTTAGAGAGTTGGACACTGAGACTGGTTTCCTGCTAAACAGTATGGTAAAGAACAGTCAAGCAATTGTTGGCCAGTTCTGTGCTTTTCCTCCTGAAGAG | Assays designed by relax mode and have no off-target hits |
| BRCA1 | BRCA1_61 | 17 | 41256105 | 41256293 | ACACTGACGACATGGTTCTACAAGAAGAAGAAGAAGAAGAAAACAAATGG | TACGGTAGCAGAGACTTGGTCTCCTTGTATTTTACAGATGCAAACAGC | 189 | 35 | agaagaagaagaagaagaaAACAAATGGTTTTACCAAGGAAGGATTTTCGGGTTCACTCTGTAGAAGTCTTTTGGCACGGTTTCTGTAGCCCATACTTTGGATGATAGAAACTTCATCTTTTAGATGTTCAGGAGAGTTATTTTCCTTTTTTGCAAAATTATAGCTGTTTGCATCTGTAAAATACAAGG | Assays designed by relax mode and have no off-target hits |
| BRCA1 | BRCA1_62 | 17 | 41256170 | 41256368 | ACACTGACGACATGGTTCTACAAGTCTTTTGGCACGGTTTCTGTA | TACGGTAGCAGAGACTTGGTCTGCATACATAGGGTTTCTCTTGGT | 199 | 33 | AGTCTTTTGGCACGGTTTCTGTAGCCCATACTTTGGATGATAGAAACTTCATCTTTTAGATGTTCAGGAGAGTTATTTTCCTTTTTTGCAAAATTATAGCTGTTTGCATCTGTAAAATACAAGGGAAAACATTATGTTTGCAGTTAGAGAAAAATGTATGAATTATAATCAAAGAAACCAAGAGAAACCCTATGTATGC | Assays designed by relax mode and have no off-target hits |
| BRCA1 | BRCA1_63 | 17 | 41256828 | 41257012 | ACACTGACGACATGGTTCTACATTCCTGAGTTTTCATGGACAGCA | TACGGTAGCAGAGACTTGGTCTTCACTTGCTGAGTGTGTTTCTCA | 185 | 38 | TTCCTGAGTTTTCATGGACAGCACTTGAGTGTCATTCTTGGGATATTCAACACTTACACTCCAAACCTGTGTCAAGCTGAAAAGCACAAATGATTTTCAATAGCTCTTCAACAAGTTGACTAAATCTCGTACTTTCTTGTAGGCTCCTGAAATTAAATTGTTTGAGAAACACACTCAGCAAGTGA |  |
| BRCA1 | BRCA1_64 | 17 | 41258420 | 41258615 | ACACTGACGACATGGTTCTACAACTTTTTCCTACTGTGGTTGCTT | TACGGTAGCAGAGACTTGGTCTTTTTGAGTATTCTTTCTACAAAAGGAAGTAAAT | 196 | 31 | ACTTTTTCCTACTGTGGTTGCTTCCAACCTAGCATCATTACCAAATTATATACCTTTTGGTTATATCATTCTTACATAAAGGACACTGTGAAGGCCCTTTCTTCTGGTTGAGAAGTTTCAGCATGCAAAATCTATAAATTATAAAGAAAGAAAGAACAATTTAATTTACTTCCTTTTGTAGAAAGAATACTCAAAA |  |
| BRCA1 | BRCA1_65 | 17 | 41267715 | 41267905 | ACACTGACGACATGGTTCTACATGGAGCCACATAACACATTCAAA | TACGGTAGCAGAGACTTGGTCTACTCAGTCATAACAGCTCAAAGT | 191 | 37 | TGGAGCCACATAACACATTCAAACTTACTTGCAAAATATGTGGTCACACTTTGTGGAGACAGGTTCCTTGATCAACTCCAGACTAGCAGGGTAGGGGGGGAGAAAAAGAAAATAAATGAGGCTCaataatttatttaaaaataaaGCTATTCTTAGTGAATAAGTTCAACTTTGAGCTGTTATGACTGAGT |  |
| BRCA1 | BRCA1_Intron_2__region_1__1 | 17 | 41271145 | 41271333 | ACACTGACGACATGGTTCTACATGTGACAAGAATGTGGTTTTTTCCT | TACGGTAGCAGAGACTTGGTCTTCCACAAGCTTTTCTTGTGATCC | 189 | 42 | TGTGACAAGAATGTGGTTTTTTCCTTAAATATTTAACTTTTTAGAAAAGGATCACAAGggccaggtgcggtggctcacgctgtaatcccagcattttgggaggccaaggcgggccagcctgggtgacagagaatccatctcaaaaaaagaaaaaaaaaaaagaaaaGGATCACAAGAAAAGCTTGTGGA |  |
| BRCA1 | BRCA1_Intron_2__region_1__2 | 17 | 41271314 | 41271508 | ACACTGACGACATGGTTCTACATCACAAGAAAAGCTTGTGGACAG | TACGGTAGCAGAGACTTGGTCTGAAACAGGTTCAGGTAGAGTGGT | 195 | 42 | TCACAAGAAAAGCTTGTGGACAGTAACCTTATTGTGAAGGGTTGTAATACAACTCTTGTAATCATGGGGTTTTTGACATAGCACAGGgcagtgaaaagaaaaacaatgaactaagtcaggaggctgggtttctactaccagttgtgtatataagcagagccaccttgggctaaccactctacctgaacctgtttc | One primer sits in the repeat region |
| BRCA1 | BRCA1_Intron_2__region_1__3 | 17 | 41271383 | 41271556 | ACACTGACGACATGGTTCTACATTTTTGACATAGCACAGGGCAGT | TACGGTAGCAGAGACTTGGTCTATTCTTGCAATAGCCCAAGGAGT | 174 | 45 | TTTTTGACATAGCACAGGgcagtgaaaagaaaaacaatgaactaagtcaggaggctgggtttctactaccagttgtgtatataagcagagccaccttgggctaaccactctacctgaacctgtttccttctcttgccattcaccctgccagactccttgggctattgcaagaat | Two primers sits in the repeat region |
| BRCA1 | BRCA1_Intron_2__region_1__4 | 17 | 41271482 | 41271675 | ACACTGACGACATGGTTCTACAGCTAACCACTCTACCTGAACCTG | TACGGTAGCAGAGACTTGGTCTAAAGTCACATTTGGTCCCAGTAA | 194 | 41 | gctaaccactctacctgaacctgtttccttctcttgccattcaccctgccagactccttgggctattgcaagaataaaattaaatgctacttgggaaaatgcttcacaacctgagatgacttgggaaaaatgcttcacaacctgagataacttgTACCAACATTGGTATTATTACTGGGACCAAATGTGACTTT | One primer sits in the repeat region |
| BRCA1 | BRCA1_Intron_2__region_1__5 | 17 | 41271534 | 41271710 | ACACTGACGACATGGTTCTACAACTCCTTGGGCTATTGCAAGAAT | TACGGTAGCAGAGACTTGGTCTTCAGAGTTTTCTTTGTCAAGGTTGTT | 177 | 36 | actccttgggctattgcaagaataaaattaaatgctacttgggaaaatgcttcacaacctgagatgacttgggaaaaatgcttcacaacctgagataacttgTACCAACATTGGTATTATTACTGGGACCAAATGTGACTTTAAAAAGAAAAACAACCTTGACAAAGAAAACTCTGA | One primer sits in the repeat region |
| BRCA1 | BRCA1_Intron_2__region_1__6 | 17 | 41271642 | 41271822 | ACACTGACGACATGGTTCTACACATTGGTATTATTACTGGGACCAAATGT | TACGGTAGCAGAGACTTGGTCTAGTCAGTGGTGAGAATGAAAGCC | 181 | 33 | CATTGGTATTATTACTGGGACCAAATGTGACTTTAAAAAGAAAAACAACCTTGACAAAGAAAACTCTGATTGGTTACTAAATCCCTATTTCTGAGATAAGCTACATTTCAAAGAAATTCTCCGTAAAAGAAAAATTGGATTCAGTTATCATACCAGATGGCTTTCATTCTCACCACTGACT |  |
| BRCA1 | BRCA1_Intron_2__region_1__7 | 17 | 41271721 | 41271916 | ACACTGACGACATGGTTCTACAAATCCCTATTTCTGAGATAAGCTACAT | TACGGTAGCAGAGACTTGGTCTTCGGAGTTTTCATCTGTTCAAAGT | 196 | 31 | AATCCCTATTTCTGAGATAAGCTACATTTCAAAGAAATTCTCCGTAAAAGAAAAATTGGATTCAGTTATCATACCAGATGGCTTTCATTCTCACCACTGACTCAATTCTGAAACAATTATATTTCAGTATGGTAATTATAATCTAAACTATATAAACACACTGTAAACACAAACTTTGAACAGATGAAAACTCCGA |  |
| BRCA1 | BRCA1_Intron_2__region_1__8 | 17 | 41271811 | 41272000 | ACACTGACGACATGGTTCTACATCACCACTGACTCAATTCTGAAAC | TACGGTAGCAGAGACTTGGTCTTCAGGACCTAGAAGGGGAACATT | 190 | 31 | TCACCACTGACTCAATTCTGAAACAATTATATTTCAGTATGGTAATTATAATCTAAACTATATAAACACACTGTAAACACAAACTTTGAACAGATGAAAACTCCGATATGTAAAAAGGTAATGAATGTTGAAGGAAGACTGTGAAAAGGGAAAAGAAAAAAAATTAAAATGTTCCCCTTCTAGGTCCTGA |  |
| BRCA1 | BRCA1_Intron_2__region_1__9 | 17 | 41271942 | 41272115 | ACACTGACGACATGGTTCTACAAGGAAGACTGTGAAAAGGGAAAAGAA | TACGGTAGCAGAGACTTGGTCTTCACTTATCCGTATTGGAAGCTCA | 174 | 31 | AGGAAGACTGTGAAAAGGGAAAAGAAAAAAAATTAAAATGTTCCCCTTCTAGGTCCTGATGAGAGTAAATGTTTACTATAAAAATGATTCAAATATTTTAAACACTTTTCAAACCAGGCAATATTTTAGGCCTACTGTATATTTGCATTTTGAGCTTCCAATACGGATAAGTGA |  |
| BRCA1 | BRCA1_Intron_2__region_1__10 | 17 | 41271985 | 41272159 | ACACTGACGACATGGTTCTACACCCTTCTAGGTCCTGATGAGAGTA | TACGGTAGCAGAGACTTGGTCTGGTGGGTTGTTGTTTTTCAACCT | 175 | 35 | CCCTTCTAGGTCCTGATGAGAGTAAATGTTTACTATAAAAATGATTCAAATATTTTAAACACTTTTCAAACCAGGCAATATTTTAGGCCTACTGTATATTTGCATTTTGAGCTTCCAATACGGATAAGTGACTGGAAAAAGCAGCTAGGTTTAGGTTGAAAAACAACAACCCACC | Multiple hits |
| BRCA1 | BRCA1_Intron_2__region_2__1 | 17 | 41275655 | 41275814 | ACACTGACGACATGGTTCTACAGCCCTCGGCCTCATCCAT | TACGGTAGCAGAGACTTGGTCTAATCCTGTGGTCCGGGAAAGAC | 160 | 46 | gccctcggccTCATCCATGATTTTATTTTGCCATTTCAAGTGATGGAGCTTGTTTTAGAGCTGGAAGAAAAGCCAAAATGCCAGTTAATCTAAACTAGATTCCTGCCCCAGTGCAGAACCAATCAAGACAGAGTCCCTGTCTTTCCCGGACCACAGGATT | One primer sits in the repeat region |
| BRCA1 | BRCA1_Intron_2__region_2__3 | 17 | 41275694 | 41275869 | ACACTGACGACATGGTTCTACAGTGATGGAGCTTGTTTTAGAGCTG | TACGGTAGCAGAGACTTGGTCTTGATTCCTTGTTCTCCATCCACT | 176 | 47 | GTGATGGAGCTTGTTTTAGAGCTGGAAGAAAAGCCAAAATGCCAGTTAATCTAAACTAGATTCCTGCCCCAGTGCAGAACCAATCAAGACAGAGTCCCTGTCTTTCCCGGACCACAGGATTTGTGTTGAAAAGGAGAGGAGTGGGAGAGGCAGAGTGGATGGAGAACAAGGAATCA |  |
| BRCA1 | BRCA1_Intron_2__region_2__4 | 17 | 41275796 | 41275973 | ACACTGACGACATGGTTCTACATTTCCCGGACCACAGGATTTG | TACGGTAGCAGAGACTTGGTCTACAGAATTGACCTTACATACTAGGG | 178 | 38 | TTTCCCGGACCACAGGATTTGTGTTGAAAAGGAGAGGAGTGGGAGAGGCAGAGTGGATGGAGAACAAGGAATCATTTTCTATATTTTTAAAGTTCTTCAGTTAAGAAAATCAGCAATTACAATAGCCTAATCTTACTAGACATGTCTTTTCTTCCCTAGTATGTAAGGTCAATTCTGT |  |
| BRCA1 | BRCA1_Intron_2__region_2__5 | 17 | 41275844 | 41276026 | ACACTGACGACATGGTTCTACACAGAGTGGATGGAGAACAAGGAA | TACGGTAGCAGAGACTTGGTCTAGCACAAGAGTGTATTAATTTGGGA | 183 | 33 | CAGAGTGGATGGAGAACAAGGAATCATTTTCTATATTTTTAAAGTTCTTCAGTTAAGAAAATCAGCAATTACAATAGCCTAATCTTACTAGACATGTCTTTTCTTCCCTAGTATGTAAGGTCAATTCTGTTCATTTGCATAGGAGATAATCATAGGAATCCCAAATTAATACACTCTTGTGCT |  |
| BRCA1 | BRCA1_66 | 17 | 41275974 | 41276147 | ACACTGACGACATGGTTCTACATCATTTGCATAGGAGATAATCATAGGAA | TACGGTAGCAGAGACTTGGTCTTCTAATGTGTTAAAGTTCATTGGAACAG | 174 | 35 | TCATTTGCATAGGAGATAATCATAGGAATCCCAAATTAATACACTCTTGTGCTGACTTACCAGATGGGACACTCTAAGATTTTCTGCATAGCATTAATGACATTTTGTACTTCTTCAACGCGAAGAGCAGATAAATCCATTTCTTTCTGTTCCAATGAACTTTAACACATTAGA |  |
| BRCA1 | BRCA1_5_UTR__exon_1B__1 | 17 | 41277107 | 41277305 | ACACTGACGACATGGTTCTACACTTCCCTCGCGACCTACAAAC | TACGGTAGCAGAGACTTGGTCTCCTCTGCTCTGGGTAAAGGTAGT | 199 | 60 | CTTCCCTCGCGACCTACAAACTGCCCCCCTCCCCAGGGTTCACAACGCCTTACGCCTCTCAGGTTCCGCCCCTACCCCCCGTCAAAGAATACCCATCTGTCAGCTTCGGAAATCCACTCTCCCACGCCAGTACCCCAGAGCATCACTTGGGCCCCCTGTCCCTTTCCCGGGACTCTACTACCTTTACCCAGAGCAGAGG |  |
| BRCA1 | BRCA1_prom1AextF_1 | 17 | 41277234 | 41277419 | ACACTGACGACATGGTTCTACACAGTACCCCAGAGCATCACTT | TACGGTAGCAGAGACTTGGTCTACAGATAAATTAAAACTGCGACTGC | 186 | 59 | CAGTACCCCAGAGCATCACTTGGGCCCCCTGTCCCTTTCCCGGGACTCTACTACCTTTACCCAGAGCAGAGGGTGAAGGCCTCCTGAGCGCAGGGGCCCAGTTATCTGAGAAACCCCACAGCCTGTCCCCCGTCCAGGAAGTCTCAGCGAGCTCACGCCGCGCAGTCGCAGTTTTAATTTATCTGT |  |
| BRCA1 | BRCA1_5_UTR__exon_1A__2 | 17 | 41277330 | 41277526 | ACACTGACGACATGGTTCTACACCCAGTTATCTGAGAAACCCCAC | TACGGTAGCAGAGACTTGGTCTCTTTCTGTCCCTCCCATCCTCT | 197 | 54 | CCCAGTTATCTGAGAAACCCCACAGCCTGTCCCCCGTCCAGGAAGTCTCAGCGAGCTCACGCCGCGCAGTCGCAGTTTTAATTTATCTGTAATTCCCGCGCTTTTCCGTTGCCACGGAAACCAAGGGGCTACCGCTAAGCAGCAGCCTCTCAGAATACGAAATCAAGGTACAATCAGAGGATGGGAGGGACAGAAAG |  |
| BRCA1 | BRCA1_prom1AextF_3 | 17 | 41277442 | 41277637 | ACACTGACGACATGGTTCTACACACGGAAACCAAGGGGCTAC | TACGGTAGCAGAGACTTGGTCTAGGCACTTTATGGCAAACTCAGG | 196 | 53 | CACGGAAACCAAGGGGCTACCGCTAAGCAGCAGCCTCTCAGAATACGAAATCAAGGTACAATCAGAGGATGGGAGGGACAGAAAGAGCCAAGCGTCTCTCGGGGCTCTGGATTGGCCACCCAGTCTGCCCCCGGATGACGTAAAAGGAAAGAGACGGAAGAGGAAGAATTCTACCTGAGTTTGCCATAAAGTGCCT |  |
| BRCA1 | BRCA1_prom1AextF_4 | 17 | 41277536 | 41277729 | ACACTGACGACATGGTTCTACATCTCTCGGGGCTCTGGATTG | TACGGTAGCAGAGACTTGGTCTACTGCTTTGGACAATAGGTAGCG | 194 | 51 | TCTCTCGGGGCTCTGGATTGGCCACCCAGTCTGCCCCCGGATGACGTAAAAGGAAAGAGACGGAAGAGGAAGAATTCTACCTGAGTTTGCCATAAAGTGCCTGCCCTCTAGCCTCTACTCTTCCAGTTGCGGCTTATTGCATCACAGTAATTGCTGTACGAAGGTCAGAATCGCTACCTATTGTCCAAAGCAGT |  |
| BRCA1 | BRCA1_Promoter_3 | 17 | 41277639 | 41277818 | ACACTGACGACATGGTTCTACACCCTCTAGCCTCTACTCTTCCAG | TACGGTAGCAGAGACTTGGTCTCCCCCCAACAATCCTTATTACTT | 180 | 48 | CCCTCTAGCCTCTACTCTTCCAGTTGCGGCTTATTGCATCACAGTAATTGCTGTACGAAGGTCAGAATCGCTACCTATTGTCCAAAGCAGTCGTAAGAAGAGGTCCCAATCCCCCACTCTTTCCGCCCTAATGGAGGTCTCCAGTTTCGGTAAATATAAGTAATAAGGATTGTTGGGGGG |  |
| BRCA1 | BRCA1_Promoter_4 | 17 | 41277726 | 41277921 | ACACTGACGACATGGTTCTACACAGTCGTAAGAAGAGGTCCCAAT | TACGGTAGCAGAGACTTGGTCTTGGTATTGGATGTTCCTCTCCAT | 196 | 46 | CAGTCGTAAGAAGAGGTCCCAATCCCCCACTCTTTCCGCCCTAATGGAGGTCTCCAGTTTCGGTAAATATAAGTAATAAGGATTGTTGGGGGGGTGGAGGGAAATAATTATTTCCAGCATGCGTTGCGGAATGAAAGGTCTTCGCCACAGTGTTCCTTAGAAACTGTAGTCTTATGGAGAGGAACATCCAATACCA |  |
| BRCA1 | BRCA1_Promoter_5 | 17 | 41277847 | 41278043 | ACACTGACGACATGGTTCTACACGTTGCGGAATGAAAGGTCTTC | TACGGTAGCAGAGACTTGGTCTAGGCCTAGTTTCTGCTTTCAAAAT | 197 | 46 | CGTTGCGGAATGAAAGGTCTTCGCCACAGTGTTCCTTAGAAACTGTAGTCTTATGGAGAGGAACATCCAATACCAGAGCGGGCACAATTCTCACGGAAATCCAGTGGATAGATTGGAGACCTGTGCGCGCTTGTACTTGTCAACAGTTATGGACTGGAGTGTTATGTTTTCGTATTTTGAAAGCAGAAACTAGGCCT |  |
| BRCA1 | BRCA1_Promoter_6 | 17 | 41277901 | 41278096 | ACACTGACGACATGGTTCTACAGGAGAGGAACATCCAATACCAGAG | TACGGTAGCAGAGACTTGGTCTCTGGGGCTGGATGGGAATTG | 196 | 45 | GGAGAGGAACATCCAATACCAGAGCGGGCACAATTCTCACGGAAATCCAGTGGATAGATTGGAGACCTGTGCGCGCTTGTACTTGTCAACAGTTATGGACTGGAGTGTTATGTTTTCGTATTTTGAAAGCAGAAACTAGGCCTTAAAAAGATACGTACAACTCTTTAGGGAGACTACAATTCCCATCCAGCCCCAG |  |
| BRCA1 | BRCA1_Promoter_7 | 17 | 41278016 | 41278210 | ACACTGACGACATGGTTCTACATCGTATTTTGAAAGCAGAAACTAGGC | TACGGTAGCAGAGACTTGGTCTGAACTACGAGTGCGCAGACA | 195 | 53 | TCGTATTTTGAAAGCAGAAACTAGGCCTTAAAAAGATACGTACAACTCTTTAGGGAGACTACAATTCCCATCCAGCCCCAGGAGTCTGGGGCAAGTAGTCTTGTAAGGTCAGTGGCCTGCGGGGACGCAGTGAGCGCCGAATTTGCCTGGGGCAGGGGAAATGCGCTCTGGCCCATGTCTGCGCACTCGTAGTTC |  |
| BRCA1 | BRCA1_Promoter_8 | 17 | 41278126 | 41278324 | ACACTGACGACATGGTTCTACAAGTGGCCTGCGGGGAC | TACGGTAGCAGAGACTTGGTCTTTACCACTTGTCCCTCAAAACGA | 199 | 59 | AGTGGCCTGCGGGGACGCAGTGAGCGCCGAATTTGCCTGGGGCAGGGGAAATGCGCTCTGGCCCATGTCTGCGCACTCGTAGTTCCACCCCTCAGCCCCAGTGTTTGTTATTTTTCGGGTTCAGCTTGCTTTTGCCCCGTCTCCGTCGACGCAATCGCCACCAGTCAATGGGGTGGTCGTTTTGAGGGACAAGTGGTAA |  |
| BRCA1 | BRCA1_Promoter_9 | 17 | 41278254 | 41278449 | ACACTGACGACATGGTTCTACACTTTTGCCCCGTCTCCGT | TACGGTAGCAGAGACTTGGTCTGAGGCGGCAATGCAAAGAC | 196 | 56 | CTTTTGCCCCGTCTCCGTCGACGCAATCGCCACCAGTCAATGGGGTGGTCGTTTTGAGGGACAAGTGGTAAGAGCCAATCTTCTTGGCGAAAACGCGGAGAAACGGGACTAGTTACTGTCTTTGTCCGCCATGTTAGATTCACCCCACAGAGATAGCGGCAGAGCTGGCAGCGGACGGTCTTTGCATTGCCGCCTC |  |
| BRCA1 | BRCA1_Promoter_10 | 17 | 41278347 | 41278528 | ACACTGACGACATGGTTCTACACGCGGAGAAACGGGACTAGTTA | TACGGTAGCAGAGACTTGGTCTCTCCAAACCCTCTTAGTGTGACG | 182 | 59 | CGCGGAGAAACGGGACTAGTTACTGTCTTTGTCCGCCATGTTAGATTCACCCCACAGAGATAGCGGCAGAGCTGGCAGCGGACGGTCTTTGCATTGCCGCCTCCCCAGGGGGCGGGAAGCTGGTAAGGAAGCAGCCTGGGTTAGCTAGGGGTGGGGTCACGTCACACTAAGAGGGTTTGGAG |  |
| BRCA1_3UTR_Combined | BRCA1_3UTR_Combined_14 | 17 | 41196714 | 41196888 | ACACTGACGACATGGTTCTACATCTTTGGAAACCGGTTCTTG | TACGGTAGCAGAGACTTGGTCTAGATCATACCACGGCACTCC | 175 | 35 | TCTTTGGAAACCGGTTCTTGAAAATCTTCTGCTGTTTTAGAACACATTCTTTAGAAATCTAGCAAATATATCTCAGACTTTTAGAAATCTCTTCTAGTTTCATTTTCCTTTTTTTTTTTTTTTTTTTGAGCCACAGTCTCACTGTCACCCAGGCTGGAGTGCCGTGGTATGATCT |  |
| BRCA1_3UTR_Combined | BRCA1_3UTR_Combined_15 | 17 | 41197039 | 41197205 | ACACTGACGACATGGTTCTACATCGAACTCCTGACCTCCAGT | TACGGTAGCAGAGACTTGGTCTGTCCTTGGGCAGTTCTCAAA | 167 | 51 | TCGAACTCCTGACCTCCAGTGATCTGCCCACCTTGGCCTCCCAAAGTGCTGGGATTACAGGCGTGAGCCACCATGCCCAGGTTTCAAGTTTCCTTTTCATTTCTAATACCTGCCTCAGAATTTCCTCCCCAATGTTCCACTCCAACATTTGAGAACTGCCCAAGGAC |  |
| BRCA1_Intron_2__region_1_ | BRCA1_Intron_2__region_1__11 | 17 | 41271259 | 41271409 | ACACTGACGACATGGTTCTACACAGCCTGGGTGACAGAGAAT | TACGGTAGCAGAGACTTGGTCTTTTCACTGCCCTGTGCTATG | 151 | 38 | CAGCCTGGGTGACAGAGAATCCATCTCAAAAAAAGAAAAAAAAAAAAGAAAAGGATCACAAGAAAAGCTTGTGGACAGTAACCTTATTGTGAAGGGTTGTAATACAACTCTTGTAATCATGGGGTTTTTGACATAGCACAGGGCAGTGAAA |  |
| BRCA1_Intron_2__region_2_ | BRCA1_Intron_2__region_2__7 | 17 | 41275415 | 41275564 | ACACTGACGACATGGTTCTACATGGAGTTCAGTGGTGCCATA | TACGGTAGCAGAGACTTGGTCTTGAAACCCCCTCTCCACTAA | 150 | 50 | TGGAGTTCAGTGGTGCCATATTGGCTCACAGCAACATCTGCCTCCTGGTTCAAGTGATTCTCCTGCCTCAGCCTCCTGAGTAGCTGGGATTACAGGCACATGCCACTACGCCCAGCTAATTTTTGTATTTTTAGTGGAGAGGGGGTTTCA |  |
| BRCA1_Intron_2__region_2_ | BRCA1_Intron_2__region_2__8 | 17 | 41275545 | 41275700 | ACACTGACGACATGGTTCTACATTAGTGGAGAGGGGGTTTCA | TACGGTAGCAGAGACTTGGTCTCCATCACTTGAAATGGCAAA | 156 | 53 | TTAGTGGAGAGGGGGTTTCACCATGTTGGCCAGGATGGTCTCGATCTCCTGACCTCGTGATCCTACCACCTTGGCCTCCCAAAGTGCTGGGATTACAGGCATAAGCCACCGCCCTCGGCCTCATCCATGATTTTATTTTGCCATTTCAAGTGATGG |  |
| BRCA2 | BRCA2_Promoter_1 | 13 | 32888507 | 32888705 | ACACTGACGACATGGTTCTACACACCTTCTGGAAGCAGCAA | TACGGTAGCAGAGACTTGGTCTCATCTTTTGCATTCTTAGGATTCGG | 199 | 47 | CACCTTCTGGAAGCAGCAAGGCCCCCATGGGAGCAACTCTCACTGAATCCATTTGAAGGTTTTGTAGGTCTTACAACAAACCCTATTCAGCCTTGTATTAGGCATGTTACAGAACCAACGAATTCGGAGATGAAGTCAGGTCTTCCAGTTCAGCCTGCGAGGAAGACAGGTGATCCGAATCCTAAGAATGCAAAAGATG |  |
| BRCA2 | BRCA2_Promoter_3 | 13 | 32888996 | 32889147 | ACACTGACGACATGGTTCTACAaaaagcaaaaGATACTACCAAGCC | TACGGTAGCAGAGACTTGGTCTTTTAGTTCCAGTAGCTGTTCTGC | 152 | 46 | aaaagcaaaaGATACTACCAAGCCCTGCGGAGCAAGGTACCTCACACTTCATGAGCGAGTTAAGATGGGTTTCACAATTTTTCAAGCAAGGAAACGGGCTCGGAGGTCTTGAACACCTGCTACCCAATAGCAGAACAGCTACTGGAACTAAA | One primer sits in the repeat region |
| BRCA2 | BRCA2_Promoter_6 | 13 | 32889044 | 32889230 | ACACTGACGACATGGTTCTACATCATGAGCGAGTTAAGATGGGTT | TACGGTAGCAGAGACTTGGTCTAAAAGCTTAGAGTGGTCGGTGTG | 187 | 46 | TCATGAGCGAGTTAAGATGGGTTTCACAATTTTTCAAGCAAGGAAACGGGCTCGGAGGTCTTGAACACCTGCTACCCAATAGCAGAACAGCTACTGGAACTAAAATCCTCTGATTTCAAATAACAGCCCCGCCCACTACCACTAAGTGAAGTCATCCACAACCACACACCGACCACTCTAAGCTTTT |  |
| BRCA2 | BRCA2_prom_Stacey_1 | 13 | 32889175 | 32889357 | ACACTGACGACATGGTTCTACACCCACTACCACTAAGTGAAGTCAT | TACGGTAGCAGAGACTTGGTCTTTTTTGCTCCAGCTCATGTTTGG | 183 | 50 | CCCACTACCACTAAGTGAAGTCATCCACAACCACACACCGACCACTCTAAGCTTTTGTAAGATCGGCTCGCTTTGGGGAACAGGTCTTGAGAGAACATCCCTTTTAAGGTCAGAACAAAGGTATTTCATAGGTCCCAGGTCGTGTCCCGAGGGCGCCCACCCAAACATGAGCTGGAGCAAAAA |  |
| BRCA2 | BRCA2_prom_Stacey_2 | 13 | 32889210 | 32889390 | ACACTGACGACATGGTTCTACACACCGACCACTCTAAGCTTTTGT | TACGGTAGCAGAGACTTGGTCTCCCTATGCCTACTCCAAGTCCC | 181 | 52 | CACCGACCACTCTAAGCTTTTGTAAGATCGGCTCGCTTTGGGGAACAGGTCTTGAGAGAACATCCCTTTTAAGGTCAGAACAAAGGTATTTCATAGGTCCCAGGTCGTGTCCCGAGGGCGCCCACCCAAACATGAGCTGGAGCAAAAAGAAAGGGATGGGGGACTTGGAGTAGGCATAGGG |  |
| BRCA2 | BRCA2_Promoter_Combined_7 | 13 | 32889335 | 32889530 | ACACTGACGACATGGTTCTACACCAAACATGAGCTGGAGCAAAAA | TACGGTAGCAGAGACTTGGTCTATTTCTCAGTGTGGCGAAAGGAA | 196 | 54 | CCAAACATGAGCTGGAGCAAAAAGAAAGGGATGGGGGACTTGGAGTAGGCATAGGGGCGGCCCCTCCAAGCAGGGTGGCCTGGGACTCTTAAGGGTCAGCGAGAAGAGAACACACACTCCAGCTCCCGCTTTATTCGGTCAGATACTGACGGTTGGGATGCCTGACAAGGAATTTCCTTTCGCCACACTGAGAAAT |  |
| BRCA2 | BRCA2_Promoter_10 | 13 | 32889458 | 32889650 | ACACTGACGACATGGTTCTACATCCCGCTTTATTCGGTCAGATAC | TACGGTAGCAGAGACTTGGTCTGCCTCTGCCGCCTAGTT | 193 | 62 | TCCCGCTTTATTCGGTCAGATACTGACGGTTGGGATGCCTGACAAGGAATTTCCTTTCGCCACACTGAGAAATACCCGCAGCGGCCCACCCAGGCCTGACTTCCGGGTGGTGCGTGTGCTGCGTGTCGCGTCACGGCGTCACGTGGCCAGCGCGGGCTTGTGGCGCGAGCTTCTGAAACTAGGCGGCAGAGGC |  |
| BRCA2 | BRCA2_Promoter_Combined_10 | 13 | 32889508 | 32889703 | ACACTGACGACATGGTTCTACATTCCTTTCGCCACACTGAGAAAT | TACGGTAGCAGAGACTTGGTCTCCGCAAAAGACACCCGAGG | 196 | 67 | TTCCTTTCGCCACACTGAGAAATACCCGCAGCGGCCCACCCAGGCCTGACTTCCGGGTGGTGCGTGTGCTGCGTGTCGCGTCACGGCGTCACGTGGCCAGCGCGGGCTTGTGGCGCGAGCTTCTGAAACTAGGCGGCAGAGGCGGAGCCGCTGTGGCACTGCTGCGCCTCTGCTGCGCCTCGGGTGTCTTTTGCGG |  |
| BRCA2 | BRCA2_5_UTR__exon_1__2 | 13 | 32889663 | 32889835 | ACACTGACGACATGGTTCTACAGCACTGCTGCGCCTCT | TACGGTAGCAGAGACTTGGTCTCTCGTCCCAACCCACTACCA | 173 | 62 | GCACTGCTGCGCCTCTGCTGCGCCTCGGGTGTCTTTTGCGGCGGTGGGTCGCCGCCGGGAGAAGCGTGAGGGGACAGATTTGTGACCGGCGCGGTTTTTGTCAGCTTACTCCGGCCAAAAAAGAACTGCACCTCTGGAGCGGGTTAGTGGTGGTGGTAGTGGGTTGGGACGAG |  |
| BRCA2 | BRCA2_prom_Stacey_7 | 13 | 32889723 | 32889921 | ACACTGACGACATGGTTCTACAGAAGCGTGAGGGGACAGATTT | TACGGTAGCAGAGACTTGGTCTAGAGACAAAAGGGCAAGAAGCC | 199 | 63 | GAAGCGTGAGGGGACAGATTTGTGACCGGCGCGGTTTTTGTCAGCTTACTCCGGCCAAAAAAGAACTGCACCTCTGGAGCGGGTTAGTGGTGGTGGTAGTGGGTTGGGACGAGCGCGTCTTCCGCAGTCCCAGTCCAGCGTGGCGGGGGAGCGCCTCACGCCCCGGGTCGCTGCCGCGGCTTCTTGCCCTTTTGTCTCT |  |
| BRCA2 | BRCA2_prom_Stacey_8 | 13 | 32889839 | 32890018 | ACACTGACGACATGGTTCTACAGTCTTCCGCAGTCCCAGTC | TACGGTAGCAGAGACTTGGTCTGAAATGGAGACCCAGGGAAGG | 180 | 64 | GTCTTCCGCAGTCCCAGTCCAGCGTGGCGGGGGAGCGCCTCACGCCCCGGGTCGCTGCCGCGGCTTCTTGCCCTTTTGTCTCTGCCAACCCCCACCCATGCCTGAGAGAAAGGTCCTTGCCCGAAGGCAGATTTTCGCCAAGCAAATTCGAGCCCCGCCCCTTCCCTGGGTCTCCATTTC |  |
| BRCA2 | BRCA2_1 | 13 | 32890522 | 32890720 | ACACTGACGACATGGTTCTACATCCCTGTGTAAGTGCATTTTGGT | TACGGTAGCAGAGACTTGGTCTTTTTAGAAAACACTTTCTCGGTGTAAT | 199 | 34 | TCCCTGTGTAAGTGCATTTTGGTCTTCTGTTTTGCAGACTTATTTACCAAGCATTGGAGGAATATCGTAGGTAAAAATGCCTATTGGATCCAAAGAGAGGCCAACATTTTTTGAAATTTTTAAGACACGCTGCAACAAAGCAGGTATTGACAAATTTTATATAACTTTATAAATTACACCGAGAAAGTGTTTTCTAAAA |  |
| BRCA2 | BRCA2_2 | 13 | 32893174 | 32893358 | ACACTGACGACATGGTTCTACATCACTGGTTAAAACTAAGGTGGGA | TACGGTAGCAGAGACTTGGTCTTAAGATGGTTTCCTTTGTGGAGT | 185 | 32 | TCACTGGTTAAAACTAAGGTGGGATTTTTTTTTTAAATAGATTTAGGACCAATAAGTCTTAATTGGTTTGAAGAACTTTCTTCAGAAGCTCCACCCTATAATTCTGAACCTGCAGAAGAATCTGAACATAAAAACAACAATTACGAACCAAACCTATTTAAAACTCCACAAAGGAAACCATCTTA |  |
| BRCA2 | BRCA2_3 | 13 | 32893249 | 32893426 | ACACTGACGACATGGTTCTACACTTTCTTCAGAAGCTCCACCCTA | TACGGTAGCAGAGACTTGGTCTGAGATTGGTACAGCGGCAGAG | 178 | 39 | CTTTCTTCAGAAGCTCCACCCTATAATTCTGAACCTGCAGAAGAATCTGAACATAAAAACAACAATTACGAACCAAACCTATTTAAAACTCCACAAAGGAAACCATCTTATAATCAGCTGGCTTCAACTCCAATAATATTCAAAGAGCAAGGGCTGACTCTGCCGCTGTACCAATCTC | Assays designed by relax mode and have no off-target hits |
| BRCA2 | BRCA2_4 | 13 | 32893336 | 32893492 | ACACTGACGACATGGTTCTACAACTCCACAAAGGAAACCATCTTA | TACGGTAGCAGAGACTTGGTCTCTCCCCAGTCTACCATATTGCAT | 157 | 39 | ACTCCACAAAGGAAACCATCTTATAATCAGCTGGCTTCAACTCCAATAATATTCAAAGAGCAAGGGCTGACTCTGCCGCTGTACCAATCTCCTGTAAAAGAATTAGATAAATTCAAATTAGACTTAGGTAAGTAATgcaatatggtagactggggag | One primer sits in the repeat region |
| BRCA2 | BRCA2_Intron_3_1 | 13 | 32897972 | 32898163 | ACACTGACGACATGGTTCTACAACTTAATGCCCTGGAGAGTCAAA | TACGGTAGCAGAGACTTGGTCTAGACAAAGTGAGTTGCAGACAGTA | 192 | 29 | ACTTAATGCCCTGGAGAGTCAAATATAATCTACTCTAATACAGAAAATAGAAATATTGAAAAACTGTAAATTGGATTTCATATTGTTAAAGCCACCTATAGCTTTAGAAACTCTGAACATTATTTTCTTAGAAAATGGATGTGTTCAATAAGAATAGAAATTATGTATTACTGTCTGCAACTCACTTTGTCT |  |
| BRCA2 | BRCA2_Intron_3_2 | 13 | 32898044 | 32898221 | ACACTGACGACATGGTTCTACAGGATTTCATATTGTTAAAGCCACCTAT | TACGGTAGCAGAGACTTGGTCTTGTCTGGTATATTAGTCACTCCTGAA | 178 | 30 | GGATTTCATATTGTTAAAGCCACCTATAGCTTTAGAAACTCTGAACATTATTTTCTTAGAAAATGGATGTGTTCAATAAGAATAGAAATTATGTATTACTGTCTGCAACTCACTTTGTCTAATTATATCCAatttattcatccagtcaatatttcaggagtgactaatataccagaca | One primer sits in the repeat region |
| BRCA2 | BRCA2_Intron_3_3 | 13 | 32898141 | 32898311 | ACACTGACGACATGGTTCTACAACTGTCTGCAACTCACTTTGTCT | TACGGTAGCAGAGACTTGGTCTACACTGTTCTTTTTCCCCCTACT | 171 | 37 | ACTGTCTGCAACTCACTTTGTCTAATTATATCCAatttattcatccagtcaatatttcaggagtgactaatataccagacatttttgtagttgctagggatacagtgacaaataagacaaaatctctacctcagattgctcacagcctagtagggggaaaaagaacagtgt | One primer sits in the repeat region |
| BRCA2 | BRCA2_Intron_3_4 | 13 | 32898184 | 32898355 | ACACTGACGACATGGTTCTACATCCAGTCAATATTTCAGGAGTGACTAA | TACGGTAGCAGAGACTTGGTCTTTAAGTGTTTGCCCCCCTATGTG | 172 | 40 | tccagtcaatatttcaggagtgactaatataccagacatttttgtagttgctagggatacagtgacaaataagacaaaatctctacctcagattgctcacagcctagtagggggaaaaagaacagtgtatgatcaaactcttcagggaacacataggggggcaaacacttaa | Two primers sits in the repeat region |
| BRCA2 | BRCA2_Intron_3_5 | 13 | 32898267 | 32898457 | ACACTGACGACATGGTTCTACATACCTCAGATTGCTCACAGCCTA | TACGGTAGCAGAGACTTGGTCTGCTGTTTCCCCTTCTTTGCTTTT | 191 | 44 | tacctcagattgctcacagcctagtagggggaaaaagaacagtgtatgatcaaactcttcagggaacacataggggggcaaacacttaatcttaccttagggatcactacagttttctggaggaggtagtttctaaatggaagcctgaaagagttgttccaggtcaagaaaagcaaagaaggggaaacagc | Two primers sits in the repeat region |
| BRCA2 | BRCA2_Intron_3_6 | 13 | 32898366 | 32898559 | ACACTGACGACATGGTTCTACAGGGATCACTACAGTTTTCTGGAGG | TACGGTAGCAGAGACTTGGTCTAATTTTTATGATGCCATTCTTTTACAGTC | 194 | 38 | gggatcactacagttttctggaggaggtagtttctaaatggaagcctgaaagagttgttccaggtcaagaaaagcaaagaaggggaaacagcttgtacaaagtcctagaggttaaagaaaacattctttcaggatatgcaaatggttgggtatgggtaaaaagtagactgtaaaagaatggCATCATAAAAATT | Two primers sits in the repeat region |
| BRCA2 | BRCA2_Intron_3_7 | 13 | 32898426 | 32898622 | ACACTGACGACATGGTTCTACACAGGTCAAGAAAAGCAAAGAAGGG | TACGGTAGCAGAGACTTGGTCTTTTCTTCATTTTTAATTTTTGTGGGTACAT | 197 | 30 | caggtcaagaaaagcaaagaaggggaaacagcttgtacaaagtcctagaggttaaagaaaacattctttcaggatatgcaaatggttgggtatgggtaaaaagtagactgtaaaagaatggCATCATAAAAATTAAGTAAATTGTCAcataaatatatatatttcttatgtacccacaaaaattaaaaaTGAAGAAA | Two primers sits in the repeat region |
| BRCA2 | BRCA2_Intron_3_8 | 13 | 32898527 | 32898716 | ACACTGACGACATGGTTCTACAAGTAGACTGTAAAAGAATGGCATCA | TACGGTAGCAGAGACTTGGTCTTGACAATATCACTTGTGCTAAAAACCT | 190 | 27 | agtagactgtaaaagaatggCATCATAAAAATTAAGTAAATTGTCAcataaatatatatatttcttatgtacccacaaaaattaaaaaTGAAGAAATTAAGTAAATTGTGAAAGGCCTTCATACTATGGAGTTTGACTTGATCTTGAAAAGTAAGATCTTGAAAGGTTTTTAGCACAAGTGATATTGTCA | One primer sits in the repeat region |
| BRCA2 | BRCA2_5 | 13 | 32899149 | 32899334 | ACACTGACGACATGGTTCTACACCAAAGAATGCAAATTTATAATCCAGAGT | TACGGTAGCAGAGACTTGGTCTAGCTTCATCATACCTTTCACTAAGAC | 186 | 34 | ccaaagaatgcaaatttataatcCAGAGTATATACATTCTCACTGAATTATTGTACTGTTTCAGGAAGGAATGTTCCCAATAGTAGACATAAAAGTCTTCGCACAGTGAAAACTAAAATGGATCAAGCAGATGATGTTTCCTGTCCACTTCTAAATTCTTGTCTTAGTGAAAGGTATGATGAAGCt | Assays designed by relax mode and have no off-target hits |
| BRCA2 | BRCA2_6 | 13 | 32899280 | 32899435 | ACACTGACGACATGGTTCTACATGATGTTTCCTGTCCACTTCTAAA | TACGGTAGCAGAGACTTGGTCTTCTACCAGGCTCTTAGCCAAAAT | 156 | 28 | TGATGTTTCCTGTCCACTTCTAAATTCTTGTCTTAGTGAAAGGTATGATGAAGCtattatattaaaatatttaaatGAAACATTTTCCTACATATATTTGTTCTATAAAGATGAATCTGATTTTTATGCTAATATTTTGGCTAAGAGCCTGGTAGA |  |
| BRCA2 | BRCA2_7 | 13 | 32900202 | 32900400 | ACACTGACGACATGGTTCTACAAAAATAACCTAAGGGATTTGCTTTGT | TACGGTAGCAGAGACTTGGTCTTGAAACAAACTCCCACATACCACT | 199 | 30 | AAAATAACCTAAGGGATTTGCTTTGTTTTATTTTAGTCCTGTTGTTCTACAATGTACACATGTAACACCACAAAGAGATAAGTCAGGTATGATTAAAAACAATGCTTTTTATTCTTAGAATACTAGAAATGTTAATAAAAATAAAACTTAACAATTTTCCCCTTTTTTTACCCCCAGTGGTATGTGGGAGTTTGTTTCA | Assays designed by relax mode and have no off-target hits |
| BRCA2 | BRCA2_8 | 13 | 32900331 | 32900527 | ACACTGACGACATGGTTCTACATGTTAATAAAAATAAAACTTAACAATTTTCCCCTT | TACGGTAGCAGAGACTTGGTCTTGAGGCAGAATGCTAGGTACAGA | 197 | 33 | TGTTAATAAAAATAAAACTTAACAATTTTCCCCTTTTTTTACCCCCAGTGGTATGTGGGAGTTTGTTTCATACACCAAAGTTTGTGAAGGTAAATATTCTACCTGGTTTATTTTTATGACTTAGTAATTGAGAATTTGACAATAGCGTTATACCTTTGCCCTGAGATTTACAAATCTGTACCTAGCATTCTGCCTCA |  |
| BRCA2 | BRCA2_9 | 13 | 32900545 | 32900728 | ACACTGACGACATGGTTCTACAACGTTAAGTGAAATAAAGAGTGAATGAA | TACGGTAGCAGAGACTTGGTCTGGTGGGTGGTGTAGCTAAAGA | 184 | 37 | ACGTTAAGTGAAATAAAGAGTGAATGAAAAAATAATATCCTTAATGATCAGGGCATTTCTATAAAAAATAAACTATTTTCTTTCCTCCCAGGGTCGTCAGACACCAAAACATATTTCTGAAAGTCTAGGAGCTGAGGTGGATCCTGATATGTCTTGGTCAAGTTCTTTAGCTACACCACCCACC |  |
| BRCA2 | BRCA2_10 | 13 | 32900618 | 32900797 | ACACTGACGACATGGTTCTACATATTTTCTTTCCTCCCAGGGTCGT | TACGGTAGCAGAGACTTGGTCTTCAACCTCATCTGCTCTTTCTTG | 180 | 41 | TATTTTCTTTCCTCCCAGGGTCGTCAGACACCAAAACATATTTCTGAAAGTCTAGGAGCTGAGGTGGATCCTGATATGTCTTGGTCAAGTTCTTTAGCTACACCACCCACCCTTAGTTCTACTGTGCTCATAGGTAATAATAGCAAATGTGTATTTACAAGAAAGAGCAGATGAGGTTGA | Assays designed by relax mode and have no off-target hits |
| BRCA2 | BRCA2_11 | 13 | 32903475 | 32903668 | ACACTGACGACATGGTTCTACATGTGTCATGTAATCAAATAGTAGATGTG | TACGGTAGCAGAGACTTGGTCTAGCAATTTCAACAGTCTAATCAATGTC | 194 | 29 | TGTGTCATGTAATCAAATAGTAGATGTGCTTTTTGATGTCTGACAAAAAATAAGTTTTTGCATTCTAGTGATAATATACAATACACATAAATTTTTATCTTACAGTCAGAAATGAAGAAGCATCTGAAACTGTATTTCCTCATGATACTACTGCTGTAAGTAAATATGACATTGATTAGACTGTTGAAATTGCT |  |
| BRCA2 | BRCA2_Intron_8_1 | 13 | 32904422 | 32904620 | ACACTGACGACATGGTTCTACATGTGACAGCAAAACCAGCTCATA | TACGGTAGCAGAGACTTGGTCTGGCCCCAAAGCACAAGTATAATG | 199 | 38 | tgtgacagcaaaaccagctcatatgtctttctccttcacaatctcacagatagatttgttcttaccatagatgtcgcagtacaatttttttcctttccttaagtcgagaactttcactgtttcaattaaaggaagcactttatggcttctttttggcatatttgaattgccagcatcattatacttgtgctttggggcc | Two primers sits in the repeat region |
| BRCA2 | BRCA2_Intron_8_2 | 13 | 32904478 | 32904657 | ACACTGACGACATGGTTCTACATGTTCTTACCATAGATGTCGCAGT | TACGGTAGCAGAGACTTGGTCTTGCTTGTGTTCAAGTCACCCTTA | 180 | 36 | tgttcttaccatagatgtcgcagtacaatttttttcctttccttaagtcgagaactttcactgtttcaattaaaggaagcactttatggcttctttttggcatatttgaattgccagcatcattatacttgtgctttggggccattgttaagtaaaataagggtgacttgaacacaagca | Two primers sits in the repeat region |
| BRCA2 | BRCA2_Intron_8_3 | 13 | 32904600 | 32904770 | ACACTGACGACATGGTTCTACATTATACTTGTGCTTTGGGGCCAT | TACGGTAGCAGAGACTTGGTCTACTTGGGACATGAATCATCCCTT | 171 | 43 | ttatacttgtgctttggggccattgttaagtaaaataagggtgacttgaacacaagcactgtggtaccacaatagccgatctgataaccaagacaactactaagtgactaataggtgggtaccatatacagcctggatacgctggacaaagggatgattcatgtcccaagt | Two primers sits in the repeat region |
| BRCA2 | BRCA2_Intron_8_4 | 13 | 32904673 | 32904852 | ACACTGACGACATGGTTCTACAAGCCGATCTGATAACCAAGACAA | TACGGTAGCAGAGACTTGGTCTCTTGCACCACTAGGATGTGGAAA | 180 | 43 | agccgatctgataaccaagacaactactaagtgactaataggtgggtaccatatacagcctggatacgctggacaaagggatgattcatgtcccaagtgggatggagcaagatggtgcaagtttttttttctccatttccattttcctttcctaagatttccacatcctagtggtgcaag | Two primers sits in the repeat region |
| BRCA2 | BRCA2_Intron_8_5 | 13 | 32904749 | 32904920 | ACACTGACGACATGGTTCTACAAGGGATGATTCATGTCCCAAGTG | TACGGTAGCAGAGACTTGGTCTGGAAAATTCCAGAAGTAAGCAATTAGT | 172 | 38 | agggatgattcatgtcccaagtgggatggagcaagatggtgcaagtttttttttctccatttccattttcctttcctaagatttccacatcctagtggtgcaagatttcatcacactactcaggatgacacacaatttaaaacttactaattgcttacttctggaattttcc | Two primers sits in the repeat region |
| BRCA2 | BRCA2_Intron_8_6 | 13 | 32904844 | 32905015 | ACACTGACGACATGGTTCTACATGGTGCAAGATTTCATCACACTA | TACGGTAGCAGAGACTTGGTCTTCAATGCACATATAGTAGTAGTCCC | 172 | 35 | tggtgcaagatttcatcacactactcaggatgacacacaatttaaaacttactaattgcttacttctggaattttccattaaaaatttttggacctaggttgattgcagataactgaaatcaccaaaagtgaaaccatggataaggggggactactACTATATGTGCATTGA | Two primers sits in the repeat region |
| BRCA2 | BRCA2_Intron_8_7 | 13 | 32904887 | 32905077 | ACACTGACGACATGGTTCTACAAAAACTTACTAATTGCTTACTTCTGGAAT | TACGGTAGCAGAGACTTGGTCTTGGAAAAATAGCTTTTCACATTCTGC | 191 | 31 | aaaacttactaattgcttacttctggaattttccattaaaaatttttggacctaggttgattgcagataactgaaatcaccaaaagtgaaaccatggataaggggggactactACTATATGTGCATTGAGAGTTTTTATACTAGTGATTTTAAACTATAATTTTTGCAGAATGTGAAAAGCTATTTTTCCA | Assays designed by relax mode and have no off-target hits |
| BRCA2 | BRCA2_12 | 13 | 32904990 | 32905188 | ACACTGACGACATGGTTCTACAGGGGACTACTACTATATGTGCATTGA | TACGGTAGCAGAGACTTGGTCTTCAACTAAACAGAGGACTTACCATGA | 199 | 33 | ggggactactACTATATGTGCATTGAGAGTTTTTATACTAGTGATTTTAAACTATAATTTTTGCAGAATGTGAAAAGCTATTTTTCCAATCATGATGAAAGTCTGAAGAAAAATGATAGATTTATCGCTTCTGTGACAGACAGTGAAAACACAAATCAAAGAGAAGCTGCAAGTCATGGTAAGTCCTCTGTTTAGTTGA | Assays designed by relax mode and have no off-target hits |
| BRCA2 | BRCA2_126 | 13 | 32905120 | 32905279 | ACACTGACGACATGGTTCTACACTGTGACAGACAGTGAAAACACA | TACGGTAGCAGAGACTTGGTCTAGGTTGCGGTAAACCGAGAT | 160 | 40 | CTGTGACAGACAGTGAAAACACAAATCAAAGAGAAGCTGCAAGTCATGGTAAGTCCTCTGTTTAGTTGAACTACAGGTTTTTTTGTTGTTGTTGTTTTGATTTTTTTTTTTTGAGGTGGAGTCTTGCTCTGTCACCCGTGATCTCGGTTTACCGCAACCT |  |
| BRCA2 | BRCA2_13 | 13 | 32906365 | 32906537 | ACACTGACGACATGGTTCTACATGGCTTATAAAATATTAATGTGCTTCTGT | TACGGTAGCAGAGACTTGGTCTAACTATCTTCTTCAGAGGTATCTACAAC | 173 | 32 | TGGCTTATAAAATATTAATGTGCTTCTGTTTTATACTTTAACAGGATTTGGAAAAACATCAGGGAATTCATTTAAAGTAAATAGCTGCAAAGACCACATTGGAAAGTCAATGCCAAATGTCCTAGAAGATGAAGTATATGAAACAGTTGTAGATACCTCTGAAGAAGATAGTT | Assays designed by relax mode and have no off-target hits |
| BRCA2 | BRCA2_14 | 13 | 32906405 | 32906593 | ACACTGACGACATGGTTCTACAACAGGATTTGGAAAAACATCAGGG | TACGGTAGCAGAGACTTGGTCTGCTAGTTCTTACTTTTTGTAGATTTTTTGTTCT | 189 | 31 | ACAGGATTTGGAAAAACATCAGGGAATTCATTTAAAGTAAATAGCTGCAAAGACCACATTGGAAAGTCAATGCCAAATGTCCTAGAAGATGAAGTATATGAAACAGTTGTAGATACCTCTGAAGAAGATAGTTTTTCATTATGTTTTTCTAAATGTAGAACAAAAAATCTACAAAAAGTAAGAACTAGC | Assays designed by relax mode and have no off-target hits |
| BRCA2 | BRCA2_15 | 13 | 32906502 | 32906695 | ACACTGACGACATGGTTCTACAATGAAACAGTTGTAGATACCTCTGAA | TACGGTAGCAGAGACTTGGTCTTGGTTCCACTTCAGATACAAATGAGT | 194 | 29 | ATGAAACAGTTGTAGATACCTCTGAAGAAGATAGTTTTTCATTATGTTTTTCTAAATGTAGAACAAAAAATCTACAAAAAGTAAGAACTAGCAAGACTAGGAAAAAAATTTTCCATGAAGCAAACGCTGATGAATGTGAAAAATCTAAAAACCAAGTGAAAGAAAAATACTCATTTGTATCTGAAGTGGAACCA | Assays designed by relax mode and have no off-target hits |
| BRCA2 | BRCA2_16 | 13 | 32906614 | 32906786 | ACACTGACGACATGGTTCTACACCATGAAGCAAACGCTGATGAAT | TACGGTAGCAGAGACTTGGTCTAAGACGGTACAACTTCCTTGGAG | 173 | 36 | CCATGAAGCAAACGCTGATGAATGTGAAAAATCTAAAAACCAAGTGAAAGAAAAATACTCATTTGTATCTGAAGTGGAACCAAATGATACTGATCCATTAGATTCAAATGTAGCAAATCAGAAGCCCTTTGAGAGTGGAAGTGACAAAATCTCCAAGGAAGTTGTACCGTCTT | Assays designed by relax mode and have no off-target hits |
| BRCA2 | BRCA2_17 | 13 | 32906677 | 32906859 | ACACTGACGACATGGTTCTACATGTATCTGAAGTGGAACCAAATGA | TACGGTAGCAGAGACTTGGTCTTGCAATAGGGGTATTTTCTCCAT | 183 | 42 | TGTATCTGAAGTGGAACCAAATGATACTGATCCATTAGATTCAAATGTAGCAAATCAGAAGCCCTTTGAGAGTGGAAGTGACAAAATCTCCAAGGAAGTTGTACCGTCTTTGGCCTGTGAATGGTCTCAACTAACCCTTTCAGGTCTAAATGGAGCCCAGATGGAGAAAATACCCCTATTGCA | Assays designed by relax mode and have no off-target hits |
| BRCA2 | BRCA2_18 | 13 | 32906777 | 32906973 | ACACTGACGACATGGTTCTACAGTACCGTCTTTGGCCTGTGAATG | TACGGTAGCAGAGACTTGGTCTGGTAGGCTAGAAATACGTGGCAAA | 197 | 39 | GTACCGTCTTTGGCCTGTGAATGGTCTCAACTAACCCTTTCAGGTCTAAATGGAGCCCAGATGGAGAAAATACCCCTATTGCATATTTCTTCATGTGACCAAAATATTTCAGAAAAAGACCTATTAGACACAGAGAACAAAAGAAAGAAAGATTTTCTTACTTCAGAGAATTCTTTGCCACGTATTTCTAGCCTACC | Assays designed by relax mode and have no off-target hits |
| BRCA2 | BRCA2_19 | 13 | 32906851 | 32907049 | ACACTGACGACATGGTTCTACACCTATTGCATATTTCTTCATGTGACC | TACGGTAGCAGAGACTTGGTCTTGTATGAGATTCAAGATGCTGCT | 199 | 34 | CCTATTGCATATTTCTTCATGTGACCAAAATATTTCAGAAAAAGACCTATTAGACACAGAGAACAAAAGAAAGAAAGATTTTCTTACTTCAGAGAATTCTTTGCCACGTATTTCTAGCCTACCAAAATCAGAGAAGCCATTAAATGAGGAAACAGTGGTAAATAAGAGAGATGAAGAGCAGCATCTTGAATCTCATACA | Assays designed by relax mode and have no off-target hits |
| BRCA2 | BRCA2_20 | 13 | 32906946 | 32907116 | ACACTGACGACATGGTTCTACAATTCTTTGCCACGTATTTCTAGCC | TACGGTAGCAGAGACTTGGTCTTACCCTGAAATGAAGAAGCCACT | 171 | 40 | ATTCTTTGCCACGTATTTCTAGCCTACCAAAATCAGAGAAGCCATTAAATGAGGAAACAGTGGTAAATAAGAGAGATGAAGAGCAGCATCTTGAATCTCATACAGACTGCATTCTTGCAGTAAAGCAGGCAATATCTGGAACTTCTCCAGTGGCTTCTTCATTTCAGGGTA | Assays designed by relax mode and have no off-target hits |
| BRCA2 | BRCA2_21 | 13 | 32907057 | 32907233 | ACACTGACGACATGGTTCTACATTCTTGCAGTAAAGCAGGCAATA | TACGGTAGCAGAGACTTGGTCTGTCCACTTTCAGAGGCTTCAGTT | 177 | 37 | TTCTTGCAGTAAAGCAGGCAATATCTGGAACTTCTCCAGTGGCTTCTTCATTTCAGGGTATCAAAAAGTCTATATTCAGAATAAGAGAATCACCTAAAGAGACTTTCAATGCAAGTTTTTCAGGTCATATGACTGATCCAAACTTTAAAAAAGAAACTGAAGCCTCTGAAAGTGGAC | Assays designed by relax mode and have no off-target hits |
| BRCA2 | BRCA2_22 | 13 | 32907091 | 32907270 | ACACTGACGACATGGTTCTACATCCAGTGGCTTCTTCATTTCAGG | TACGGTAGCAGAGACTTGGTCTGAGTCCTCCTTCTGTGAGCAAAC | 180 | 37 | TCCAGTGGCTTCTTCATTTCAGGGTATCAAAAAGTCTATATTCAGAATAAGAGAATCACCTAAAGAGACTTTCAATGCAAGTTTTTCAGGTCATATGACTGATCCAAACTTTAAAAAAGAAACTGAAGCCTCTGAAAGTGGACTGGAAATACATACTGTTTGCTCACAGAAGGAGGACTC | Assays designed by relax mode and have no off-target hits |
| BRCA2 | BRCA2_23 | 13 | 32907186 | 32907361 | ACACTGACGACATGGTTCTACATGACTGATCCAAACTTTAAAAAAGAAACT | TACGGTAGCAGAGACTTGGTCTAGTGGATATTAAACCTGCATTCTTCA | 176 | 40 | TGACTGATCCAAACTTTAAAAAAGAAACTGAAGCCTCTGAAAGTGGACTGGAAATACATACTGTTTGCTCACAGAAGGAGGACTCCTTATGTCCAAATTTAATTGATAATGGAAGCTGGCCAGCCACCACCACACAGAATTCTGTAGCTTTGAAGAATGCAGGTTTAATATCCACT | Assays designed by relax mode and have no off-target hits |
| BRCA2 | BRCA2_24 | 13 | 32907264 | 32907455 | ACACTGACGACATGGTTCTACAAGGACTCCTTATGTCCAAATTTAATTGAT | TACGGTAGCAGAGACTTGGTCTTTAGTTCTGATTTTTGGTCTTTCGG | 192 | 32 | AGGACTCCTTATGTCCAAATTTAATTGATAATGGAAGCTGGCCAGCCACCACCACACAGAATTCTGTAGCTTTGAAGAATGCAGGTTTAATATCCACTTTGAAAAAGAAAACAAATAAGTTTATTTATGCTATACATGATGAAACATCTTATAAAGGAAAAAAAATACCGAAAGACCAAAAATCAGAACTAA | Assays designed by relax mode and have no off-target hits |
| BRCA2 | BRCA2_25 | 13 | 32907328 | 32907512 | ACACTGACGACATGGTTCTACATGTAGCTTTGAAGAATGCAGGTTT | TACGGTAGCAGAGACTTGGTCTTTGCAAATGTAAGTGGTGCTTCA | 185 | 30 | TGTAGCTTTGAAGAATGCAGGTTTAATATCCACTTTGAAAAAGAAAACAAATAAGTTTATTTATGCTATACATGATGAAACATCTTATAAAGGAAAAAAAATACCGAAAGACCAAAAATCAGAACTAATTAACTGTTCAGCCCAGTTTGAAGCAAATGCTTTTGAAGCACCACTTACATTTGCAA | Assays designed by relax mode and have no off-target hits |
| BRCA2 | BRCA2_26 | 13 | 32907463 | 32907643 | ACACTGACGACATGGTTCTACATTCAGCCCAGTTTGAAGCA | TACGGTAGCAGAGACTTGGTCTGATGATGCCTAAGATTAAATATAAGATATGAAGA | 181 | 31 | TTCAGCCCAGTTTGAAGCAAATGCTTTTGAAGCACCACTTACATTTGCAAATGCTGATTCAGGTACCTCTGTCTTTTTTTTTTTGTAAATAGTACATATAGTTTTATAGATGACGATTCCTTCTGTGTTTTTTTCTGCTTTTTAAAATCTTCATATCTTATATTTAATCTTAGGCATCATC | Assays designed by relax mode and have no off-target hits |
| BRCA2 | BRCA2_27 | 13 | 32910305 | 32910494 | ACACTGACGACATGGTTCTACAactgtgcccAAACACTACCTT | TACGGTAGCAGAGACTTGGTCTTCAGAATTGTCCCAAAAGAGCTA | 190 | 33 | actgtgcccAAACACTACCTTTTTAACTTAGTGAAAAATATTTAGTGAATGTGATTGATGGTACTTTAATTTTGTCACTTTGTGTTTTTATGTTTAGGTTTATTGCATTCTTCTGTGAAAAGAAGCTGTTCACAGAATGATTCTGAAGAACCAACTTTGTCCTTAACTAGCTCTTTTGGGACAATTCTGA | One primer sits in the repeat region |
| BRCA2 | BRCA2_28 | 13 | 32910395 | 32910590 | ACACTGACGACATGGTTCTACATGTTTAGGTTTATTGCATTCTTCTGTGA | TACGGTAGCAGAGACTTGGTCTACTGTAGTTTTTCCTTATTACATTTTGCT | 196 | 32 | TGTTTAGGTTTATTGCATTCTTCTGTGAAAAGAAGCTGTTCACAGAATGATTCTGAAGAACCAACTTTGTCCTTAACTAGCTCTTTTGGGACAATTCTGAGGAAATGTTCTAGAAATGAAACATGTTCTAATAATACAGTAATCTCTCAGGATCTTGATTATAAAGAAGCAAAATGTAATAAGGAAAAACTACAGT | Assays designed by relax mode and have no off-target hits |
| BRCA2 | BRCA2_29 | 13 | 32910464 | 32910654 | ACACTGACGACATGGTTCTACATCCTTAACTAGCTCTTTTGGGACA | TACGGTAGCAGAGACTTGGTCTGGATCATTTTCACACTGTCCTTCC | 191 | 35 | TCCTTAACTAGCTCTTTTGGGACAATTCTGAGGAAATGTTCTAGAAATGAAACATGTTCTAATAATACAGTAATCTCTCAGGATCTTGATTATAAAGAAGCAAAATGTAATAAGGAAAAACTACAGTTATTTATTACCCCAGAAGCTGATTCTCTGTCATGCCTGCAGGAAGGACAGTGTGAAAATGATCC |  |
| BRCA2 | BRCA2_30 | 13 | 32910603 | 32910766 | ACACTGACGACATGGTTCTACACAGAAGCTGATTCTCTGTCATGC | TACGGTAGCAGAGACTTGGTCTACTTTTCTGGGATTGAAAGTCAGTAT | 164 | 40 | CAGAAGCTGATTCTCTGTCATGCCTGCAGGAAGGACAGTGTGAAAATGATCCAAAAAGCAAAAAAGTTTCAGATATAAAAGAAGAGGTCTTGGCTGCAGCATGTCACCCAGTACAACATTCAAAAGTGGAATACAGTGATACTGACTTTCAATCCCAGAAAAGT | Assays designed by relax mode and have no off-target hits |
| BRCA2 | BRCA2_31 | 13 | 32910671 | 32910842 | ACACTGACGACATGGTTCTACATCAGATATAAAAGAAGAGGTCTTGGC | TACGGTAGCAGAGACTTGGTCTTGACTAGGTTTGACAGAACATCC | 172 | 38 | TCAGATATAAAAGAAGAGGTCTTGGCTGCAGCATGTCACCCAGTACAACATTCAAAAGTGGAATACAGTGATACTGACTTTCAATCCCAGAAAAGTCTTTTATATGATCATGAAAATGCCAGCACTCTTATTTTAACTCCTACTTCCAAGGATGTTCTGTCAAACCTAGTCA | Assays designed by relax mode and have no off-target hits |
| BRCA2 | BRCA2_32 | 13 | 32910787 | 32910961 | ACACTGACGACATGGTTCTACATGCCAGCACTCTTATTTTAACTCCT | TACGGTAGCAGAGACTTGGTCTAGCACATACATCTTGATTCTTTTCCA | 175 | 35 | TGCCAGCACTCTTATTTTAACTCCTACTTCCAAGGATGTTCTGTCAAACCTAGTCATGATTTCTAGAGGCAAAGAATCATACAAAATGTCAGACAAGCTCAAAGGTAACAATTATGAATCTGATGTTGAATTAACCAAAAATATTCCCATGGAAAAGAATCAAGATGTATGTGCT | Assays designed by relax mode and have no off-target hits |
| BRCA2 | BRCA2_33 | 13 | 32910870 | 32911041 | ACACTGACGACATGGTTCTACAAAATGTCAGACAAGCTCAAAGGT | TACGGTAGCAGAGACTTGGTCTTGTACCTTTCTTGAAGGTGATGCT | 172 | 33 | AAATGTCAGACAAGCTCAAAGGTAACAATTATGAATCTGATGTTGAATTAACCAAAAATATTCCCATGGAAAAGAATCAAGATGTATGTGCTTTAAATGAAAATTATAAAAACGTTGAGCTGTTGCCACCTGAAAAATACATGAGAGTAGCATCACCTTCAAGAAAGGTACA | Assays designed by relax mode and have no off-target hits |
| BRCA2 | BRCA2_34 | 13 | 32910936 | 32911134 | ACACTGACGACATGGTTCTACATGGAAAAGAATCAAGATGTATGTGCT | TACGGTAGCAGAGACTTGGTCTTCTTCAGAGTCTGGATTGACAGTTAT | 199 | 32 | TGGAAAAGAATCAAGATGTATGTGCTTTAAATGAAAATTATAAAAACGTTGAGCTGTTGCCACCTGAAAAATACATGAGAGTAGCATCACCTTCAAGAAAGGTACAATTCAACCAAAACACAAATCTAAGAGTAATCCAAAAAAATCAAGAAGAAACTACTTCAATTTCAAAAATAACTGTCAATCCAGACTCTGAAGA | Assays designed by relax mode and have no off-target hits |
| BRCA2 | BRCA2_35 | 13 | 32911048 | 32911246 | ACACTGACGACATGGTTCTACACCAAAACACAAATCTAAGAGTAATCCA | TACGGTAGCAGAGACTTGGTCTGTTTACACAAGTCAAGTCTGTTTCA | 199 | 31 | CCAAAACACAAATCTAAGAGTAATCCAAAAAAATCAAGAAGAAACTACTTCAATTTCAAAAATAACTGTCAATCCAGACTCTGAAGAACTTTTCTCAGACAATGAGAATAATTTTGTCTTCCAAGTAGCTAATGAAAGGAATAATCTTGCTTTAGGAAATACTAAGGAACTTCATGAAACAGACTTGACTTGTGTAAAC | Assays designed by relax mode and have no off-target hits |
| BRCA2 | BRCA2_36 | 13 | 32911114 | 32911305 | ACACTGACGACATGGTTCTACATGTCAATCCAGACTCTGAAGAACT | TACGGTAGCAGAGACTTGGTCTGCTTGTTTATCACCTGTGTCTCC | 192 | 35 | TGTCAATCCAGACTCTGAAGAACTTTTCTCAGACAATGAGAATAATTTTGTCTTCCAAGTAGCTAATGAAAGGAATAATCTTGCTTTAGGAAATACTAAGGAACTTCATGAAACAGACTTGACTTGTGTAAACGAACCCATTTTCAAGAACTCTACCATGGTTTTATATGGAGACACAGGTGATAAACAAGC | Assays designed by relax mode and have no off-target hits |
| BRCA2 | BRCA2_37 | 13 | 32911227 | 32911397 | ACACTGACGACATGGTTCTACACAGACTTGACTTGTGTAAACGAACC | TACGGTAGCAGAGACTTGGTCTGACCTAGAGTCATTTTTATATGCTGCTTT | 171 | 36 | CAGACTTGACTTGTGTAAACGAACCCATTTTCAAGAACTCTACCATGGTTTTATATGGAGACACAGGTGATAAACAAGCAACCCAAGTGTCAATTAAAAAAGATTTGGTTTATGTTCTTGCAGAGGAGAACAAAAATAGTGTAAAGCAGCATATAAAAATGACTCTAGGTC | Assays designed by relax mode and have no off-target hits |
| BRCA2 | BRCA2_38 | 13 | 32911338 | 32911529 | ACACTGACGACATGGTTCTACAATGTTCTTGCAGAGGAGAACAAA | TACGGTAGCAGAGACTTGGTCTAAGCTGTTCTGAAGCTACCTC | 192 | 34 | ATGTTCTTGCAGAGGAGAACAAAAATAGTGTAAAGCAGCATATAAAAATGACTCTAGGTCAAGATTTAAAATCGGACATCTCCTTGAATATAGATAAAATACCAGAAAAAAATAATGATTACATGAACAAATGGGCAGGACTCTTAGGTCCAATTTCAAATCACAGTTTTGGAGGTAGCTTCAGAACAGCTT | Assays designed by relax mode and have no off-target hits |
| BRCA2 | BRCA2_39 | 13 | 32911416 | 32911569 | ACACTGACGACATGGTTCTACATCTCCTTGAATATAGATAAAATACCAGAAAAAA | TACGGTAGCAGAGACTTGGTCTTTCTTAATGTTATGTTCAGAGAGCTTG | 154 | 33 | TCTCCTTGAATATAGATAAAATACCAGAAAAAAATAATGATTACATGAACAAATGGGCAGGACTCTTAGGTCCAATTTCAAATCACAGTTTTGGAGGTAGCTTCAGAACAGCTTCAAATAAGGAAATCAAGCTCTCTGAACATAACATTAAGAA | Assays designed by relax mode and have no off-target hits |
| BRCA2 | BRCA2_40 | 13 | 32911509 | 32911679 | ACACTGACGACATGGTTCTACAGAGGTAGCTTCAGAACAGCTTCA | TACGGTAGCAGAGACTTGGTCTGAGGCTTGCTCAGTTTCTTTTGATT | 171 | 34 | GAGGTAGCTTCAGAACAGCTTCAAATAAGGAAATCAAGCTCTCTGAACATAACATTAAGAAGAGCAAAATGTTCTTCAAAGATATTGAAGAACAATATCCTACTAGTTTAGCTTGTGTTGAAATTGTAAATACCTTGGCATTAGATAATCAAAAGAAACTGAGCAAGCCTC | Assays designed by relax mode and have no off-target hits |
| BRCA2 | BRCA2_41 | 13 | 32911535 | 32911731 | ACACTGACGACATGGTTCTACAAAGGAAATCAAGCTCTCTGAACA | TACGGTAGCAGAGACTTGGTCTTCAGAAACAACTACACTACTCTGTAAA | 197 | 32 | AAGGAAATCAAGCTCTCTGAACATAACATTAAGAAGAGCAAAATGTTCTTCAAAGATATTGAAGAACAATATCCTACTAGTTTAGCTTGTGTTGAAATTGTAAATACCTTGGCATTAGATAATCAAAAGAAACTGAGCAAGCCTCAGTCAATTAATACTGTATCTGCACATTTACAGAGTAGTGTAGTTGTTTCTGA | Assays designed by relax mode and have no off-target hits |
| BRCA2 | BRCA2_42 | 13 | 32911651 | 32911821 | ACACTGACGACATGGTTCTACAAGATAATCAAAAGAAACTGAGCAAGC | TACGGTAGCAGAGACTTGGTCTTCTGCCTTTTGGCTAGGTG | 171 | 33 | AGATAATCAAAAGAAACTGAGCAAGCCTCAGTCAATTAATACTGTATCTGCACATTTACAGAGTAGTGTAGTTGTTTCTGATTGTAAAAATAGTCATATAACCCCTCAGATGTTATTTTCCAAGCAGGATTTTAATTCAAACCATAATTTAACACCTAGCCAAAAGGCAGA | Assays designed by relax mode and have no off-target hits |
| BRCA2 | BRCA2_43 | 13 | 32911752 | 32911942 | ACACTGACGACATGGTTCTACACCCCTCAGATGTTATTTTCCAAGC | TACGGTAGCAGAGACTTGGTCTAGTCATCTGGTTTTCAGGCACTT | 191 | 35 | CCCCTCAGATGTTATTTTCCAAGCAGGATTTTAATTCAAACCATAATTTAACACCTAGCCAAAAGGCAGAAATTACAGAACTTTCTACTATATTAGAAGAATCAGGAAGTCAGTTTGAATTTACTCAGTTTAGAAAACCAAGCTACATATTGCAGAAGAGTACATTTGAAGTGCCTGAAAACCAGATGACT | Assays designed by relax mode and have no off-target hits |
| BRCA2 | BRCA2_44 | 13 | 32911796 | 32911973 | ACACTGACGACATGGTTCTACAAATTTAACACCTAGCCAAAAGGC | TACGGTAGCAGAGACTTGGTCTCTCTGCATTCCTCAGAAGTGGTC | 178 | 35 | AATTTAACACCTAGCCAAAAGGCAGAAATTACAGAACTTTCTACTATATTAGAAGAATCAGGAAGTCAGTTTGAATTTACTCAGTTTAGAAAACCAAGCTACATATTGCAGAAGAGTACATTTGAAGTGCCTGAAAACCAGATGACTATCTTAAAGACCACTTCTGAGGAATGCAGAG | Assays designed by relax mode and have no off-target hits |
| BRCA2 | BRCA2_45 | 13 | 32911913 | 32912084 | ACACTGACGACATGGTTCTACAACATTTGAAGTGCCTGAAAACCA | TACGGTAGCAGAGACTTGGTCTTTTTCAACAGGCCAGCAAACTTC | 172 | 41 | ACATTTGAAGTGCCTGAAAACCAGATGACTATCTTAAAGACCACTTCTGAGGAATGCAGAGATGCTGATCTTCATGTCATAATGAATGCCCCATCGATTGGTCAGGTAGACAGCAGCAAGCAATTTGAAGGTACAGTTGAAATTAAACGGAAGTTTGCTGGCCTGTTGAAAA | Assays designed by relax mode and have no off-target hits |
| BRCA2 | BRCA2_46 | 13 | 32912025 | 32912218 | ACACTGACGACATGGTTCTACAGCAGCAAGCAATTTGAAGGTACA | TACGGTAGCAGAGACTTGGTCTACTAAACAGTTTCACAGCTTTTTGC | 194 | 38 | GCAGCAAGCAATTTGAAGGTACAGTTGAAATTAAACGGAAGTTTGCTGGCCTGTTGAAAAATGACTGTAACAAAAGTGCTTCTGGTTATTTAACAGATGAAAATGAAGTGGGGTTTAGGGGCTTTTATTCTGCTCATGGCACAAAACTGAATGTTTCTACTGAAGCTCTGCAAAAAGCTGTGAAACTGTTTAGT | Assays designed by relax mode and have no off-target hits |
| BRCA2 | BRCA2_47 | 13 | 32912129 | 32912305 | ACACTGACGACATGGTTCTACAGAAGTGGGGTTTAGGGGCTTTTA | TACGGTAGCAGAGACTTGGTCTTGAAACAACAGAATCATGACATTTACTT | 177 | 37 | GAAGTGGGGTTTAGGGGCTTTTATTCTGCTCATGGCACAAAACTGAATGTTTCTACTGAAGCTCTGCAAAAAGCTGTGAAACTGTTTAGTGATATTGAGAATATTAGTGAGGAAACTTCTGCAGAGGTACATCCAATAAGTTTATCTTCAAGTAAATGTCATGATTCTGTTGTTTCA | Assays designed by relax mode and have no off-target hits |
| BRCA2 | BRCA2_48 | 13 | 32912216 | 32912409 | ACACTGACGACATGGTTCTACAAGTGATATTGAGAATATTAGTGAGGAAACT | TACGGTAGCAGAGACTTGGTCTACAAAAGTGCCAGTAGTCATTTCA | 194 | 27 | AGTGATATTGAGAATATTAGTGAGGAAACTTCTGCAGAGGTACATCCAATAAGTTTATCTTCAAGTAAATGTCATGATTCTGTTGTTTCAATGTTTAAGATAGAAAATCATAATGATAAAACTGTAAGTGAAAAAAATAATAAATGCCAACTGATATTACAAAATAATATTGAAATGACTACTGGCACTTTTGT | Assays designed by relax mode and have no off-target hits |
| BRCA2 | BRCA2_49 | 13 | 32912285 | 32912479 | ACACTGACGACATGGTTCTACATGTCATGATTCTGTTGTTTCAATGT | TACGGTAGCAGAGACTTGGTCTTCTACTGGCAGCAGTATATTTGTT | 195 | 26 | TGTCATGATTCTGTTGTTTCAATGTTTAAGATAGAAAATCATAATGATAAAACTGTAAGTGAAAAAAATAATAAATGCCAACTGATATTACAAAATAATATTGAAATGACTACTGGCACTTTTGTTGAAGAAATTACTGAAAATTACAAGAGAAATACTGAAAATGAAGATAACAAATATACTGCTGCCAGTAGA | Assays designed by relax mode and have no off-target hits |
| BRCA2 | BRCA2_50 | 13 | 32912391 | 32912574 | ACACTGACGACATGGTTCTACATGACTACTGGCACTTTTGTTGAAG | TACGGTAGCAGAGACTTGGTCTTGATCAGTAAATAGCAAGTCCGT | 184 | 30 | TGACTACTGGCACTTTTGTTGAAGAAATTACTGAAAATTACAAGAGAAATACTGAAAATGAAGATAACAAATATACTGCTGCCAGTAGAAATTCTCATAACTTAGAATTTGATGGCAGTGATTCAAGTAAAAATGATACTGTTTGTATTCATAAAGATGAAACGGACTTGCTATTTACTGATCA | Assays designed by relax mode and have no off-target hits |
| BRCA2 | BRCA2_51 | 13 | 32912431 | 32912629 | ACACTGACGACATGGTTCTACACAAGAGAAATACTGAAAATGAAGATAACAAAT | TACGGTAGCAGAGACTTGGTCTCTGAGTGTTTCCCTCCTTCATAA | 199 | 33 | CAAGAGAAATACTGAAAATGAAGATAACAAATATACTGCTGCCAGTAGAAATTCTCATAACTTAGAATTTGATGGCAGTGATTCAAGTAAAAATGATACTGTTTGTATTCATAAAGATGAAACGGACTTGCTATTTACTGATCAGCACAACATATGTCTTAAATTATCTGGCCAGTTTATGAAGGAGGGAAACACTCAG | Assays designed by relax mode and have no off-target hits |
| BRCA2 | BRCA2_52 | 13 | 32912534 | 32912717 | ACACTGACGACATGGTTCTACATGTATTCATAAAGATGAAACGGACTTG | TACGGTAGCAGAGACTTGGTCTACTGTTCTTTATTTGAAGTATTACCATGAC | 184 | 34 | TGTATTCATAAAGATGAAACGGACTTGCTATTTACTGATCAGCACAACATATGTCTTAAATTATCTGGCCAGTTTATGAAGGAGGGAAACACTCAGATTAAAGAAGATTTGTCAGATTTAACTTTTTTGGAAGTTGCGAAAGCTCAAGAAGCATGTCATGGTAATACTTCAAATAAAGAACAGT | Assays designed by relax mode and have no off-target hits |
| BRCA2 | BRCA2_53 | 13 | 32912600 | 32912792 | ACACTGACGACATGGTTCTACAGGCCAGTTTATGAAGGAGGGAAA | TACGGTAGCAGAGACTTGGTCTTCCCACTTGCAGTCTGAAAAAATG | 193 | 35 | GGCCAGTTTATGAAGGAGGGAAACACTCAGATTAAAGAAGATTTGTCAGATTTAACTTTTTTGGAAGTTGCGAAAGCTCAAGAAGCATGTCATGGTAATACTTCAAATAAAGAACAGTTAACTGCTACTAAAACGGAGCAAAATATAAAAGATTTTGAGACTTCTGATACATTTTTTCAGACTGCAAGTGGGA | Assays designed by relax mode and have no off-target hits |
| BRCA2 | BRCA2_54 | 13 | 32912680 | 32912872 | ACACTGACGACATGGTTCTACAAGAAGCATGTCATGGTAATACTTCAAA | TACGGTAGCAGAGACTTGGTCTAAAGTTATGCAATTCTTCTGGTTTCT | 193 | 30 | AGAAGCATGTCATGGTAATACTTCAAATAAAGAACAGTTAACTGCTACTAAAACGGAGCAAAATATAAAAGATTTTGAGACTTCTGATACATTTTTTCAGACTGCAAGTGGGAAAAATATTAGTGTCGCCAAAGAGTCATTTAATAAAATTGTAAATTTCTTTGATCAGAAACCAGAAGAATTGCATAACTTT | Assays designed by relax mode and have no off-target hits |
| BRCA2 | BRCA2_55 | 13 | 32912788 | 32912986 | ACACTGACGACATGGTTCTACATGGGAAAAATATTAGTGTCGCCAAAG | TACGGTAGCAGAGACTTGGTCTACCAACTGGGACACTTTCTTTCA | 199 | 30 | TGGGAAAAATATTAGTGTCGCCAAAGAGTCATTTAATAAAATTGTAAATTTCTTTGATCAGAAACCAGAAGAATTGCATAACTTTTCCTTAAATTCTGAATTACATTCTGACATAAGAAAGAACAAAATGGACATTCTAAGTTATGAGGAAACAGACATAGTTAAACACAAAATACTGAAAGAAAGTGTCCCAGTTGGT | Assays designed by relax mode and have no off-target hits |
| BRCA2 | BRCA2_56 | 13 | 32912851 | 32913037 | ACACTGACGACATGGTTCTACAACCAGAAGAATTGCATAACTTTTCCT | TACGGTAGCAGAGACTTGGTCTCTTTTCATCACGTTCGGGTTGTC | 187 | 36 | ACCAGAAGAATTGCATAACTTTTCCTTAAATTCTGAATTACATTCTGACATAAGAAAGAACAAAATGGACATTCTAAGTTATGAGGAAACAGACATAGTTAAACACAAAATACTGAAAGAAAGTGTCCCAGTTGGTACTGGAAATCAACTAGTGACCTTCCAGGGACAACCCGAACGTGATGAAAAG | Assays designed by relax mode and have no off-target hits |
| BRCA2 | BRCA2_57 | 13 | 32912966 | 32913164 | ACACTGACGACATGGTTCTACAAAAGAAAGTGTCCCAGTTGGT | TACGGTAGCAGAGACTTGGTCTTGGTGATTTCACTAGTACCTTGC | 199 | 39 | AAAGAAAGTGTCCCAGTTGGTACTGGAAATCAACTAGTGACCTTCCAGGGACAACCCGAACGTGATGAAAAGATCAAAGAACCTACTCTATTGGGTTTTCATACAGCTAGCGGGAAAAAAGTTAAAATTGCAAAGGAATCTTTGGACAAAGTGAAAAACCTTTTTGATGAAAAAGAGCAAGGTACTAGTGAAATCACCA | Assays designed by relax mode and have no off-target hits |
| BRCA2 | BRCA2_58 | 13 | 32913064 | 32913248 | ACACTGACGACATGGTTCTACATCATACAGCTAGCGGGAAAAAAGT | TACGGTAGCAGAGACTTGGTCTTGATCTCAATGGTCTCACATGCT | 185 | 38 | TCATACAGCTAGCGGGAAAAAAGTTAAAATTGCAAAGGAATCTTTGGACAAAGTGAAAAACCTTTTTGATGAAAAAGAGCAAGGTACTAGTGAAATCACCAGTTTTAGCCATCAATGGGCAAAGACCCTAAAGTACAGAGAGGCCTGTAAAGACCTTGAATTAGCATGTGAGACCATTGAGATCA | Assays designed by relax mode and have no off-target hits |
| BRCA2 | BRCA2_59 | 13 | 32913185 | 32913382 | ACACTGACGACATGGTTCTACAAAGACCCTAAAGTACAGAGAGGC | TACGGTAGCAGAGACTTGGTCTTGATGTTTTGAGATTTTCAGTTTGTCT | 198 | 37 | AAGACCCTAAAGTACAGAGAGGCCTGTAAAGACCTTGAATTAGCATGTGAGACCATTGAGATCACAGCTGCCCCAAAGTGTAAAGAAATGCAGAATTCTCTCAATAATGATAAAAACCTTGTTTCTATTGAGACTGTGGTGCCACCTAAGCTCTTAAGTGATAATTTATGTAGACAAACTGAAAATCTCAAAACATCA | Assays designed by relax mode and have no off-target hits |
| BRCA2 | BRCA2_60 | 13 | 32913301 | 32913486 | ACACTGACGACATGGTTCTACACCTTGTTTCTATTGAGACTGTGGTG | TACGGTAGCAGAGACTTGGTCTTCAATGACTGAATAAGGGGACTG | 186 | 33 | CCTTGTTTCTATTGAGACTGTGGTGCCACCTAAGCTCTTAAGTGATAATTTATGTAGACAAACTGAAAATCTCAAAACATCAAAAAGTATCTTTTTGAAAGTTAAAGTACATGAAAATGTAGAAAAAGAAACAGCAAAAAGTCCTGCAACTTGTTACACAAATCAGTCCCCTTATTCAGTCATTGA | Assays designed by relax mode and have no off-target hits |
| BRCA2 | BRCA2_61 | 13 | 32913356 | 32913547 | ACACTGACGACATGGTTCTACAAGACAAACTGAAAATCTCAAAACATCAAA | TACGGTAGCAGAGACTTGGTCTTGAAGTCTGACTCACAGAAGTTTTTC | 192 | 32 | AGACAAACTGAAAATCTCAAAACATCAAAAAGTATCTTTTTGAAAGTTAAAGTACATGAAAATGTAGAAAAAGAAACAGCAAAAAGTCCTGCAACTTGTTACACAAATCAGTCCCCTTATTCAGTCATTGAAAATTCAGCCTTAGCTTTTTACACAAGTTGTAGTAGAAAAACTTCTGTGAGTCAGACTTCA | Assays designed by relax mode and have no off-target hits |
| BRCA2 | BRCA2_62 | 13 | 32913476 | 32913669 | ACACTGACGACATGGTTCTACATCAGTCATTGAAAATTCAGCCTTAGC | TACGGTAGCAGAGACTTGGTCTTCAGCTATAGTACTGTTTGAATTATTTTCAT | 194 | 31 | TCAGTCATTGAAAATTCAGCCTTAGCTTTTTACACAAGTTGTAGTAGAAAAACTTCTGTGAGTCAGACTTCATTACTTGAAGCAAAAAAATGGCTTAGAGAAGGAATATTTGATGGTCAACCAGAAAGAATAAATACTGCAGATTATGTAGGAAATTATTTGTATGAAAATAATTCAAACAGTACTATAGCTGA | Assays designed by relax mode and have no off-target hits |
| BRCA2 | BRCA2_63 | 13 | 32913566 | 32913759 | ACACTGACGACATGGTTCTACATGGCTTAGAGAAGGAATATTTGATGGT | TACGGTAGCAGAGACTTGGTCTACCTCATCAGAATGGTAGGAATAGC | 194 | 31 | TGGCTTAGAGAAGGAATATTTGATGGTCAACCAGAAAGAATAAATACTGCAGATTATGTAGGAAATTATTTGTATGAAAATAATTCAAACAGTACTATAGCTGAAAATGACAAAAATCATCTCTCCGAAAAACAAGATACTTATTTAAGTAACAGTAGCATGTCTAACAGCTATTCCTACCATTCTGATGAGGT | Assays designed by relax mode and have no off-target hits |
| BRCA2 | BRCA2_64 | 13 | 32913649 | 32913831 | ACACTGACGACATGGTTCTACATTCAAACAGTACTATAGCTGAAAATGAC | TACGGTAGCAGAGACTTGGTCTTCAACATTCTTCAATACTGGCTCAA | 183 | 32 | TTCAAACAGTACTATAGCTGAAAATGACAAAAATCATCTCTCCGAAAAACAAGATACTTATTTAAGTAACAGTAGCATGTCTAACAGCTATTCCTACCATTCTGATGAGGTATATAATGATTCAGGATATCTCTCAAAAAATAAACTTGATTCTGGTATTGAGCCAGTATTGAAGAATGTTGA | Assays designed by relax mode and have no off-target hits |
| BRCA2 | BRCA2_65 | 13 | 32913735 | 32913933 | ACACTGACGACATGGTTCTACAGCTATTCCTACCATTCTGATGAGGT | TACGGTAGCAGAGACTTGGTCTACAAGTTCCTCAACGCAAATATC | 199 | 32 | GCTATTCCTACCATTCTGATGAGGTATATAATGATTCAGGATATCTCTCAAAAAATAAACTTGATTCTGGTATTGAGCCAGTATTGAAGAATGTTGAAGATCAAAAAAACACTAGTTTTTCCAAAGTAATATCCAATGTAAAAGATGCAAATGCATACCCACAAACTGTAAATGAAGATATTTGCGTTGAGGAACTTGT | Assays designed by relax mode and have no off-target hits |
| BRCA2 | BRCA2_66 | 13 | 32913854 | 32914036 | ACACTGACGACATGGTTCTACATCCAAAGTAATATCCAATGTAAAAGATGC | TACGGTAGCAGAGACTTGGTCTACTGGCTATCCTAAATGCAGGTG | 183 | 36 | TCCAAAGTAATATCCAATGTAAAAGATGCAAATGCATACCCACAAACTGTAAATGAAGATATTTGCGTTGAGGAACTTGTGACTAGCTCTTCACCCTGCAAAAATAAAAATGCAGCCATTAAATTGTCCATATCTAATAGTAATAATTTTGAGGTAGGGCCACCTGCATTTAGGATAGCCAGT | Assays designed by relax mode and have no off-target hits |
| BRCA2 | BRCA2_67 | 13 | 32913964 | 32914149 | ACACTGACGACATGGTTCTACATGCAGCCATTAAATTGTCCATATCT | TACGGTAGCAGAGACTTGGTCTTGGCAAATTTTTGATTTATTCTCGTTGTTT | 186 | 32 | TGCAGCCATTAAATTGTCCATATCTAATAGTAATAATTTTGAGGTAGGGCCACCTGCATTTAGGATAGCCAGTGGTAAAATCGTTTGTGTTTCACATGAAACAATTAAAAAAGTGAAAGACATATTTACAGACAGTTTCAGTAAAGTAATTAAGGAAAACAACGAGAATAAATCAAAAATTTGCCA | Assays designed by relax mode and have no off-target hits |
| BRCA2 | BRCA2_68 | 13 | 32914091 | 32914275 | ACACTGACGACATGGTTCTACAACAGACAGTTTCAGTAAAGTAATTAAGGA | TACGGTAGCAGAGACTTGGTCTTCTTCACTCTGAATGTCAGCAAA | 185 | 35 | ACAGACAGTTTCAGTAAAGTAATTAAGGAAAACAACGAGAATAAATCAAAAATTTGCCAAACGAAAATTATGGCAGGTTGTTACGAGGCATTGGATGATTCAGAGGATATTCTTCATAACTCTCTAGATAATGATGAATGTAGCACGCATTCACATAAGGTTTTTGCTGACATTCAGAGTGAAGA | Assays designed by relax mode and have no off-target hits |
| BRCA2 | BRCA2_69 | 13 | 32914156 | 32914353 | ACACTGACGACATGGTTCTACAAATTATGGCAGGTTGTTACGAGG | TACGGTAGCAGAGACTTGGTCTGTTTCCAAACTAACATCACAAGGT | 198 | 34 | AATTATGGCAGGTTGTTACGAGGCATTGGATGATTCAGAGGATATTCTTCATAACTCTCTAGATAATGATGAATGTAGCACGCATTCACATAAGGTTTTTGCTGACATTCAGAGTGAAGAAATTTTACAACATAACCAAAATATGTCTGGATTGGAGAAAGTTTCTAAAATATCACCTTGTGATGTTAGTTTGGAAAC | Assays designed by relax mode and have no off-target hits |
| BRCA2 | BRCA2_70 | 13 | 32914255 | 32914448 | ACACTGACGACATGGTTCTACATGCTGACATTCAGAGTGAAGAAA | TACGGTAGCAGAGACTTGGTCTCAGATTTTCCACTTGCTGTGCTAA | 194 | 34 | TGCTGACATTCAGAGTGAAGAAATTTTACAACATAACCAAAATATGTCTGGATTGGAGAAAGTTTCTAAAATATCACCTTGTGATGTTAGTTTGGAAACTTCAGATATATGTAAATGTAGTATAGGGAAGCTTCATAAGTCAGTCTCATCTGCAAATACTTGTGGGATTTTTAGCACAGCAAGTGGAAAATCTG | Assays designed by relax mode and have no off-target hits |
| BRCA2 | BRCA2_71 | 13 | 32914397 | 32914568 | ACACTGACGACATGGTTCTACAGTCTCATCTGCAAATACTTGTGGG | TACGGTAGCAGAGACTTGGTCTTGAGCTGGTCTGAATGTTCGTTA | 172 | 37 | GTCTCATCTGCAAATACTTGTGGGATTTTTAGCACAGCAAGTGGAAAATCTGTCCAGGTATCAGATGCTTCATTACAAAACGCAAGACAAGTGTTTTCTGAAATAGAAGATAGTACCAAGCAAGTCTTTTCCAAAGTATTGTTTAAAAGTAACGAACATTCAGACCAGCTCA | Assays designed by relax mode and have no off-target hits |
| BRCA2 | BRCA2_72 | 13 | 32914478 | 32914674 | ACACTGACGACATGGTTCTACAGCAAGACAAGTGTTTTCTGAAATAGAAG | TACGGTAGCAGAGACTTGGTCTGCTGTACTAAATCCAGAGAAAGCAGA | 197 | 35 | GCAAGACAAGTGTTTTCTGAAATAGAAGATAGTACCAAGCAAGTCTTTTCCAAAGTATTGTTTAAAAGTAACGAACATTCAGACCAGCTCACAAGAGAAGAAAATACTGCTATACGTACTCCAGAACATTTAATATCCCAAAAAGGCTTTTCATATAATGTGGTAAATTCATCTGCTTTCTCTGGATTTAGTACAGC | Assays designed by relax mode and have no off-target hits |
| BRCA2 | BRCA2_73 | 13 | 32914546 | 32914737 | ACACTGACGACATGGTTCTACATAACGAACATTCAGACCAGCTCA | TACGGTAGCAGAGACTTGGTCTTCCTCTAACACTCCCTTAACTTTGTG | 192 | 36 | TAACGAACATTCAGACCAGCTCACAAGAGAAGAAAATACTGCTATACGTACTCCAGAACATTTAATATCCCAAAAAGGCTTTTCATATAATGTGGTAAATTCATCTGCTTTCTCTGGATTTAGTACAGCAAGTGGAAAGCAAGTTTCCATTTTAGAAAGTTCCTTACACAAAGTTAAGGGAGTGTTAGAGGA | Assays designed by relax mode and have no off-target hits |
| BRCA2 | BRCA2_74 | 13 | 32914658 | 32914834 | ACACTGACGACATGGTTCTACATCTGGATTTAGTACAGCAAGTGGA | TACGGTAGCAGAGACTTGGTCTTGGGTTTCTCTTATCAACACGAGG | 177 | 36 | TCTGGATTTAGTACAGCAAGTGGAAAGCAAGTTTCCATTTTAGAAAGTTCCTTACACAAAGTTAAGGGAGTGTTAGAGGAATTTGATTTAATCAGAACTGAGCATAGTCTTCACTATTCACCTACGTCTAGACAAAATGTATCAAAAATACTTCCTCGTGTTGATAAGAGAAACCCA | Assays designed by relax mode and have no off-target hits |
| BRCA2 | BRCA2_75 | 13 | 32914749 | 32914934 | ACACTGACGACATGGTTCTACATCAGAACTGAGCATAGTCTTCACT | TACGGTAGCAGAGACTTGGTCTAGTGATTATTTTCTGAAGAACCACCTT | 186 | 33 | TCAGAACTGAGCATAGTCTTCACTATTCACCTACGTCTAGACAAAATGTATCAAAAATACTTCCTCGTGTTGATAAGAGAAACCCAGAGCACTGTGTAAACTCAGAAATGGAAAAAACCTGCAGTAAAGAATTTAAATTATCAAATAACTTAAATGTTGAAGGTGGTTCTTCAGAAAATAATCACT | Assays designed by relax mode and have no off-target hits |
| BRCA2 | BRCA2_76 | 13 | 32914851 | 32915046 | ACACTGACGACATGGTTCTACACAGAAATGGAAAAAACCTGCAGTA | TACGGTAGCAGAGACTTGGTCTGCCTGTTCTTTTCCCAAAACATGAA | 196 | 32 | CAGAAATGGAAAAAACCTGCAGTAAAGAATTTAAATTATCAAATAACTTAAATGTTGAAGGTGGTTCTTCAGAAAATAATCACTCTATTAAAGTTTCTCCATATCTCTCTCAATTTCAACAAGACAAACAACAGTTGGTATTAGGAACCAAAGTGTCACTTGTTGAGAACATTCATGTTTTGGGAAAAGAACAGGC | Assays designed by relax mode and have no off-target hits |
| BRCA2 | BRCA2_77 | 13 | 32914958 | 32915151 | ACACTGACGACATGGTTCTACATCTCAATTTCAACAAGACAAACAACA | TACGGTAGCAGAGACTTGGTCTTCTGAATCTTTGGAGTAAGTAGAACA | 194 | 33 | TCTCAATTTCAACAAGACAAACAACAGTTGGTATTAGGAACCAAAGTGTCACTTGTTGAGAACATTCATGTTTTGGGAAAAGAACAGGCTTCACCTAAAAACGTAAAAATGGAAATTGGTAAAACTGAAACTTTTTCTGATGTTCCTGTGAAAACAAATATAGAAGTTTGTTCTACTTACTCCAAAGATTCAGA | Assays designed by relax mode and have no off-target hits |
| BRCA2 | BRCA2_78 | 13 | 32915045 | 32915243 | ACACTGACGACATGGTTCTACAGCTTCACCTAAAAACGTAAAAATGGAA | TACGGTAGCAGAGACTTGGTCTGTGTGGCATGACTTGGCAGTTTA | 199 | 34 | GCTTCACCTAAAAACGTAAAAATGGAAATTGGTAAAACTGAAACTTTTTCTGATGTTCCTGTGAAAACAAATATAGAAGTTTGTTCTACTTACTCCAAAGATTCAGAAAACTACTTTGAAACAGAAGCAGTAGAAATTGCTAAAGCTTTTATGGAAGATGATGAACTGACAGATTCTAAACTGCCAAGTCATGCCACAC | Assays designed by relax mode and have no off-target hits |
| BRCA2 | BRCA2_79 | 13 | 32915188 | 32915372 | ACACTGACGACATGGTTCTACAAGCTTTTATGGAAGATGATGAACTGA | TACGGTAGCAGAGACTTGGTCTTAGTGATTGGCAACACGAAAGGT | 185 | 38 | AGCTTTTATGGAAGATGATGAACTGACAGATTCTAAACTGCCAAGTCATGCCACACATTCTCTTTTTACATGTCCCGAAAATGAGGAAATGGTTTTGTCAAATTCAAGAATTGGAAAAAGAAGAGGAGAGCCCCTTATCTTAGTGGGTAAGTGTTCATTTTTACCTTTCGTGTTGCCAATCACTA | Assays designed by relax mode and have no off-target hits |
| BRCA2 | BRCA2_80 | 13 | 32918559 | 32918755 | ACACTGACGACATGGTTCTACACACTATTTGTTGTAAGTATTTTTGTTTAACATTT | TACGGTAGCAGAGACTTGGTCTTCTTGATTTTCTATTATCCTGTCAAATTCAT | 197 | 23 | CACTATTTGTTGTAAGTATTTTTGTTTAACATTTAAAGAGTCAATACTTTAGCTTTAAAAAAATGGTCTATAGACTTTTGAGAAATAAAACTGATATTATTTGCCTTAAAAACATATATGAAATATTTCTTTTTAGGAGAACCCTCAATCAAAAGAAACTTATTAAATGAATTTGACAGGATAATAGAAAATCAAGA | Assays designed by relax mode and have no off-target hits |
| BRCA2 | BRCA2_81 | 13 | 32918683 | 32918853 | ACACTGACGACATGGTTCTACAATTTCTTTTTAGGAGAACCCTCAATCAA | TACGGTAGCAGAGACTTGGTCTGTCAGAATATTATATACCATACCTATAGAGGGAGA | 171 | 29 | ATTTCTTTTTAGGAGAACCCTCAATCAAAAGAAACTTATTAAATGAATTTGACAGGATAATAGAAAATCAAGAAAAATCCTTAAAGGCTTCAAAAAGCACTCCAGATGGTAAAATTAGCTTTTTATTTATATCTGTTCTCCCTCTATAGGTATGGTATATAATAttctgac | Assays designed by relax mode and have no off-target hits |
| BRCA2 | BRCA2_83 | 13 | 32920895 | 32921044 | ACACTGACGACATGGTTCTACAGTATTTACAGTAACATGGATATTCTCTTAGATTT | TACGGTAGCAGAGACTTGGTCTACATGTCTTACCGAAAGGGTACA | 150 | 31 | GTATTTACAGTAACATGGATATTCTCTTAGATTTTAACTAATATGTAATATAAAATAATTGTTTCCTAGGCACAATAAAAGATCGAAGATTGTTTATGCATCATGTTTCTTTAGAGCCGATTACCTGTGTACCCTTTCGGTAAGACATGT | Assays designed by relax mode and have no off-target hits |
| BRCA2 | BRCA2_127 | 13 | 32920953 | 32921102 | ACACTGACGACATGGTTCTACATTGTTTCCTAGGCACAATAAAAGA | TACGGTAGCAGAGACTTGGTCTTGTTCATTTATAAAAACGAGACTTTTC | 150 | 31 | TTGTTTCCTAGGCACAATAAAAGATCGAAGATTGTTTATGCATCATGTTTCTTTAGAGCCGATTACCTGTGTACCCTTTCGGTAAGACATGTTTAAATTTTTCTAAATTCTAATACAGTATGAGAAAAGTCTCGTTTTTATAAATGAACA |  |
| BRCA2 | BRCA2_85 | 13 | 32928912 | 32929110 | ACACTGACGACATGGTTCTACACAAATGAGGGTCTGCAACAAAGG | TACGGTAGCAGAGACTTGGTCTTGCTTGAAGATTTTTCCAAAGTCAG | 199 | 35 | CAAATGAGGGTCTGCAACAAAGGCATATTCCTAAATATTTATATGTGTACTAGTCAATAAACTTATATATTTTCTCCCCATTGCAGCACAACTAAGGAACGTCAAGAGATACAGAATCCAAATTTTACCGCACCTGGTCAAGAATTTCTGTCTAAATCTCATTTGTATGAACATCTGACTTTGGAAAAATCTTCAAGCA | Assays designed by relax mode and have no off-target hits |
| BRCA2 | BRCA2_86 | 13 | 32928997 | 32929195 | ACACTGACGACATGGTTCTACAGCACAACTAAGGAACGTCAAGAG | TACGGTAGCAGAGACTTGGTCTGGTCTGCCTGTAGTAATCAAGTG | 199 | 36 | GCACAACTAAGGAACGTCAAGAGATACAGAATCCAAATTTTACCGCACCTGGTCAAGAATTTCTGTCTAAATCTCATTTGTATGAACATCTGACTTTGGAAAAATCTTCAAGCAATTTAGCAGTTTCAGGACATCCATTTTATCAAGTTTCTGCTACAAGAAATGAAAAAATGAGACACTTGATTACTACAGGCAGACC | Assays designed by relax mode and have no off-target hits |
| BRCA2 | BRCA2_87 | 13 | 32929113 | 32929288 | ACACTGACGACATGGTTCTACATTAGCAGTTTCAGGACATCCATT | TACGGTAGCAGAGACTTGGTCTTGTCTGTTTTCCTCCAAGTTAATATTCC | 176 | 35 | TTAGCAGTTTCAGGACATCCATTTTATCAAGTTTCTGCTACAAGAAATGAAAAAATGAGACACTTGATTACTACAGGCAGACCAACCAAAGTCTTTGTTCCACCTTTTAAAACTAAATCACATTTTCACAGAGTTGAACAGTGTGTTAGGAATATTAACTTGGAGGAAAACAGACA |  |
| BRCA2 | BRCA2_88 | 13 | 32929181 | 32929377 | ACACTGACGACATGGTTCTACATACTACAGGCAGACCAACCAAAG | TACGGTAGCAGAGACTTGGTCTTGGAGTTGTTTTTGTTAAACTGATGA | 197 | 34 | TACTACAGGCAGACCAACCAAAGTCTTTGTTCCACCTTTTAAAACTAAATCACATTTTCACAGAGTTGAACAGTGTGTTAGGAATATTAACTTGGAGGAAAACAGACAAAAGCAAAACATTGATGGACATGGCTCTGATGATAGTAAAAATAAGATTAATGACAATGAGATTCATCAGTTTAACAAAAACAACTCCA | Assays designed by relax mode and have no off-target hits |
| BRCA2 | BRCA2_89 | 13 | 32929315 | 32929467 | ACACTGACGACATGGTTCTACACTGATGATAGTAAAAATAAGATTAATGACAATGAG | TACGGTAGCAGAGACTTGGTCTACTGAAAGGCAAAAATTCATCACACA | 153 | 31 | CTGATGATAGTAAAAATAAGATTAATGACAATGAGATTCATCAGTTTAACAAAAACAACTCCAATCAAGCAGTAGCTGTAACTTTCACAAAGTGTGAAGAAGAACCTTTAGGTATTGTATGACAATTTGTGTGATGAATTTTTGCCTTTCAGT |  |
| BRCA2 | BRCA2_90 | 13 | 32930503 | 32930690 | ACACTGACGACATGGTTCTACAGGCCAGGGGTTGTGCTTTTTA | TACGGTAGCAGAGACTTGGTCTTTCGAGGCAGAGTGGATGTTTTT | 188 | 37 | ggccAGGGGTTGTGCTTTTTAAATTTCAATTTTATTTTTGCTAAGTATTTATTCTTTGATAGATTTAATTACAAGTCTTCAGAATGCCAGAGATATACAGGATATGCGAATTAAGAAGAAACAAAGGCAACGCGTCTTTCCACAGCCAGGCAGTCTGTATCTTGCAAAAACATCCACTCTGCCTCGAA | Assays designed by relax mode and have no off-target hits |
| BRCA2 | BRCA2_91 | 13 | 32930607 | 32930790 | ACACTGACGACATGGTTCTACATGCGAATTAAGAAGAAACAAAGGC | TACGGTAGCAGAGACTTGGTCTACACTCTGTCATAAAAGCCATCAGT | 184 | 45 | TGCGAATTAAGAAGAAACAAAGGCAACGCGTCTTTCCACAGCCAGGCAGTCTGTATCTTGCAAAAACATCCACTCTGCCTCGAATCTCTCTGAAAGCAGCAGTAGGAGGCCAAGTTCCCTCTGCGTGTTCTCATAAACAGGTATGTGTTTGTCTACAATACTGATGGCTTTTATGACAGAGTGT | Assays designed by relax mode and have no off-target hits |
| BRCA2 | BRCA2_92 | 13 | 32931809 | 32932007 | ACACTGACGACATGGTTCTACATTGTTTTTATTGTGTGATACATGTTTACTTT | TACGGTAGCAGAGACTTGGTCTAGCCAACTGTATTCCTTTTCCAGT | 199 | 30 | TTGTTTTTATTGTGTGATACATGTTTACTTTAAATTGTTTTTCTTTTTTGTGTGTGTTTATTTTGTGTAGCTGTATACGTATGGCGTTTCTAAACATTGCATAAAAATTAACAGCAAAAATGCAGAGTCTTTTCAGTTTCACACTGAAGATTATTTTGGTAAGGAAAGTTTATGGACTGGAAAAGGAATACAGTTGGCT | Assays designed by relax mode and have no off-target hits |
| BRCA2 | BRCA2_93 | 13 | 32931907 | 32932099 | ACACTGACGACATGGTTCTACAGCATAAAAATTAACAGCAAAAATGCAG | TACGGTAGCAGAGACTTGGTCTAAAGTTAACACACAATCTTTTTGCATAG | 193 | 36 | GCATAAAAATTAACAGCAAAAATGCAGAGTCTTTTCAGTTTCACACTGAAGATTATTTTGGTAAGGAAAGTTTATGGACTGGAAAAGGAATACAGTTGGCTGATGGTGGATGGCTCATACCCTCCAATGATGGAAAGGCTGGAAAAGAAGAATTTTATAGGTACTCTATGCAAAAAGATTGTGTGTTAACTTT | Assays designed by relax mode and have no off-target hits |
| BRCA2 | BRCA2_94 | 13 | 32936578 | 32936771 | ACACTGACGACATGGTTCTACATGTACAGAGAATAGTTGTAGTTGTTGA | TACGGTAGCAGAGACTTGGTCTAGGAAAGGCACATTCCATAGCTG | 194 | 36 | TGTACAGAGAATAGTTGTAGTTGTTGAATTCAGTATCATCCTATGTGGTTTTTATGATAATATTCTACTTTTATTTGTTCAGGGCTCTGTGTGACACTCCAGGTGTGGATCCAAAGCTTATTTCTAGAATTTGGGTTTATAATCACTATAGATGGATCATATGGAAACTGGCAGCTATGGAATGTGCCTTTCCT | Assays designed by relax mode and have no off-target hits |
| BRCA2 | BRCA2_95 | 13 | 32936661 | 32936831 | ACACTGACGACATGGTTCTACAGCTCTGTGTGACACTCCAGGT | TACGGTAGCAGAGACTTGGTCTCCTGTATTTTAGTTGAAGAAGCACCC | 171 | 41 | GCTCTGTGTGACACTCCAGGTGTGGATCCAAAGCTTATTTCTAGAATTTGGGTTTATAATCACTATAGATGGATCATATGGAAACTGGCAGCTATGGAATGTGCCTTTCCTAAGGAATTTGCTAATAGATGCCTAAGCCCAGAAAGGGTGCTTCTTCAACTAAAATACAGG | Assays designed by relax mode and have no off-target hits |
| BRCA2 | BRCA2_96 | 13 | 32936738 | 32936933 | ACACTGACGACATGGTTCTACAATGGAAACTGGCAGCTATGGAAT | TACGGTAGCAGAGACTTGGTCTGACAACTGGCTTGTGCAACATTT | 196 | 40 | ATGGAAACTGGCAGCTATGGAATGTGCCTTTCCTAAGGAATTTGCTAATAGATGCCTAAGCCCAGAAAGGGTGCTTCTTCAACTAAAATACAGGCAAGTTTAAAGCATTACATTACGTAATCATATACGGCAGTATGGTTAAGGTTTCTGTGTAGTCTGTGACTTCCATGTCAAAATGTTGCACAAGCCAGTTGTC | Assays designed by relax mode and have no off-target hits |
| BRCA2 | BRCA2_97 | 13 | 32937263 | 32937433 | ACACTGACGACATGGTTCTACATGGAATTCTAGAGTCACACTTCCTAA | TACGGTAGCAGAGACTTGGTCTTGCGCTCAATGAAATTATGTCAGAAAC | 171 | 35 | TGGAATTCTAGAGTCACACTTCCTAAAATATGCATTTTTGTTTTCACTTTTAGATATGATACGGAAATTGATAGAAGCAGAAGATCGGCTATAAAAAAGATAATGGAAAGGGATGACACAGCTGCAAAAACACTTGTTCTCTGTGTTTCTGACATAATTTCATTGAGCGCA | Assays designed by relax mode and have no off-target hits |
| BRCA2 | BRCA2_98 | 13 | 32937366 | 32937563 | ACACTGACGACATGGTTCTACATGGAAAGGGATGACACAGC | TACGGTAGCAGAGACTTGGTCTTCTTTAAGACAGCTAAGAGGGGAG | 198 | 40 | TGGAAAGGGATGACACAGCTGCAAAAACACTTGTTCTCTGTGTTTCTGACATAATTTCATTGAGCGCAAATATATCTGAAACTTCTAGCAATAAAACTAGTAGTGCAGATACCCAAAAAGTGGCCATTATTGAACTTACAGATGGGTGGTATGCTGTTAAGGCCCAGTTAGATCCTCCCCTCTTAGCTGTCTTAAAGA | Assays designed by relax mode and have no off-target hits |
| BRCA2 | BRCA2_99 | 13 | 32937465 | 32937648 | ACACTGACGACATGGTTCTACAGTAGTGCAGATACCCAAAAAGTGG | TACGGTAGCAGAGACTTGGTCTGCTTCAAGAGGTGTACAGGCATC | 184 | 47 | GTAGTGCAGATACCCAAAAAGTGGCCATTATTGAACTTACAGATGGGTGGTATGCTGTTAAGGCCCAGTTAGATCCTCCCCTCTTAGCTGTCTTAAAGAATGGCAGACTGACAGTTGGTCAGAAGATTATTCTTCATGGAGCAGAACTGGTGGGCTCTCCTGATGCCTGTACACCTCTTGAAGC | Assays designed by relax mode and have no off-target hits |
| BRCA2 | BRCA2_100 | 13 | 32937594 | 32937785 | ACACTGACGACATGGTTCTACATTCTTCATGGAGCAGAACTGGTG | TACGGTAGCAGAGACTTGGTCTTCAGTACATCTAAGAAATTGAGCATCC | 192 | 35 | TTCTTCATGGAGCAGAACTGGTGGGCTCTCCTGATGCCTGTACACCTCTTGAAGCCCCAGAATCTCTTATGTTAAAGGTAAATTAATTTGCACTCTTGGTAAAAATCAGTCATTGATTCAGTTAAATTCTAGAAGTTTTACATTTAAATTTTAAATGCTTACTAAGGATGCTCAATTTCTTAGATGTACTGA |  |
| BRCA2 | BRCA2_101 | 13 | 32944423 | 32944616 | ACACTGACGACATGGTTCTACATTTTTAAAGTGAATATTTTTAAGGCAGTTCT | TACGGTAGCAGAGACTTGGTCTCAGAGGAAAAGGTCTAGGGTCAG | 194 | 32 | TTTTTAAAGTGAATATTTTTAAGGCAGTTCTAGAAGAATGAAAACTCTTATGATATCTGTAATAGAATTGAATACATATTTAACTACTAAATCAATATATTTATTAATTTGTCCAGATTTCTGCTAACAGTACTCGGCCTGCTCGCTGGTATACCAAACTTGGATTCTTTCCTGACCCTAGACCTTTTCCTCTG | Assays designed by relax mode and have no off-target hits |
| BRCA2 | BRCA2_102 | 13 | 32944558 | 32944739 | ACACTGACGACATGGTTCTACAGGCCTGCTCGCTGGTAT | TACGGTAGCAGAGACTTGGTCTATCAAAAGAAAGAAATATATGGTAAGTTTCAAG | 182 | 39 | GGCCTGCTCGCTGGTATACCAAACTTGGATTCTTTCCTGACCCTAGACCTTTTCCTCTGCCCTTATCATCGCTTTTCAGTGATGGAGGAAATGTTGGTTGTGTTGATGTAATTATTCAAAGAGCATACCCTATACAGGTATGATGTATTCTTGAAACTTACCATATATTTCTTTCTTTTGAT | Assays designed by relax mode and have no off-target hits |
| BRCA2 | BRCA2_103 | 13 | 32945018 | 32945192 | ACACTGACGACATGGTTCTACACTGTGCCTGGCCTGATACAATTA | TACGGTAGCAGAGACTTGGTCTCTAGTCTCTTTTGTTGGGCCTCC | 175 | 38 | ctgtgcctggccTGATACAATTAACTTGAATGTTATATATGTGACTTTTTTGGTGTGTGTAACACATTATTACAGTGGATGGAGAAGACATCATCTGGATTATACATATTTCGCAATGAAAGAGAGGAAGAAAAGGAAGCAGCAAAATATGTGGAGGCCCAACAAAAGAGACTAG | Assays designed by relax mode and have no off-target hits |
| BRCA2 | BRCA2_104 | 13 | 32945088 | 32945279 | ACACTGACGACATGGTTCTACATACAGTGGATGGAGAAGACATCA | TACGGTAGCAGAGACTTGGTCTTCATATTAGAAATAACAATGTGTACCATATAACT | 192 | 34 | TACAGTGGATGGAGAAGACATCATCTGGATTATACATATTTCGCAATGAAAGAGAGGAAGAAAAGGAAGCAGCAAAATATGTGGAGGCCCAACAAAAGAGACTAGAAGCCTTATTCACTAAAATTCAGGAGGAATTTGAAGAACATGAAGGTAAAATTAGTTATATGGTACACATTGTTATTTCTAATATGA | Assays designed by relax mode and have no off-target hits |
| BRCA2 | BRCA2_105 | 13 | 32950735 | 32950927 | ACACTGACGACATGGTTCTACATGCTTGGTTCTTTAGTTTTAGTTGCT | TACGGTAGCAGAGACTTGGTCTTCAAGGTAAGCTGGGTCTGC | 193 | 37 | TGCTTGGTTCTTTAGTTTTAGTTGCTTTTGAATTTACAGTTTAGTGAATTAATAATCCTTTTGTTTTCTTAGAAAACACAACAAAACCATATTTACCATCACGTGCACTAACAAGACAGCAAGTTCGTGCTTTGCAAGATGGTGCAGAGCTTTATGAAGCAGTGAAGAATGCAGCAGACCCAGCTTACCTTGA |  |
| BRCA2 | BRCA2_106 | 13 | 32950838 | 32951010 | ACACTGACGACATGGTTCTACATGCACTAACAAGACAGCAAGTTC | TACGGTAGCAGAGACTTGGTCTCCTGTGATGGCCAGAGAGTCTAA | 173 | 45 | TGCACTAACAAGACAGCAAGTTCGTGCTTTGCAAGATGGTGCAGAGCTTTATGAAGCAGTGAAGAATGCAGCAGACCCAGCTTACCTTGAGGTGAGAGAGTAAGAGGACATATAATGAGGCTTGATGATTATTCAAGGTGAGAAGCTGTTTTAGACTCTCTGGCCATCACAGG | Assays designed by relax mode and have no off-target hits |
| BRCA2 | BRCA2_107 | 13 | 32953398 | 32953576 | ACACTGACGACATGGTTCTACAAGATGGAACTTTTTTGTTCTGATTGC | TACGGTAGCAGAGACTTGGTCTTTCCTTTTGTTCAGCAGATTCCA | 179 | 36 | AGATGGAACTTTTTTGTTCTGATTGCTTTTTATTCCAATATCTTAAATGGTCACAGGGTTATTTCAGTGAAGAGCAGTTAAGAGCCTTGAATAATCACAGGCAAATGTTGAATGATAAGAAACAAGCTCAGATCCAGTTGGAAATTAGGAAGGCCATGGAATCTGCTGAACAAAAGGAA |  |
| BRCA2 | BRCA2_108 | 13 | 32953496 | 32953694 | ACACTGACGACATGGTTCTACAAGGCAAATGTTGAATGATAAGAAACA | TACGGTAGCAGAGACTTGGTCTAAGTTAATAAAACTGATAAAAACAAAGCATTTAC | 199 | 35 | AGGCAAATGTTGAATGATAAGAAACAAGCTCAGATCCAGTTGGAAATTAGGAAGGCCATGGAATCTGCTGAACAAAAGGAACAAGGTTTATCAAGGGATGTCACAACCGTGTGGAAGTTGCGTATTGTAAGCTATTCAAAAAAAGAAAAAGATTCAGGTAAGTATGTAAATGCTTTGTTTTTATCAGTTTTATTAACTT | Assays designed by relax mode and have no off-target hits |
| BRCA2 | BRCA2_109 | 13 | 32953847 | 32954025 | ACACTGACGACATGGTTCTACACACTTCTTCCATTGCATCTTTCTCA | TACGGTAGCAGAGACTTGGTCTTTTGTCGCTGCTAACTGTATGTT | 179 | 34 | CACTTCTTCCATTGCATCTTTCTCATCTTTCTCCAAACAGTTATACTGAGTATTTGGCGTCCATCATCAGATTTATATTCTCTGTTAACAGAAGGAAAGAGATACAGAATTTATCATCTTGCAACTTCAAAATCTAAAAGTAAATCTGAAAGAGCTAACATACAGTTAGCAGCGACAAA | Assays designed by relax mode and have no off-target hits |
| BRCA2 | BRCA2_110 | 13 | 32953901 | 32954099 | ACACTGACGACATGGTTCTACATGGCGTCCATCATCAGATTTATATTC | TACGGTAGCAGAGACTTGGTCTACTAACAAGCACTTATCAAAACTGAAA | 199 | 33 | TGGCGTCCATCATCAGATTTATATTCTCTGTTAACAGAAGGAAAGAGATACAGAATTTATCATCTTGCAACTTCAAAATCTAAAAGTAAATCTGAAAGAGCTAACATACAGTTAGCAGCGACAAAAAAAACTCAGTATCAACAACTACCGGTACAAACCTTTCATTGTAATTTTTCAGTTTTGATAAGTGCTTGTTAGT | Assays designed by relax mode and have no off-target hits |
| BRCA2 | BRCA2_111 | 13 | 32954047 | 32954235 | ACACTGACGACATGGTTCTACAACCGGTACAAACCTTTCATTGT | TACGGTAGCAGAGACTTGGTCTGAACAAGATGGCTGAAAGTCTGG | 189 | 35 | ACCGGTACAAACCTTTCATTGTAATTTTTCAGTTTTGATAAGTGCTTGTTAGTTTATGGAATCTCCATATGTTGAATTTTTGTTTTGTTTTCTGTAGGTTTCAGATGAAATTTTATTTCAGATTTACCAGCCACGGGAGCCCCTTCACTTCAGCAAATTTTTAGATCCAGACTTTCAGCCATCTTGTTC | Assays designed by relax mode and have no off-target hits |
| BRCA2 | BRCA2_112 | 13 | 32954171 | 32954369 | ACACTGACGACATGGTTCTACATACCAGCCACGGGAGCC | TACGGTAGCAGAGACTTGGTCTTGGTAGCTCCAACTAATCATAAGAGA | 199 | 35 | TACCAGCCACGGGAGCCCCTTCACTTCAGCAAATTTTTAGATCCAGACTTTCAGCCATCTTGTTCTGAGGTGGACCTAATAGGATTTGTCGTTTCTGTTGTGAAAAAAACAGGTAATGCACAATATAGTTAATTTTTTTTATTGATTCTTTTAAAAAACATTGTCTTTTAAAATCTCTTATGATTAGTTGGAGCTACCA | Assays designed by relax mode and have no off-target hits |
| BRCA2 | BRCA2_Intron_24__region_1__1 | 13 | 32955429 | 32955627 | ACACTGACGACATGGTTCTACAGGGGAGGGAGATTACAAACAAAG | TACGGTAGCAGAGACTTGGTCTACCTCACTCCCTCAATTCCTTCA | 199 | 40 | ggggagggagattacaaacaaagaaataagtaatatacatgtgtatagtttttttagtgctcagaaaaaaaattaagtgggtaagggggtaatgtcagagaagagagagggatgtaattttagattgagaggtgaggagagagacctccctggaaagctgacatttgagtgaagcttgaaggaattgagggagtgaggt | Two primers sits in the repeat region |
| BRCA2 | BRCA2_Intron_24__region_1__2 | 13 | 32955521 | 32955716 | ACACTGACGACATGGTTCTACATGTCAGAGAAGAGAGAGGGATGT | TACGGTAGCAGAGACTTGGTCTCAATGCTTCACGGCACACTAGG | 196 | 49 | tgtcagagaagagagagggatgtaattttagattgagaggtgaggagagagacctccctggaaagctgacatttgagtgaagcttgaaggaattgagggagtgaggtgaggcatgtggccatctggggaaagctttccaggcaattacaaaggccgcagtacagcaggatcatgcctagtgtgccgtgaagcattg | Two primers sits in the repeat region |
| BRCA2 | BRCA2_Intron_24__region_1__3 | 13 | 32955600 | 32955779 | ACACTGACGACATGGTTCTACAAAGCTTGAAGGAATTGAGGGAGT | TACGGTAGCAGAGACTTGGTCTCACACGACCTGGCTCTTCAC | 180 | 53 | aagcttgaaggaattgagggagtgaggtgaggcatgtggccatctggggaaagctttccaggcaattacaaaggccgcagtacagcaggatcatgcctagtgtgccgtgaagcattggcagagaccagagagtgagaagtaacatccagggacagaggcagtgaagagccaggtcgtgtg | Two primers sits in the repeat region |
| BRCA2 | BRCA2_Intron_24__region_1__4 | 13 | 32955731 | 32955909 | ACACTGACGACATGGTTCTACAGTGAGAAGTAACATCCAGGGACA | TACGGTAGCAGAGACTTGGTCTCCCCTACAGCCTATTCTCAACAC | 179 | 51 | gtgagaagtaacatccagggacagaggcagtgaagagccaggtcgtgtgggggtccttgtgtggactgtaacttcctgtgatgacaggaagtcacaggaaaattccaggtagagggacactgtctgacaggttttcacagaatcattcaggccactgtgttgagaataggctgtagggg | Two primers sits in the repeat region |
| BRCA2 | BRCA2_Intron_24__region_1__5 | 13 | 32955824 | 32956006 | ACACTGACGACATGGTTCTACAACAGGAAAATTCCAGGTAGAGGG | TACGGTAGCAGAGACTTGGTCTGACTGCTTCTCACCACTTCCATT | 183 | 45 | acaggaaaattccaggtagagggacactgtctgacaggttttcacagaatcattcaggccactgtgttgagaataggctgtagggggcacaagagtacaaacaagccatttggaggctctttcaagcacttaggcaaaagatgatgaaccaaacaaaagcaatggaagtggtgagaagcagtc | Two primers sits in the repeat region |
| BRCA2 | BRCA2_Intron_24__region_1__6 | 13 | 32955922 | 32956107 | ACACTGACGACATGGTTCTACAAAACAAGCCATTTGGAGGCTCTT | TACGGTAGCAGAGACTTGGTCTTGCTTTTCTTGCAGTCTTTCCTT | 186 | 42 | aaacaagccatttggaggctctttcaagcacttaggcaaaagatgatgaaccaaacaaaagcaatggaagtggtgagaagcagtcagattcttgttgtattttgaaggtagggggacggtgcaggatggtctgaacattgggaaaaatggaattgccacttagaaggaaagactgcaagaaaagca | Two primers sits in the repeat region |
| BRCA2 | BRCA2_Intron_24__region_1__7 | 13 | 32956034 | 32956227 | ACACTGACGACATGGTTCTACAGGGACGGTGCAGGATGG | TACGGTAGCAGAGACTTGGTCTCCAGCCATTCCTCTGAACAGATT | 194 | 42 | gggacggtgcaggatggtctgaacattgggaaaaatggaattgccacttagaaggaaagactgcaagaaaagcaagtatgtggggaagttcaggagctcagttttagacagttaagttttagatgcttattaggcatctaagtagaaatgtctacttgatggttacataggaatctgttcagaggaatggctgg | Two primers sits in the repeat region |
| BRCA2 | BRCA2_Intron_24__region_2__1 | 13 | 32958725 | 32958923 | ACACTGACGACATGGTTCTACAAAGTGAGGAGGCCTAGATGTCAG | TACGGTAGCAGAGACTTGGTCTTCTGGTCCAAGACTCTGTTTTCC | 199 | 43 | AAGTGAGGAGGCCTAGATGTCAGAGGAGTCCGGCTAAACCACTGCAGAACTGCTGCCTAATTCACAGCAACCATGAGTAAAAATGCTGATGATCATCAGGTCAAGGATAGTCTGGAGCAGTTAAGATGTTACTTTACATGGGAGGTATCAATTAAAGATGATGAAATGCCTGATTTGGAAAACAGAGTCTTGGACCAGA |  |
| BRCA2 | BRCA2_Intron_24__region_2__2 | 13 | 32958830 | 32959024 | ACACTGACGACATGGTTCTACAGATAGTCTGGAGCAGTTAAGATGT | TACGGTAGCAGAGACTTGGTCTAGTTCTCCAGCACTTCCTCATTC | 195 | 41 | GATAGTCTGGAGCAGTTAAGATGTTACTTTACATGGGAGGTATCAATTAAAGATGATGAAATGCCTGATTTGGAAAACAGAGTCTTGGACCAGATTGGGTTTCTAGACTAAATACAGTGTGGGAATACACAATACACAACCTACTAGCCTATGTGAAACACCCGAAAGGCCAGAATGAGGAAGTGCTGGAGAACT |  |
| BRCA2 | BRCA2_Intron_24__region_2__3 | 13 | 32958974 | 32959172 | ACACTGACGACATGGTTCTACATAGCCTATGTGAAACACCCGAAA | TACGGTAGCAGAGACTTGGTCTGCAAATGTTCTCTACCTTGTCCA | 199 | 47 | TAGCCTATGTGAAACACCCGAAAGGCCAGAATGAGGAAGTGCTGGAGAACTTGAAAGAAGCTGAAGACTTAATCCAGAAAGAAGATGCCAATCAGATTTGAGAAGCCTGGTAACCTGGGGCAACTTTGCCTGGGTGTATTACCACATGGGCAGACTGGCAGAAACCCAGACTTACCTGGACAAGGTAGAGAACATTTGC |  |
| BRCA2 | BRCA2_Intron_24__region_2__4 | 13 | 32959058 | 32959234 | ACACTGACGACATGGTTCTACAATGCCAATCAGATTTGAGAAGCC | TACGGTAGCAGAGACTTGGTCTTCTTCCTCACAGTCCATCTCTGG | 177 | 47 | ATGCCAATCAGATTTGAGAAGCCTGGTAACCTGGGGCAACTTTGCCTGGGTGTATTACCACATGGGCAGACTGGCAGAAACCCAGACTTACCTGGACAAGGTAGAGAACATTTGCAAGAAGTTTTCAAGTCCTTTCTGTCACAGAATGGAATGTCCAGAGATGGACTGTGAGGAAGA |  |
| BRCA2 | BRCA2_Intron_24__region_2__5 | 13 | 32959147 | 32959333 | ACACTGACGACATGGTTCTACAACCTGGACAAGGTAGAGAACATT | TACGGTAGCAGAGACTTGGTCTGTGTTGAGTTCAGGGTTTTCAGC | 187 | 48 | ACCTGGACAAGGTAGAGAACATTTGCAAGAAGTTTTCAAGTCCTTTCTGTCACAGAATGGAATGTCCAGAGATGGACTGTGAGGAAGAACGGGCCTTGCTGGAATGTGGAGGGAAGAATTATGAACAGGCCAAGGCCTGCTTTGAAAAGGATCTGGCAGTGGCTGCTGAAAACCCTGAACTCAACAC |  |
| BRCA2 | BRCA2_Intron_24__region_2__6 | 13 | 32959205 | 32959401 | ACACTGACGACATGGTTCTACAGGAATGTCCAGAGATGGACTGTG | TACGGTAGCAGAGACTTGGTCTAAAATGACTTGTGATCCCCCGTT | 197 | 50 | GGAATGTCCAGAGATGGACTGTGAGGAAGAACGGGCCTTGCTGGAATGTGGAGGGAAGAATTATGAACAGGCCAAGGCCTGCTTTGAAAAGGATCTGGCAGTGGCTGCTGAAAACCCTGAACTCAACACTGGGTATGAAATCACCGCCTGTCGCCTGGATGGCTTTAAATTAGCAACGGGGGATCACAAGTCATTTT |  |
| BRCA2 | BRCA2_Intron_24__region_2__7 | 13 | 32959311 | 32959504 | ACACTGACGACATGGTTCTACAGCTGAAAACCCTGAACTCAACAC | TACGGTAGCAGAGACTTGGTCTCCTTCAGCTTCCTGTCCTTCATC | 194 | 47 | GCTGAAAACCCTGAACTCAACACTGGGTATGAAATCACCGCCTGTCGCCTGGATGGCTTTAAATTAGCAACGGGGGATCACAAGTCATTTTCTTTGCCTACCCTAAGGCAGGCTGTCAGGCTAAATGTAGATGATAGATATAGTAAGGTTCTTCTTGCCCTGAAGCTTTGGGATGAAGGACAGGAAGCTGAAGG |  |
| BRCA2 | BRCA2_Intron_24__region_3__1 | 13 | 32964498 | 32964682 | ACACTGACGACATGGTTCTACAGCGGCTCGCAAGGCTA | TACGGTAGCAGAGACTTGGTCTACAAAGAAGATTTTTAAAGGTAGGAAGCA | 185 | 32 | gcggctcgcaaggctaggagtttgggacaagtctgggcaacagtgagattctgtctctataaacataaaaaCTAAATTTAAAAAAAAAACTACTTTAAAACATAATAATAAAACAAACTGTACAAGTTCATATTCCTGATGCATGATAACATTTAAtgcttcctacctttaaaaatcttctttgt | Assays designed by relax mode and have no off-target hits |
| BRCA2 | BRCA2_Intron_24__region_3__2 | 13 | 32964614 | 32964772 | ACACTGACGACATGGTTCTACAACTGTACAAGTTCATATTCCTGATGC | TACGGTAGCAGAGACTTGGTCTAAGCATCTGACTAGGGAGGTAAT | 159 | 35 | ACTGTACAAGTTCATATTCCTGATGCATGATAACATTTAAtgcttcctacctttaaaaatcttctttgttttgcatagatgctcttttgttctccccctcttgatgaatttgtcctattcattattcagctcaagtattacctccctagtcagatgctt | One primer sits in the repeat region |
| BRCA2 | BRCA2_Intron_24__region_3__3 | 13 | 32964707 | 32964900 | ACACTGACGACATGGTTCTACACCCCCTCTTGATGAATTTGTCCT | TACGGTAGCAGAGACTTGGTCTACTGAGGCTAGTAGGTGGCTTAT | 194 | 44 | ccccctcttgatgaatttgtcctattcattattcagctcaagtattacctccctagtcagatgcttacagtctgggttagatcctccttcctccagcttttgaagcattttgttggtttggctgtttcagcacttgtcacatggtccatgatcctacctgtacacttctgaataagccacctactagcctcagt | Two primers sits in the repeat region |
| BRCA2 | BRCA2_Intron_24__region_3__4 | 13 | 32964809 | 32965000 | ACACTGACGACATGGTTCTACAAAGCATTTTGTTGGTTTGGCTGT | TACGGTAGCAGAGACTTGGTCTCCTGGAGTTTTCAACAACTGACC | 192 | 44 | aagcattttgttggtttggctgtttcagcacttgtcacatggtccatgatcctacctgtacacttctgaataagccacctactagcctcagtaatacaatgatctcttcaaggacagggactctgtttGGATTTCTACTTCCACTTCTGACATAATGGGTTGAAGCAGGGGTCAGTTGTTGAAAACTCCAGG | One primer sits in the repeat region |
| BRCA2 | BRCA2_Intron_24__region_3__5 | 13 | 32964908 | 32965094 | ACACTGACGACATGGTTCTACATGATCTCTTCAAGGACAGGGACT | TACGGTAGCAGAGACTTGGTCTTAACCTCTCTGAGCTCCAGTTTC | 187 | 44 | tgatctcttcaaggacagggactctgtttGGATTTCTACTTCCACTTCTGACATAATGGGTTGAAGCAGGGGTCAGTTGTTGAAAACTCCAGGTatgcagcatctcatacggtcctcatagtcacactgttcatttgctattatcatcccactttacagatggggaaactggagctcagagaggtta | Two primers sits in the repeat region |
| BRCA2 | BRCA2_Intron_24__region_3__6 | 13 | 32965017 | 32965207 | ACACTGACGACATGGTTCTACACGGTCCTCATAGTCACACTGTTC | TACGGTAGCAGAGACTTGGTCTTGAGTTTGCTTTTGTTTTGTCAGC | 191 | 40 | cggtcctcatagtcacactgttcatttgctattatcatcccactttacagatggggaaactggagctcagagaggttaagtagcctgctcagggtcacaatgctataaattgatttgaacttatactttcaggccctaaagctcttaggttctttcttatacttcatgctgACAAAACAAAAGCAAACTCA | Two primers sits in the repeat region |
| BRCA2 | BRCA2_Intron_24__region_3__7 | 13 | 32965060 | 32965244 | ACACTGACGACATGGTTCTACATTTACAGATGGGGAAACTGGAGC | TACGGTAGCAGAGACTTGGTCTTACAGATGTCCCTGTTTTAAGCC | 185 | 39 | tttacagatggggaaactggagctcagagaggttaagtagcctgctcagggtcacaatgctataaattgatttgaacttatactttcaggccctaaagctcttaggttctttcttatacttcatgctgACAAAACAAAAGCAAACTCAACATTTGAGAGTTGGGCTTAAAACAGGGACATCTGTA | One primer sits in the repeat region |
| BRCA2 | BRCA2_Intron_24__region_3__8 | 13 | 32965183 | 32965359 | ACACTGACGACATGGTTCTACAtgctgACAAAACAAAAGCAAACT | TACGGTAGCAGAGACTTGGTCTgaaACAGGCTTTCCCCAAAACAA | 177 | 34 | tgctgACAAAACAAAAGCAAACTCAACATTTGAGAGTTGGGCTTAAAACAGGGACATCTGTATTTTTAATCTAATGCTTTGTTACTGTATTACAGAAACACTGTGATATATAATGAGTTAATTAAACGAGAACCTTTCTTAGGTTGGGAAAGATTTGTTTTGGGGAAAGCCTGTttc | Two primers sits in the repeat region |
| BRCA2 | BRCA2_Intron_24__region_3__9 | 13 | 32965220 | 32965418 | ACACTGACGACATGGTTCTACATGGGCTTAAAACAGGGACATCTG | TACGGTAGCAGAGACTTGGTCTGCTGTTCCTAAGGGCATTTTCTA | 199 | 36 | TGGGCTTAAAACAGGGACATCTGTATTTTTAATCTAATGCTTTGTTACTGTATTACAGAAACACTGTGATATATAATGAGTTAATTAAACGAGAACCTTTCTTAGGTTGGGAAAGATTTGTTTTGGGGAAAGCCTGTttccctgggaaacagacttacagatttcatgtaggagattagaaaatgcccttaggaacagc | One primer sits in the repeat region |
| BRCA2 | BRCA2_Intron_24__region_3__10 | 13 | 32965331 | 32965527 | ACACTGACGACATGGTTCTACAAAAGATTTGTTTTGGGGAAAGCC | TACGGTAGCAGAGACTTGGTCTCAGGCTAGCAAGACTCTGAAGG | 197 | 47 | AAAGATTTGTTTTGGGGAAAGCCTGTttccctgggaaacagacttacagatttcatgtaggagattagaaaatgcccttaggaacagcacttgtgaggaagcaaatgcagcaggattgggcagaagaagaagaaaggttcagttagtcccacagggacttctggagctgggtggcccttcagagtcttgctagcctg | One primer sits in the repeat region |
| BRCA2 | BRCA2_Intron_24__region_3__11 | 13 | 32965437 | 32965630 | ACACTGACGACATGGTTCTACAGCAGCAGGATTGGGCAGAAG | TACGGTAGCAGAGACTTGGTCTCTCTGAGTTCCCTCCTGGACTTG | 194 | 56 | gcagcaggattgggcagaagaagaagaaaggttcagttagtcccacagggacttctggagctgggtggcccttcagagtcttgctagcctgaggcaagagccagtccttgcatactggctcctccagggaggggcatagccttaagcaagacaatgcctttcgacagagggcaagtccaggagggaactcagag | Two primers sits in the repeat region |
| BRCA2 | BRCA2_Intron_24__region_3__12 | 13 | 32965531 | 32965725 | ACACTGACGACATGGTTCTACACAAGAGCCAGTCCTTGCATACT | TACGGTAGCAGAGACTTGGTCTCTGGAGTGGATGCCATGGTG | 195 | 56 | caagagccagtccttgcatactggctcctccagggaggggcatagccttaagcaagacaatgcctttcgacagagggcaagtccaggagggaactcagaggtgagttgtcaatagctagctctccccagaagctggaggatcacatgccttggtcctgaaggggatctgcacccgcaccatggcatccacTCCAG | Two primers sits in the repeat region |
| BRCA2 | BRCA2_Intron_24__region_3__13 | 13 | 32965663 | 32965858 | ACACTGACGACATGGTTCTACACTGGAGGATCACATGCCTTGG | TACGGTAGCAGAGACTTGGTCTTATTTTGCAGCTTTGTGGTCTGG | 196 | 43 | ctggaggatcacatgccttggtcctgaaggggatctgcacccgcaccatggcatccacTCCAGGTGGAATCGGTGACAGTGGTTTTATAAATTGCTAAGTCCTTTATTGTTTCAGTGACATTTTCTAAAAGGAATTAAAAGCTTTGAAGAATTCTATGCAAGTTCAGAGTAGGCCAGACCACAAAGCTGCAAAATA | One primer sits in the repeat region |
| BRCA2 | BRCA2_113 | 13 | 32968741 | 32968935 | ACACTGACGACATGGTTCTACAAGGCATATTAGAGTTTCCTTTCTTGC | TACGGTAGCAGAGACTTGGTCTTGCAGCAATTAACATATGAGGCTT | 195 | 32 | AGGCATATTAGAGTTTCCTTTCTTGCATCTTAAAATTCATCTAACACATCTATAATAACATTCTTTTCTTTTTTTTCCATTCTAGGACTTGCCCCTTTCGTCTATTTGTCAGACGAATGTTACAATTTACTGGCAATAAAGTTTTGGATAGACCTTAATGAGGACATTATTAAGCCTCATATGTTAATTGCTGCA | Assays designed by relax mode and have no off-target hits |
| BRCA2 | BRCA2_114 | 13 | 32968830 | 32969028 | ACACTGACGACATGGTTCTACATGCCCCTTTCGTCTATTTGTCAG | TACGGTAGCAGAGACTTGGTCTGCCCTCTTTTGGACTAGCAGAA | 199 | 40 | TGCCCCTTTCGTCTATTTGTCAGACGAATGTTACAATTTACTGGCAATAAAGTTTTGGATAGACCTTAATGAGGACATTATTAAGCCTCATATGTTAATTGCTGCAAGCAACCTCCAGTGGCGACCAGAATCCAAATCAGGCCTTCTTACTTTATTTGCTGGAGATTTTTCTGTGTTTTCTGCTAGTCCAAAAGAGGGC | Assays designed by relax mode and have no off-target hits |
| BRCA2 | BRCA2_115 | 13 | 32968930 | 32969107 | ACACTGACGACATGGTTCTACAGCTGCAAGCAACCTCCAGT | TACGGTAGCAGAGACTTGGTCTTACCAAAATGTGTGGTGATGCTG | 178 | 41 | GCTGCAAGCAACCTCCAGTGGCGACCAGAATCCAAATCAGGCCTTCTTACTTTATTTGCTGGAGATTTTTCTGTGTTTTCTGCTAGTCCAAAAGAGGGCCACTTTCAAGAGACATTCAACAAAATGAAAAATACTGTTGAGGTAAGGTTACTTTTCAGCATCACCACACATTTTGGTA | Assays designed by relax mode and have no off-target hits |
| BRCA2 | BRCA2_116 | 13 | 32970934 | 32971113 | ACACTGACGACATGGTTCTACATGGTCCAAACTTTTCATTTCTGCTTT | TACGGTAGCAGAGACTTGGTCTGGGTGGACCACTTGGGA | 180 | 34 | TGGTCCAAACTTTTCATTTCTGCTTTTAAAGGAAATACTTTTGGAAACATAAATATGTGGGTTTGCAATTTATAAAGCAGCTTTTCCACTTATTTTCTTAGAATATTGACATACTTTGCAATGAAGCAGAAAACAAGCTTATGCATATACTGCATGCAAATGATCCCAAGTGGTCCACCC |  |
| BRCA2 | BRCA2_117 | 13 | 32971050 | 32971232 | ACACTGACGACATGGTTCTACATGCAATGAAGCAGAAAACAAGC | TACGGTAGCAGAGACTTGGTCTTGGCCTCCATATATACTTCTTATAATATTCC | 183 | 40 | TGCAATGAAGCAGAAAACAAGCTTATGCATATACTGCATGCAAATGATCCCAAGTGGTCCACCCCAACTAAAGACTGTACTTCAGGGCCGTACACTGCTCAAATCATTCCTGGTACAGGAAACAAGCTTCTGGTAAGTTAATGTAAACTCAAGGAATATTATAAGAAGTATATATGGAGGCCA |  |
| BRCA2 | BRCA2_118 | 13 | 32972189 | 32972375 | ACACTGACGACATGGTTCTACAACTGTGTGTAATATTTGCGTGCTT | TACGGTAGCAGAGACTTGGTCTGTGGAAACAGACTTCCTTTTGGC | 187 | 29 | ACTGTGTGTAATATTTGCGTGCTTAAATATTTTCAATGAAAAGTTACTTTGATTTAGTTTTTTATGTTACTACATAATTATGATAGGCTACGTTTTCATTTTTTTATCAGATGTCTTCTCCTAATTGTGAGATATATTATCAAAGTCCTTTATCACTTTGTATGGCCAAAAGGAAGTCTGTTTCCAC | Assays designed by relax mode and have no off-target hits |
| BRCA2 | BRCA2_119 | 13 | 32972289 | 32972464 | ACACTGACGACATGGTTCTACATTTTTATCAGATGTCTTCTCCTAATTGTG | TACGGTAGCAGAGACTTGGTCTCCAAGGCTCTTCTCTTTTTGCAG | 176 | 39 | TTTTTATCAGATGTCTTCTCCTAATTGTGAGATATATTATCAAAGTCCTTTATCACTTTGTATGGCCAAAAGGAAGTCTGTTTCCACACCTGTCTCAGCCCAGATGACTTCAAAGTCTTGTAAAGGGGAGAAAGAGATTGATGACCAAAAGAACTGCAAAAAGAGAAGAGCCTTGG | Assays designed by relax mode and have no off-target hits |
| BRCA2 | BRCA2_120 | 13 | 32972412 | 32972609 | ACACTGACGACATGGTTCTACAAGGGGAGAAAGAGATTGATGACC | TACGGTAGCAGAGACTTGGTCTGGAGAATTCAGTTCTTTTTTCTTTATGGG | 198 | 43 | AGGGGAGAAAGAGATTGATGACCAAAAGAACTGCAAAAAGAGAAGAGCCTTGGATTTCTTGAGTAGACTGCCTTTACCTCCACCTGTTAGTCCCATTTGTACATTTGTTTCTCCGGCTGCACAGAAGGCATTTCAGCCACCAAGGAGTTGTGGCACCAAATACGAAACACCCATAAAGAAAAAAGAACTGAATTCTCC | Assays designed by relax mode and have no off-target hits |
| BRCA2 | BRCA2_121 | 13 | 32972486 | 32972684 | ACACTGACGACATGGTTCTACATACCTCCACCTGTTAGTCCCATT | TACGGTAGCAGAGACTTGGTCTGCAAGTTCTTCGTCAGCTATTGA | 199 | 39 | TACCTCCACCTGTTAGTCCCATTTGTACATTTGTTTCTCCGGCTGCACAGAAGGCATTTCAGCCACCAAGGAGTTGTGGCACCAAATACGAAACACCCATAAAGAAAAAAGAACTGAATTCTCCTCAGATGACTCCATTTAAAAAATTCAATGAAATTTCTCTTTTGGAAAGTAATTCAATAGCTGACGAAGAACTTGC | Assays designed by relax mode and have no off-target hits |
| BRCA2 | BRCA2_122 | 13 | 32972573 | 32972771 | ACACTGACGACATGGTTCTACAACGAAACACCCATAAAGAAAAAAGAACT | TACGGTAGCAGAGACTTGGTCTGTGGGAGCAGTCCTAGTGGAT | 199 | 35 | ACGAAACACCCATAAAGAAAAAAGAACTGAATTCTCCTCAGATGACTCCATTTAAAAAATTCAATGAAATTTCTCTTTTGGAAAGTAATTCAATAGCTGACGAAGAACTTGCATTGATAAATACCCAAGCTCTTTTGTCTGGTTCAACAGGAGAAAAACAATTTATATCTGTCAGTGAATCCACTAGGACTGCTCCCAC | Assays designed by relax mode and have no off-target hits |
| BRCA2 | BRCA2_123 | 13 | 32972696 | 32972846 | ACACTGACGACATGGTTCTACACCCAAGCTCTTTTGTCTGGTTC | TACGGTAGCAGAGACTTGGTCTGCCTGGGAACTCTCCTGTTCT | 151 | 44 | CCCAAGCTCTTTTGTCTGGTTCAACAGGAGAAAAACAATTTATATCTGTCAGTGAATCCACTAGGACTGCTCCCACCAGTTCAGAAGATTATCTCAGACTGAAACGACGTTGTACTACATCTCTGATCAAAGAACAGGAGAGTTCCCAGGC |  |
| BRCA2 | BRCA2_124 | 13 | 32972756 | 32972954 | ACACTGACGACATGGTTCTACACTAGGACTGCTCCCACCA | TACGGTAGCAGAGACTTGGTCTACTGGAAAGGTTAAGCGTCAATA | 199 | 40 | CTAGGACTGCTCCCACCAGTTCAGAAGATTATCTCAGACTGAAACGACGTTGTACTACATCTCTGATCAAAGAACAGGAGAGTTCCCAGGCCAGTACGGAAGAATGTGAGAAAAATAAGCAGGACACAATTACAACTAAAAAATATATCTAAGCATTTGCAAAGGCGACAATAAATTATTGACGCTTAACCTTTCCAGT | Assays designed by relax mode and have no off-target hits |
| BRCA2 | BRCA2_3_UTR_1 | 13 | 32972832 | 32972989 | ACACTGACGACATGGTTCTACAGGAGAGTTCCCAGGCCAGTA | TACGGTAGCAGAGACTTGGTCTAATGTGTGGTTTGAAATTATATTCCAGT | 158 | 35 | GGAGAGTTCCCAGGCCAGTACGGAAGAATGTGAGAAAAATAAGCAGGACACAATTACAACTAAAAAATATATCTAAGCATTTGCAAAGGCGACAATAAATTATTGACGCTTAACCTTTCCAGTTTATAAGACTGGAATATAATTTCAAACCACACATT |  |
| BRCA2 | BRCA2_3_UTR_2 | 13 | 32972934 | 32973106 | ACACTGACGACATGGTTCTACATTGACGCTTAACCTTTCCAGTTT | TACGGTAGCAGAGACTTGGTCTTGCAACTGAAGCAAAAGTATACCA | 173 | 35 | TTGACGCTTAACCTTTCCAGTTTATAAGACTGGAATATAATTTCAAACCACACATTAGTACTTATGTTGCACAATGAGAAAAGAAATTAGTTTCAAATTTACCTCAGCGTTTGTGTATCGGGCAAAAATCGTTTTGCCCGATTCCGTATTGGTATACTTTTGCTTCAGTTGCA |  |
| BRCA2 | BRCA2_3_UTR_3 | 13 | 32973032 | 32973229 | ACACTGACGACATGGTTCTACATTACCTCAGCGTTTGTGTATCGG | TACGGTAGCAGAGACTTGGTCTCTGGCCTCAAGCACTCCTC | 198 | 42 | TTACCTCAGCGTTTGTGTATCGGGCAAAAATCGTTTTGCCCGATTCCGTATTGGTATACTTTTGCTTCAGTTGCATATCTTAAAACTAAATGTAATTTATTAACTAATCAAGAAAAACATCTTTggctgagctcggtggctcatgcctgtaatcccaacactttgagaagctgaggtgggaggagtgcttgaggccag | One primer sits in the repeat region |
| BRCA2 | BRCA2_3_UTR_5 | 13 | 32973253 | 32973427 | ACACTGACGACATGGTTCTACACATAGGGAGACCCCCATCTT | TACGGTAGCAGAGACTTGGTCTTCTGCATCAAAATAACTGTACTAAGAGA | 175 | 30 | catagggagacccccatctttacaaagaaaaaaaaaaGGGGAAAAGAAAATCTTTTAAATCTTTGGATTTGATCACTACAAGTATTATTTTACAAGTGAAATAAACATACCATTTTCTTTTAGATTGTGTCATTAAATGGAATGAGGTCTCTTAGTACAGTTATTTTGATGCAGA | One primer sits in the repeat region |
| BRCA2 | BRCA2_3_UTR_6 | 13 | 32973316 | 32973499 | ACACTGACGACATGGTTCTACATGGATTTGATCACTACAAGTATTATTTTACA | TACGGTAGCAGAGACTTGGTCTAGGAGAACTATTTCATAGTGAGTTACC | 184 | 29 | TGGATTTGATCACTACAAGTATTATTTTACAAGTGAAATAAACATACCATTTTCTTTTAGATTGTGTCATTAAATGGAATGAGGTCTCTTAGTACAGTTATTTTGATGCAGATAATTCCTTTTAGTTTAGCTACTATTTTAGGGGATTTTTTTTAGAGGTAACTCACTATGAAATAGTTCTCCT |  |
| BRCA2 | BRCA2_3_UTR_7 | 13 | 32973390 | 32973583 | ACACTGACGACATGGTTCTACATGGAATGAGGTCTCTTAGTACAGTT | TACGGTAGCAGAGACTTGGTCTTGCTCAAAAGGAAACACCACTCT | 194 | 34 | TGGAATGAGGTCTCTTAGTACAGTTATTTTGATGCAGATAATTCCTTTTAGTTTAGCTACTATTTTAGGGGATTTTTTTTAGAGGTAACTCACTATGAAATAGTTCTCCTTAATGCAAATATGTTGGTTCTGCTATAGTTCCATCCTGTTCAAAAGTCAGGATGAATATGAAGAGTGGTGTTTCCTTTTGAGCA |  |
| BRCA2 | BRCA2_3_UTR_8 | 13 | 32973511 | 32973681 | ACACTGACGACATGGTTCTACATGTTGGTTCTGCTATAGTTCCATCC | TACGGTAGCAGAGACTTGGTCTTTTAATTTCAGAGATCACACTGGAATAGT | 171 | 35 | TGTTGGTTCTGCTATAGTTCCATCCTGTTCAAAAGTCAGGATGAATATGAAGAGTGGTGTTTCCTTTTGAGCAATTCTTCATCCTTAAGTCAGCATGATTATAAGAAAAATAGAACCCTCAGTGTAACTCTAATTCCTTTTTACTATTCCAGTGTGATCTCTGAAATTAAA |  |
| BRCA2 | BRCA2_3_UTR_9 | 13 | 32973572 | 32973770 | ACACTGACGACATGGTTCTACATCCTTTTGAGCAATTCTTCATCCTT | TACGGTAGCAGAGACTTGGTCTTGAGTTTGGATGACCATTTTGTTG | 199 | 27 | TCCTTTTGAGCAATTCTTCATCCTTAAGTCAGCATGATTATAAGAAAAATAGAACCCTCAGTGTAACTCTAATTCCTTTTTACTATTCCAGTGTGATCTCTGAAATTAAATTACTTCAACTAAAAATTCAAATACTTTAAATCAGAAGATTTCATAGTTAATTTATTTTTTTTTTCAACAAAATGGTCATCCAAACTCA |  |
| BRCA2 | BRCA2_3_UTR_10 | 13 | 32973633 | 32973831 | ACACTGACGACATGGTTCTACATGTAACTCTAATTCCTTTTTACTATTCCAGT | TACGGTAGCAGAGACTTGGTCTGCGCTAAAAATAAAGCAGGCAGA | 199 | 28 | TGTAACTCTAATTCCTTTTTACTATTCCAGTGTGATCTCTGAAATTAAATTACTTCAACTAAAAATTCAAATACTTTAAATCAGAAGATTTCATAGTTAATTTATTTTTTTTTTCAACAAAATGGTCATCCAAACTCAAACTTGAGAAAATATCTTGCTTTCAAATTGGCACTGATTCTGCCTGCTTTATTTTTAGCGC |  |
| BRCA2 | BRCA2_3_UTR_11 | 13 | 32973746 | 32973929 | ACACTGACGACATGGTTCTACATCAACAAAATGGTCATCCAAACTCAA | TACGGTAGCAGAGACTTGGTCTaaaaaaGGACGTAACAAATGAGAGTAT | 184 | 35 | TCAACAAAATGGTCATCCAAACTCAAACTTGAGAAAATATCTTGCTTTCAAATTGGCACTGATTCTGCCTGCTTTATTTTTAGCGCTATCACAGGACCCAGAGCCTATGCCCTTTTAAACTTACCACAAAAGCAGAAGATTAATTCAATTTAAGATGATACTCTCATTTGTTACGTCCtttttt | One primer sits in the repeat region |
| BRCA2_3_UTR | BRCA2_3_UTR_12 | 13 | 32973158 | 32973307 | ACACTGACGACATGGTTCTACACTGAGCTCGGTGGCTCAT | TACGGTAGCAGAGACTTGGTCTAAAGATTTTCTTTTCCCCTTTTT | 150 | 49 | CTGAGCTCGGTGGCTCATGCCTGTAATCCCAACACTTTGAGAAGCTGAGGTGGGAGGAGTGCTTGAGGCCAGGAGTTCAAGACCAGCCTGGGCAACATAGGGAGACCCCCATCTTTACAAAGAAAAAAAAAAGGGGAAAAGAAAATCTTT |  |
| BRCA2_Promoter_Combined | BRCA2_Promoter_Combined_14 | 13 | 32888661 | 32888849 | ACACTGACGACATGGTTCTACACTGCGAGGAAGACAGGTGAT | TACGGTAGCAGAGACTTGGTCTGCACGGCTAACTTTGCATTT | 189 | 55 | CTGCGAGGAAGACAGGTGATCCGAATCCTAAGAATGCAAAAGATGGGCCGGGTGTGGTGGCTCATGCCTGTAATCCCAGCGCTTTGGGAGGCCGAGGCAGGCAGATCACCTGAGGTCGGGAGGTTGAGACCAGACTGACCAACAACGGAGAAACCCCGTCTCTACTTAAAAATGCAAAGTTAGCCGTGC |  |
| BRCA2_Promoter_Combined | BRCA2_Promoter_Combined_15 | 13 | 32888792 | 32888990 | ACACTGACGACATGGTTCTACAAGACTGACCAACAACGGAGAA | TACGGTAGCAGAGACTTGGTCTTGAGACGGAGTTTCGCTCTT | 199 | 57 | AGACTGACCAACAACGGAGAAACCCCGTCTCTACTTAAAAATGCAAAGTTAGCCGTGCGTGGTGGCCCATGCCTGTATTCCCAGCTACTCGGGAGGCTGAGGCAGGAGAACCACTTGATCCCTGGAGGCGGAAGTTGCGGTGAGCGGAGATTGCGCCATTGCACACCAGCCCGGGCCACAAGAGCGAAACTCCGTCTCA |  |
| BRCA2_Promoter_Combined | BRCA2_Promoter_Combined_16 | 13 | 32888925 | 32889092 | ACACTGACGACATGGTTCTACAGTTGCGGTGAGCGGAGAT | TACGGTAGCAGAGACTTGGTCTCCGTTTCCTTGCTTGAAAAA | 168 | 50 | GTTGCGGTGAGCGGAGATTGCGCCATTGCACACCAGCCCGGGCCACAAGAGCGAAACTCCGTCTCAAAAAAAAAAGCAAAAGATACTACCAAGCCCTGCGGAGCAAGGTACCTCACACTTCATGAGCGAGTTAAGATGGGTTTCACAATTTTTCAAGCAAGGAAACGG |  |
